# Enantiopure Dinaphtho[2,3‐*b*:2,3‐*f*]thieno[3,2‐*b*]thiophenes: Reaching High Magnetoresistance Effect in OFETs

**DOI:** 10.1002/advs.202301914

**Published:** 2023-07-09

**Authors:** Martina Volpi, Rémy Jouclas, Jie Liu, Guangfeng Liu, Luca Catalano, Nemo McIntosh, Marco Bardini, Christos Gatsios, Federico Modesti, Nicholas Turetta, David Beljonne, Jérôme Cornil, Alan R. Kennedy, Norbert Koch, Peter Erk, Paolo Samorì, Guillaume Schweicher, Yves H. Geerts

**Affiliations:** ^1^ Laboratoire de Chimie des Polymères Faculté des Sciences Université Libre de Bruxelles (ULB) Boulevard du Triomphe, CP 206/01 Bruxelles 1050 Belgium; ^2^ Laboratory for Chemistry of Novel Materials Center for Research in Molecular Electronics and Photonics University of Mons Place du Parc 23 Mons B‐7000 Belgium; ^3^ Helmholtz‐Zentrum Berlin für Materialien und Energie GmbH 12489 Berlin Germany; ^4^ Institut für Physik and IRIS Adlershof Humboldt‐Universitat zu Berlin 12489 Berlin Germany; ^5^ BASF SE RGD – J542S 67056 Ludwigshafen am Rhein Germany; ^6^ CNRS University of Strasbourg ISIS UMR 7006, 8 Alleé Gaspard Monge Strasbourg F‐67000 France; ^7^ Department of Pure and Applied Chemistry University of Strathclyde Cathedral Street 295 Glasgow G1 1XL UK; ^8^ International Solvay Institutes for Physics and Chemistry Université Libre de Bruxelles (ULB) Boulevard du Triomphe, CP 231 Bruxelles 1050 Belgium

**Keywords:** chiral induced spin selectivity effect, chirality, magnetoresistance, organic semiconductors, spin, transistors

## Abstract

Chiral molecules are known to behave as spin filters due to the chiral induced spin selectivity (CISS) effect. Chirality can be implemented in molecular semiconductors in order to study the role of the CISS effect in charge transport and to find new materials for spintronic applications. In this study, the design and synthesis of a new class of enantiopure chiral organic semiconductors based on the well‐known dinaphtho[2,3‐*b*:2,3‐*f*]thieno[3,2‐*b*]thiophene (DNTT) core functionalized with chiral alkyl side chains is presented. When introduced in an organic field‐effect transistor (OFET) with magnetic contacts, the two enantiomers, (*R*)‐DNTT and (*S*)‐DNTT, show an opposite behavior with respect to the relative direction of the magnetization of the contacts, oriented by an external magnetic field. Each enantiomer displays an unexpectedly high magnetoresistance over one preferred orientation of the spin current injected from the magnetic contacts. The result is the first reported OFET in which the current can be switched on and off upon inversion of the direction of the applied external magnetic field. This work contributes to the general understanding of the CISS effect and opens new avenues for the introduction of organic materials in spintronic devices.

## Graphical abstract

The first example of a chiral organic field‐effect transistor (OFET) is reported in which the current can be switched on and off upon inversion of the direction of an external magnetic field. This work contributes to the general understanding of the chiral induced spin selectivity (CISS) effect and opens new avenues for the introduction of organic materials in spintronic devices.

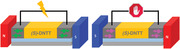

## 1 Introduction

Organic semiconductors (OSCs) are solution‐processable, lightweight, and multifunctional materials that find applications in organic field‐effect transistors (OFETs). A great effort has been devoted over the past decades to the design and synthesis of new OSCs displaying high charge carrier mobility (*µ*), optimal device characteristics, and performances that could compete with silicon in large area electronics. Thanks to the versatility of organic synthesis, it is easy to tune material properties through a careful molecular design and the engineering of crystal packing. Molecular design relies on new conjugated cores and the introduction of side chains facilitating solubility and dense crystal packing, in order to increase the processability of the material and the efficiency of charge transport.

Among all the possible molecular and material properties available to synthetic chemists, chirality is still barely exploited in the field of OSCs.^[^ ^1^ ^]^ At the molecular level, chirality is a geometric property implying that enantiomers cannot be superimposed to their mirror image. Chiral molecules have been demonstrated to behave as spin filters due to the chiral induced spin selectivity (CISS) effect, experimentally proved for a large variety of organic and inorganic systems.^[^ ^2^, ^3^, ^4^, ^5^, ^6^, ^7^, ^8^, ^9^, ^10^ ^]^ Electrons with either spin up or down cross enantiomers at different rates depending on molecular handedness, though it is not yet understood whether this selectivity arises at the charge injection level or when the charges drift over the molecular backbone. Although featuring a much lower strength, the electrical magnetochiral anisotropy (eMChA) is closely related to CISS effect, because it implies a variation of the electrical resistance of the sample depending on the interplay between chirality and magnetic fields.^[^ ^11^ ^]^ However, eMChA scales with the magnetic field whereas CISS does not.^[^ ^12^, ^13^, ^14^, ^15^, ^16^ ^]^ The origin and theoretical explanations of the CISS effect are debated. Different theories have been proposed to describe it, but to date, a quantitative agreement has not been reached.^[^ ^3^, ^17^ ^]^ The handedness of the material couples with the spin of the electron resulting in the preferential transmission of one spin and in the reflection of the opposite one. An open question is to know whether the molecules act as spin filters or as spin polarizers; in the first case the transmitted and reflected electrons would have opposite spins, while in the second transmitted and reflected electrons would present the same spin orientation.^[^ ^18^ ^]^

Chiral systems raised considerable interest in the field of spintronics, since chiral and helical structures have been proved to behave as spin filters in different spintronic devices.^[^ ^19^ ^]^ Spintronics or spin electronics is a quantum technology that aims to add the spin quantum degree of freedom to conventional electronic devices.^[^ ^20^ ^]^ At the beginning, spintronic was relying only on metals and inorganic materials.^[^ ^21^ ^]^ The first use of a π‐conjugated molecule in spintronics dates back to 2004 with the generation of a hybrid organic/inorganic spin valve in which the current fluctuates when switching the magnetization of the two contacts from parallel to opposite.^[^ ^22^ ^]^ The interest in organic molecules for spintronic applications is related to the weak spin‐orbit coupling implying low spin scattering and long spin lifetime, which are ideal conditions to carry the spin information over a long length and time scale.^[^ ^23^, ^24^ ^]^ In spintronic devices, the spin‐dependent hybridization at interfaces between ferromagnetic electrodes and molecular materials, named spinterface, can also play a major role in defining device performances. For instance, the strong interaction between the metal and chemisorbed molecules at the spinterface can induce a spin polarization within the molecule; in extreme cases, the resulting molecular spin polarization can be opposite to the spin of majority carriers at the Fermi level in the ferromagnetic contact, thus leading to a huge contact resistance.^[^ ^25^ ^]^ The role of the spinterface has also been studied in the frame of the CISS effect in single‐molecule junctions.^[^ ^26^, ^27^ ^]^

To the best of our knowledge, the coupling of chiral molecules, CISS effect, and spintronic devices has mainly been exploited in spin valves and Hall sensors. Magnetoresistance have been measured in four probe devices and in molecule/ferromagnet bilayer systems for various organic systems of opposite handedness, such as DNA, superhelical polyaniline microfibers, oligopeptides, supramolecular polymers, and metal–organic complexes.^[^ ^28^, ^29^, ^30^, ^31^, ^32^ ^]^ Noteworthy, a recent paper focused on the measurement of CISS effect in a spin transistor.^[^ ^33^ ^]^ Other publications deal with the use of chiral semiconductors in OFETs but without mention of CISS effect.^[^ ^34^, ^35^, ^36^, ^37^, ^38^, ^39^, ^40^, ^41^, ^42^ ^]^ For the sake of completeness, it should be noted that magnetic effects in achiral semiconductors are known to be very weak.^[^ ^43^ ^]^

Here, we report the design, synthesis, and characterization of enantiopure semiconductors with the goal of studying CISS effect in OFETs, aiming at contributing to its understanding. The density of charge and spin carriers is orders of magnitude higher in OFETs than in two terminal devices, and charges are transported over distances that exceed 100 µm, compared to the 1–10 nm prevailing in diodes.

## 2 Results and Discussion

### 2.1 Design, Synthesis, and Characterization

**Figure 1:**
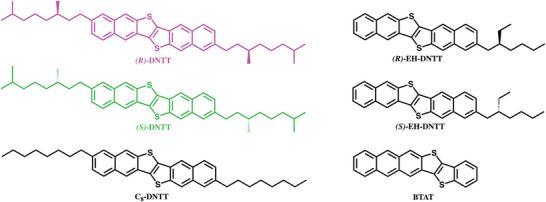
Molecular structure of (*R*)‐DNTT (pink), (*S*)‐DNTT (green), C8‐DNTT, (*R*)‐EH‐DNTT, (*S*)‐EH‐DNTT, and BTAT.

Our molecular design starts with the selection of dinaphtho[2,3‐*b*:2,3‐*f*]thieno[3,2‐*b*]thiophene (DNTT) as aromatic core because it qualifies as one of the best performing OSCs.^[^ ^44^ ^]^ Two alkyl side chains bearing a stereogenic center and derived from citronellol, for which both enantiomers are commercially available, are linked to the DNTT core at 2 and 9 positions. (*R*)‐DNTT and (*S*)‐DNTT, depicted in **Figure** **1**, are structurally similar to C_8_‐DNTT that is well known for both exhibiting mobilities in excess of 10 cm^2^ V^−1^ s^−1^ and an ideal Hall effect response in single crystal devices.^[^ ^45^, ^46^, ^47^ ^]^ We anticipate that the methyl group at the 3 position on the side chain is sufficiently close to the π‐system for imparting a chiral arrangement of the DNTT cores in the crystal structure, while preserving the herringbone (HB) packing known for alkylated DNTTs.^[^ ^48^ ^]^ This is of great importance because the relative arrangement of π‐systems determines the overlap of the frontier orbitals responsible for the charge transport and therefore the amplitudes, the distribution and the 2D‐isotropicity of the transfer integrals.^[^ ^49^ ^]^

The two enantiomers, (*R*)‐DNTT and (*S*)‐DNTT, have been obtained by following the synthetic route of alkylated DNTT reported by Takimiya et al.^[^ ^50^ ^]^ The only variation on the reported synthesis concerns the introduction of the alkyl chain on the aromatic system (Figure S1, Supporting Information). The chiral chains used as starting materials in the synthesis are commercially available with an enantiomeric excess (*ee*) of 99% for (*S*)‐citronellol and of 95% for (*R*)‐citronellyl bromide. To minimize the consumption of (*R*)‐ or (*S*)‐8‐bromo‐2,6‐dimethyloct‐2‐ene, we performed an iron‐catalyzed alkylation of the aromatic Grignard,^[^ ^51^ ^]^ where the chiral starting compounds are the limiting reagents, instead of the Kumada cross‐coupling reported by Takimiya et al., where the *n*‐alkyl Grignard reagents are used in excess. The two enantiomers have been synthesized with an overall yield of 18% and fully characterized (Section S1, Supporting Information). Since the final product of the synthesis couples two alkyl chains to the DNTT core, the *ee* of the target enantiomers is lower compared to the starting chiral chains, due to the presence of traces of the other enantiomer and of the meso‐compound (*R*,*S*)‐DNTT (Table S1, Supporting Information). Only an *ee* approaching 100% is tolerable because charge transport is very sensitive to structural defects. To this end, several recrystallization steps in toluene were performed until a constant melting point, identical for both enantiomers, is reached.^[^ ^52^ ^]^ Only four steps suffice for affording (*R*)‐DNTT and (*S*)‐DNTT exhibiting the same and constant melting point of 254.8 °C, with an enantiopurification yield of 82% for both enantiomers (Figure S23, Supporting Information, Supporting Information).

### 2.2 Thermal, Structural, and Spectroscopic Properties

**Figure 2:**
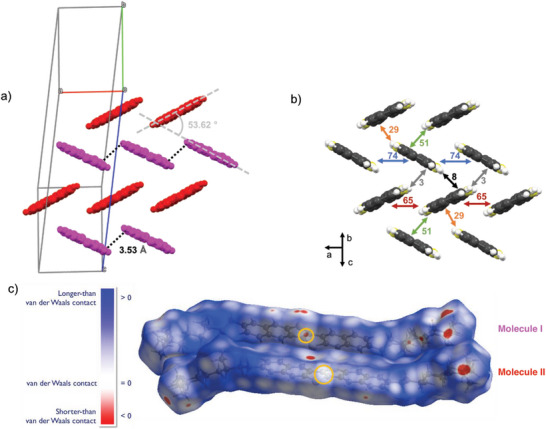
a) Crystal packing diagram of (*S*)‐DNTT in the herringbone plane without the chiral alkyl chains. Molecules in pink and red are crystallographically independent one with respect to the other. The black dashed lines represent S–S short contacts, the herringbone angle is displayed in grey. b) (*S*)‐DNTT intermolecular transfer integrals (in meV) calculated from the experimental crystal structure. c) Front view of the Hirshfeld surfaces of the two distinct molecules belonging to the same unit cell of (*S*)‐DNTT with S–S contact regions highlighted in yellow.

The intermediates of the synthesis were purified and characterized by common methods (see Section S1, Supporting Information). It is a textbook concept that enantiomers have identical chemical and physical properties, and it is possible to distinguish between them, only in the presence of another chiral object (for example a chiral molecule or circularly polarized light). For this reason, most of the chemical, physical, and structural characterizations are reported solely for one of the two enantiomers. Proton NMR (Section S1.2, Supporting Information) and UV–vis absorption spectra (Figure S22, Supporting Information) of (*R*)‐DNTT and (*S*)‐DNTT are in perfect agreement with the spectra of C_8_‐DNTT already reported in literature.^[^ ^50^ ^]^ The final proof of the structure and enantiopurity of the products comes from the solution of the crystal structure obtained by single‐crystal X‐ray diffraction (SCXRD). Single crystals of (*S*)‐DNTT have been grown over a month at room temperature from a saturated solution in chlorobenzene, by slow diffusion of dichloromethane as antisolvent. The crystal structure that has been solved at room temperature, belongs to the triclinic space group *P_1_* and presents two crystallographically unique molecules in the unit cell (Section S5.1, Supporting Information). This lack of symmetry is rather uncommon for thienoacene‐based semiconductors and it is shared only by the non‐symmetric BTAT and the chiral EH‐DNTT reported by Takimiya et al. (Figure 1).^[^ ^41^, ^53^ ^]^ The chiral side chains of (*S*)‐DNTT are disordered in the crystal structure (Figure S35, Supporting Information), while the DNTT cores are perfectly ordered in the desired HB packing (**Figure** **2**a), with a HB angle of 53.7°. Crystallographic data confirm that the chiral side chains have not racemized during the synthesis. Interestingly only one of the two molecules in the unit cell makes S–S contacts shorter than the sum of the sulfur van der Waals (VdW) radius (3.60 Å), for a distance of 3.53 Å (highlighted in black in Figure 2a). This difference in intermolecular contacts is highlighted also in the Hirshfeld surfaces calculated on the two molecules of the asymmetric unit (Figure 2c). S–S contacts in Molecule I are represented as a red spot with a distance shorter than VdW contacts, while in Molecule II S–S contacts are represented by a white area corresponding to a distance equal to the sum of VdW contacts. The two molecules have a different packing environment and are characterized by different short contacts regions, even though fingerprint plots calculated from Hirshfeld surfaces are almost identical (Figure S36, Supporting Information). The chemical purity of the final products has been also assessed by X‐ray photoelectron spectroscopy measurements, confirming the correct C/S ratio of 21 for both enantiomers (Section S2.1, Supporting Information).

The thermal stability of the products has been analyzed by different methods (Section S4, Supporting Information). Thermogravimetric analysis demonstrates that (*R*)‐DNTT and (*S*)‐DNTT are thermally stable up to ≈370 °C (Figure S29, Supporting Information). Differential scanning calorimetry traces indicate that (*R*)‐DNTT and (*S*)‐DNTT exhibit three polymorphic forms (Cr_A_, Cr_B_, and Cr_C_). The heating traces (Figures S30 and S31, Supporting Information) successively show a weak transition (Cr_A_→Cr_B_) around 80 °C, followed by a more intense one (Cr_B_→Cr_C_) around 215 °C. A last transition (Cr_C_→L) corresponding to the melting is observed at 254.8 °C, for both (*R*)‐DNTT and (*S*)‐DNTT. Variable temperature powder X‐ray diffraction (PXRD) patterns (Figure S34, Supporting Information) and optical microscopy images (Figure S33, Supporting Information) assess that none of the phases are liquid crystalline. Specifically, crystals with sharply defined angles are observed until the two enantiomers melt. A gradual shift toward higher 2*θ* values of the first three 00*l* diffraction peaks upon increasing the temperature from 190 to 240 °C indicates a contraction of the unit cell with temperature of up to 2 Å along this specific crystallographic direction. The 002 peak, in the pattern recorded at room temperature, corresponds to a distance of 33 Å, which equates the length of the molecule with straight alkyl chains, in agreement with the 33.2 Å distance given by the structure solved by SCXRD. The PXRD patterns of (*R*)‐DNTT and (*S*)‐DNTT recorded at 25 °C are in agreement with the PXRD pattern calculated from the SCXRD data, also registered at 25 °C (Figure S38, Supporting Information). The ionization energy (IE) of (*R*)‐DNTT and (*S*)‐DNTT measured by photoemission yield spectroscopy (Section S3, Supporting Information) are respectively 5.23 (±0.01) and 5.19 (±0.01). These IE values are consistent with those measured by ultraviolet photoelectron spectroscopy, which gives 5.16 (±0.06) and 5.18 (±0.06) eV for (*R*)‐DNTT and (*S*)‐DNTT, respectively (Section S2.2, Supporting Information). We thus expect an efficient charge injection from Au in OFETs, similarly to C_10_‐DNBDT (5.2 eV), DN4T (5.3 eV), DNTT, and DNBDT (5.4 eV).^[^ ^50^, ^54^, ^55^ ^]^

### 2.3 Quantum‐Chemical Calculations

The shape of the highest occupied molecular orbital (HOMO) of (*S*)‐DNTT (Figure S40, Supporting Information) has been calculated by density functional theory, after geometry optimization in gas phase at the B3LYP/6‐311G* level with the Gaussian package.^[^ ^56^ ^]^ The transfer integrals between HOMOs of interacting molecules were computed from the experimental crystal structure with the B3LYP functional and a DZ basis set within ADF,^[^ ^57^ ^]^ using a fragment orbital approach. The HOMO has the same shape and spatial distribution as DNTT and alkylated DNTT, as expected since the orbital is localized only on the DNTT core and is not affected by the presence of the chiral chains.^[^ ^44^, ^58^ ^]^

The spatial distribution of transfer integrals reported in Figure 2b is unusually anisotropic and asymmetric compared to similar thienoacene structures.^[^ ^1^ ^]^ It is also interesting to note that the highest transfer integrals (74 meV) correspond to dimers characterized by the S–S shortest contacts.

### 2.4 Charge and Spin Transport Measurements

**Figure 3:**
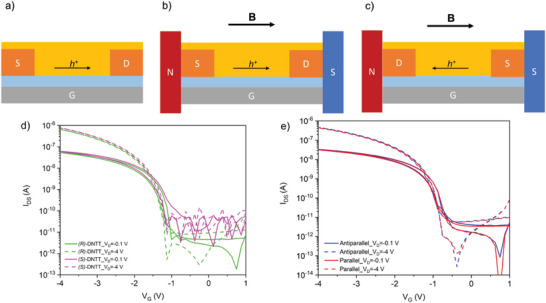
a) Geometry of a BGBC device with Au electrodes, b) representation of a BC device in a parallel magnetic field, and c) in an antiparallel magnetic field. Gate contact (G) is depicted in gray, the dielectric in light blue, source (S) and drain (D) gold contacts in orange and the semiconductor in yellow. The direction of the magnetic field (B) and of the hole current (h^+^) are depicted with black arrows. d) Transfer curves of (*R*)‐DNTT (green) and (*S*)‐DNTT (pink) best performing OFETs fabricated with BGBC geometry at a substrate temperature of 40 °C. e) Transfer curves of (*S*)‐DNTT BC OFETs placed in a parallel (red) and antiparallel (blue) magnetic field.

To investigate charge transport properties of chiral DNTTs we fabricated organic field‐effect transistors with bottom‐gate bottom‐contact (BGBC) geometry (**Figure** **3**a). Side chains of organic semiconductors are known to hinder efficient spin injection.^[^ ^59^ ^]^ BGBC transistors are preferable for efficient spin injection from electrodes directly in contact with aromatic cores. Devices consist of polycrystalline thin films of the material evaporated under high vacuum on silicon substrates with an Al_2_O_3_ dielectric layer held at various temperatures during the OSCs deposition (Section S7.1, Supporting Information). As expected with source and drain Au electrodes, (*S*)‐DNTT and (*R*)‐DNTT have similar values of mobility ranging between 0.5 and 0.6 cm^2^ V^−1^ s^−1^, threshold voltages (*V*_th_) between −1.4 and −1.6 V and ON/OFF ratio of 2 × 10^4^, in the linear regime. All devices show an ideal behavior and comparable electrical performances. The best performing devices were obtained at a substrate temperature of 40 °C (Figure 3d) (Table S7, Supporting Information), exhibiting mobilities up to 0.52 and 0.57 cm^2^ V^−1^ s^−1^ and threshold voltages of −1.51 and −1.53 V, in the linear regime for (*R*)‐DNTT and (*S*)‐DNTT, respectively. The morphologies of thin films evaporated at different substrate temperatures were characterized by XRD and AFM (Section S7.2, Supporting Information), to investigate the origin of the decrease in mobility in devices evaporated at substrate temperatures higher than 40 °C. XRD patterns (Figure S43, Supporting Information) show no polymorphism upon increasing the substrate temperature from 40 to 130 °C. AFM topographies at different substrate temperatures (Figure S46, Supporting Information) exhibit different film morphologies, different domain sizes, and different crystallinity, all factors that can influence the mobility and device performances.

To probe eMChA, we have placed (*S*)‐DNTT BC transistors with Au contacts in parallel (Figure 3b) and antiparallel (Figure 3c) magnetic field of 0.2 T. The external magnetic field was applied by two permanent neodymium magnets placed parallel to each other at two opposite sides of the substrate (Figure S46a, Supporting Information). As clearly shown in Figure 3e, we found no difference in the device response upon inversion of the magnetic field direction. No eMChA has been observed on charge transport when injecting non polarized charges from non‐ferromagnetic contacts, indicating no measurable spin filtering or CISS effect by (*S*)‐DNTT.

**Figure 4:**
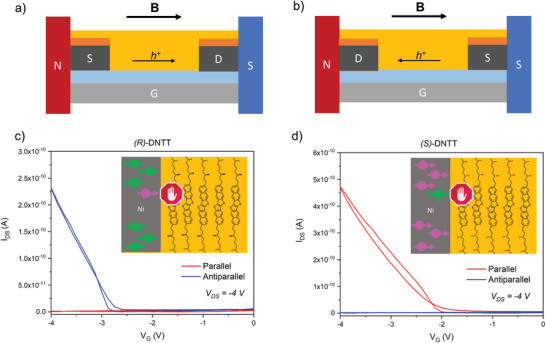
Geometry of a BGBC device with ferromagnetic electrodes a) in a parallel and b) in an antiparallel magnetic field (B). Gate contact (G) is depicted in gray, the dielectric in light blue, source (S) and drain (D) contacts in orange (Au) and dark grey (Ni) and the semiconductor in yellow. The direction of the magnetic field (B) and of the hole current (h^+^) are depicted with black arrows. c) Transfer curves in saturation regime (*V*_d_ = −4 V) of (*R*)‐DNTT and d) (*S*)‐DNTT in a parallel (red) and antiparallel (blue) magnetic field. In the graph is reported a schematic representation of the injection of parallel (pink) or antiparallel (green) charges from the ferromagnetic contact (grey area) into the chiral semiconductor (yellow area).

To probe the CISS effect in presence of a spin‐polarized current we built BC devices using ferromagnets as contacts. The ferromagnetic contacts are made of Ni (40 nm), covered by a thin layer of gold (10 nm) to protect Ni from oxidation and to ease charge injection in the semiconductor. The two metals are successively thermally evaporated in a high vacuum chamber. As previously demonstrated by Naaman et al, a layer of 10 nm of Au is thin enough to maintain the spin polarization from Ni and inject spin polarized electrons inside the semiconductor.^[^ ^28^, ^29^ ^]^ The two electrodes are polarized by an external magnetic field applied through external magnets: the magnetic field can be parallel (**Figure** **4**a) or antiparallel (Figure 4b) with respect to the direction of the current and the magnetization of the two electrodes is collinear. Since DNTT is a hole carrier material defined as p‐type semiconductor, we refer here to the current generated by the holes transported through the material from the source to the drain. The source acts as a spin polarizer to inject spin‐polarized charges in the chiral semiconductor, then by applying a bias between source and drain the charges are propagated through the material and finally are detected by the drain. The applied magnetic field of 0.2 T is generated by two permanent neodymium magnets parallel to each other (Section S8.1, Supporting Information). By switching source and drain it is possible to invert the direction of the current inside the semiconductor with respect to the applied magnetic field and therefore to measure the impact of the two magnetic field directions on the same device.

The results of these measurements are depicted in Figure 4. OFETs made with (*S*)‐DNTT show a current in the transfer curve only when the magnetic field is parallel to the direction of the hole current. An inversion of the magnetic field direction causes a complete switch off of the device. Conversely, (*R*)‐DNTT OFETs show a response in the transfer curve only when the applied magnetic field is antiparallel to the direction of the current in the semiconductor. Therefore, the current is switched on and off depending on the direction of the magnetic field and on the handedness of the molecule, with the two enantiomers behaving in opposite ways. Devices behavior and properties are not ideal and it is possible to record a signal only in saturation regime (*V*_d_ ≥ −4 V), despite the fact that electrodes have been treated with pentafluorobenzenethiol that does not prevent spin injection.

## 3 Concluding Remarks

We successfully synthesized, purified, and characterized two enantiomers of a chiral DNTT‐based semiconductor. Due to the chirality within the molecule, the crystal structure and the packing of DNTT cores are also chiral, inducing an unusual asymmetric transfer integral distribution. Conventional BGBC OFETs with Au electrodes afford decent characteristics, as testified by *µ* ≈ 0.5 cm^2^ V^−1^ s^−1^ and *V*_th_ ≈ −1.5 V. Applying an external magnetic field of 0.2 T parallel or antiparallel to the charge transport direction does not induce changes in the current intensity for (*S*)‐DNTT, demonstrating no measurable variation of electrical resistance due to eMChA. The situation is, however, radically different when ferromagnetic Ni electrodes covered with Au are used in the same transistor architecture. The current is switched on and off as a function of the parallel or antiparallel orientation of the magnetic field. Knowing that the spin diffusion length in organic materials is at best on the order of 800 nm in comparable thienoacene semiconductors, there is no reason to believe that spin polarization is maintained from S to D electrodes separated by a distance of 215 µm.^[^ ^60^ ^]^ The *I*_DS_ difference is attributed to a magnetoresistance at the spin polarized S and D interfaces. This conclusion is reinforced by the fact that DNTT thin films are polycrystalline with grain size of roughly 100 nm. Even with µm‐long spin diffusion length, it is unphysical that charge carriers would keep their spin polarization upon crossing more than 2000 grain boundaries. We conclude that CISS effect occurs exclusively at the interface with the spin polarized electrodes.

## Conflict of Interest

The authors declare no conflict of interest.

## Supporting information

Supporting Information

## Data Availability

The data that support the findings of this study are available in the supplementary material of this article.
