# Supplementary material for: Enantiopure Dinaphtho[2,3‐b:2,3‐f]thieno[3,2‐b]thiophenes: Reaching High Magnetoresistance Effect in OFETs
Source: Adv Sci (Weinh). 2023 Jul 9;10(26):2301914. doi: 10.1002/advs.202301914 (PMC10502826; doi:10.1002/advs.202301914)
Supplement: Supplementary file 1 — Supporting Information [file ADVS-10-2301914-s001.pdf]

ADVANCED SCIENCE
Open Access

Supporting Information

for *Adv. Sci.*, DOI 10.1002/advs.202301914

Enantiopure Dinaphtho[2,3-*b*:2,3-*f*]thieno[3,2-*b*]thiophenes: Reaching High Magnetoresistance Effect in OFETs

Martina Volpi, Rémy Jouclas, Jie Liu, Guangfeng Liu, Luca Catalano, Nemo McIntosh, Marco Bardini, Christos Gatsios, Federico Modesti, Nicholas Turetta, David Beljonne, Jérôme Cornil, Alan R. Kennedy, Norbert Koch, Peter Erk, Paolo Samorì, Guillaume Schweicher and Yves H. Geerts**

Supporting Information

Enantiopure Dinaphtho[2,3-b:2,3-f]thieno[3,2-b]thiophenes: Reaching High Magnetoresistance Effect in OFETs

Martina Volpi, Rémy Jouclas, Jie Liu, Guangfeng Liu, Luca Catalano, Nemo McIntosh, Marco Bardini, Christos Gatsios, Federico Modesti, Nicholas Turetta, David Beljonne, Jérôme Cornil, Alan R. Kennedy, Norbert Koch, Peter Erk, Paolo Samorì, Guillaume Schweicher, and Yves H. Geerts**

Rémy Jouclas, Jie Liu, Martina Volpi, Guangfeng Liu, Luca Catalano, Guillaume Schweicher, Yves H. Geerts:

Laboratoire de Chimie des Polymères, Faculté des Sciences, Université Libre de Bruxelles (ULB), Boulevard du Triomphe, CP 206/01, 1050 Bruxelles, Belgium
E-mail: guillaume.schweicher@ulb.be, yves.geerts@ulb.be

Nemo McIntosh, Marco Bardini, David Beljonne, Jérôme Cornil
Laboratory for Chemistry of Novel Materials, Center for Research in Molecular Electronics and Photonics, University of Mons, Place du Parc 23, B-7000 Mons, Belgium

Christos Gatsios, Norbert Koch
Helmholtz-Zentrum Berlin für Materialien und Energie GmbH, 12489 Berlin, Germany
Institut für Physik & IRIS Adlershof, Humboldt-Universitat zu Berlin, 12489 Berlin, Germany

Federico Modesti, Peter Erk
BASF SE, RGD - J542S, 67056 Ludwigshafen am Rhein, Germany

Nicholas Turetta, Paolo Samorì
University of Strasbourg, CNRS, ISIS UMR 7006, 8 Alleé Gaspard Monge, F-67000 Strasbourg, France

Alan R. Kennedy
University of Strathclyde, Dept. of Pure and Applied Chemistry, Cathedral Street 295, G1 1XL Glasgow, UK

Yves H. Geerts
International Solvay Institutes for Physics and Chemistry, Université Libre de Bruxelles (ULB), Boulevard du Triomphe, CP 231, 1050 Bruxelles, Belgium

Keywords: CISS effect, chirality, spin, organic semiconductors, transistors, magnetoresistance

TABLE OF CONTENTS 2

1. SYNTHESIS 3

1.1 Experimental procedures 3
1.2 ^{1}H-NMR and ^{13}C-NMR 9
1.3 MS spectra 17
1.4 Experimental UV-Vis absorption spectra 24
1.5 Enantioenrichment by recrystallization 24
1.6 Preparation of the racemic mixture *(R+S)*-DNTT 27

2. UPS AND XPS MEASUREMENTS 27

2.1 XPS Measurements and elemental analysis 28
2.2 Determination of ionization energies from UPS 31

3. DETERMINATION OF IONIZATION ENERGY FROM PYS 32

4. THERMAL CHARACTERIZATION 33

4.1 TGA 33
4.2 DSC 33
4.3 Hot-Stage Optical Microscopy 37
4.4 Variable temperature XRD 37

5. CRYSTAL STRUCTURE 38

5.1 SC-XRD data 38
5.2 Hirshfeld surfaces analysis and fingerprints plot 39
5.3 Powder XRD 41

6. QUANTUM CALCULATIONS 43

6.1 HOMO/LUMO 43

7. ORGANIC FIELD-EFFECT TRANSISTORS WITH AU CONTACTS 44

7.1 Device fabrication 44
7.2 Thin films characterization 46

8. ORGANIC FIELD-EFFECT TRANSISTORS WITH FERROMAGNETIC CONTACTS 48

8.1 Device fabrication 48

9. STRUCTURES OF MOLECULES MENTIONED IN THE TEXT 49

10. REFERENCES 49

1. **Synthesis**

1.1 Experimental procedures

General

All reagents were purchased Sigma-Aldrich (now Merck), VWR, Acros, Alfa Aesar, abcr, TCI and Fluorochem and were used without further purification. Technical grade solvents were purchased from Chem-Lab and used as supplied. Anhydrous solvents as chloroform, dichloromethane, N,Ndimethylformamide and tetrahydrofurane were distilled using common methods. Air- and/or moisture-sensitive liquids and solutions were transferred via a syringe or a Teflon cannula. Analytical thin-layer chromatography (TLC) was performed on aluminum plates with 10-12 µm silica gel containing a fluorescent indicator (Merck silica gel 60 F254). TLC plates were visualized by exposure to ultraviolet light (254 nm and 365 nm). Flash column chromatography was performed on Grace Davisil LC60A (70-200µm) silica. All NMR spectra were recorded on Jeol 400 MHz spectrometer. Chemical shifts are reported in parts per million (ppm, δ scale) from tetramethylsilane for 1H NMR (δ 0 ppm in chloroform and 1,1,2,2-tetrachoroethane) and from the solvent carbon for 13C NMR (e.g., δ 77.16 ppm for chloroform). The data are presented in the following format: chemical shift, multiplicity (s = singlet, d = doublet, t = triplet, m = multiplet), coupling constant in hertz (Hz), signal area integration in natural numbers, assignment (italic). The Mass Spectrometry analyses have been performed in the Organic Synthesis & Mass Spectrometry Laboratory at the University of Mons (Prof. Pascal Gerbaux) using MALDI-MS on a Q-TOF Premier mass spectrometer (Waters, Manchester, UK) in the positive ion mode.

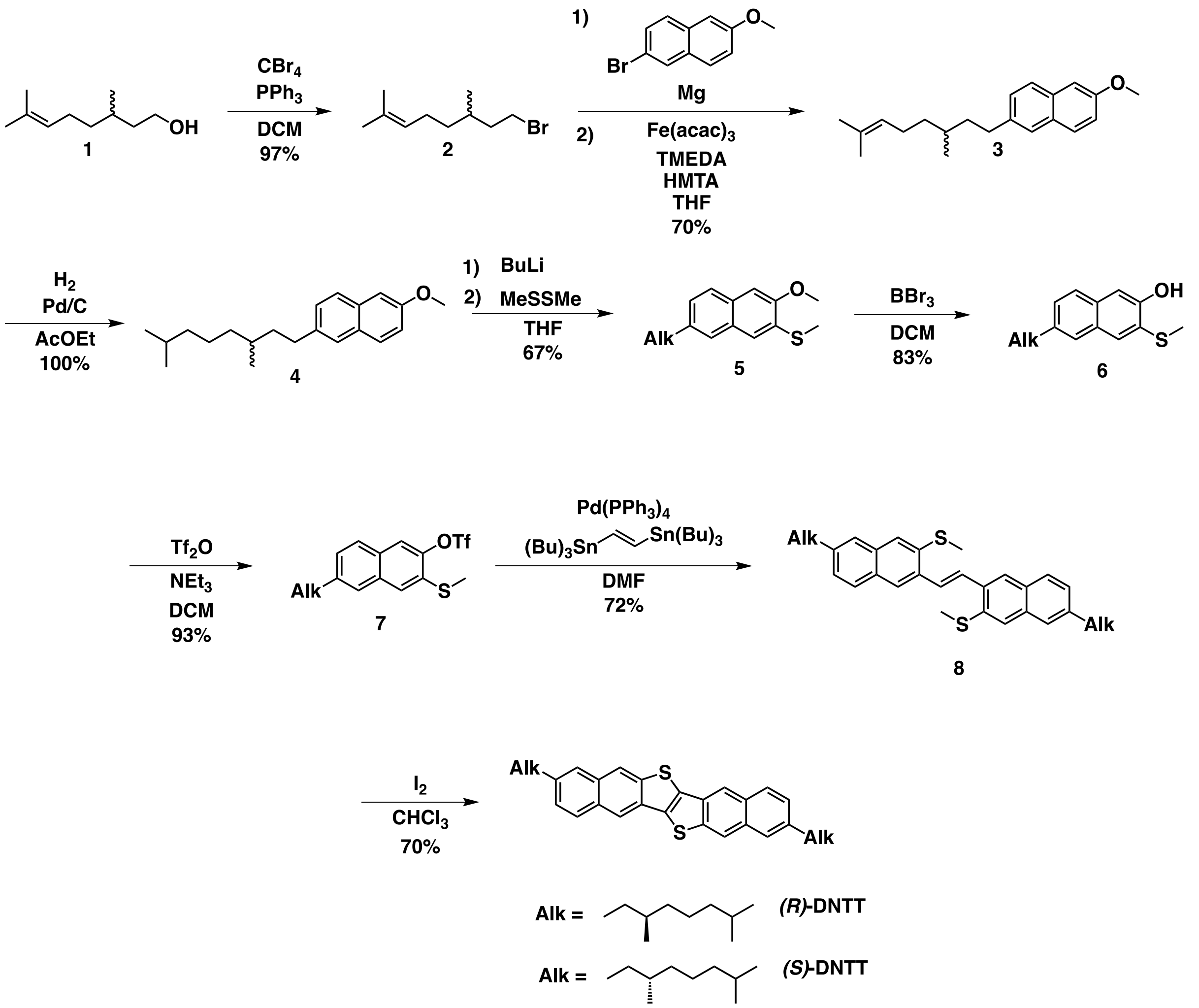

Figure S1- Synthetic scheme for (R)-DNTT and (S)-DNTT

(R)- or (S)- 8-bromo-2,6-dimethyloct-2-ene (2R, 2S)

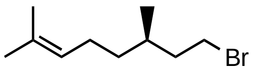

2R

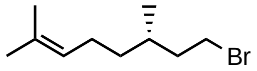

2S

Chemical Formula: $C_{10}H_{19}Br$
Exact Mass: 218,07
Molecular Weight: 219,17

Compound **2R** has been purchased from Merck with an ee = 95%.

Compound **2S** has been synthesized from *(S)*-3,7-dimethyl-6-octen-1-ol (ee = 99%) according to a known procedure.[1]

(S)- or (R)- 2-(3,7-dimethyloct-6-en-1-yl)-6-methoxynaphthalene (3R, 3S)

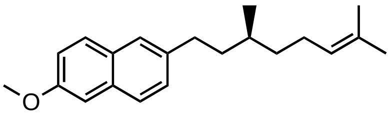

3R

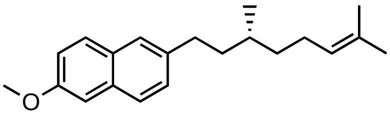

3S

Chemical Formula: $C_{21}H_{28}O$
Exact Mass: 296,21
Molecular Weight: 296,45

A suspension of Mg (4.44g, 182.52 mmol, 4.0 eq) in 30 mL of THF and a catalytic amount of 1,2-dibromoethane was prepared. When the Mg was activated and the reaction started heating, a solution of 2-bromo-6-methoxynaphtalene (21.64 g, 91.6 mmol, 2.0 eq) in 65 mL of THF was added over h under vigorous stirring. After complete addiction, the reaction mixture was cooled to RT. The solution was added dropwise to a second solution of $Fe(acac)_3$ (806 mg, 2.28 mmol, 5 mol%), TMEDA (530 mg, 4.56 mmol, 10 mol%), HMTA (320 mg, 2.28 mmol, 5 mol%) and **2R** (10 g, 45.63 mmol, 1 eq) in 20 mL of THF at 0 °C. The reaction was stirred at room temperature for 2h, quenched in 100 mL of 1M HCl aqueous solution and extracted with 50 mL of EtOAc. The organic phase was dried on $MgSO_4$, filtered and solvents were evaporated. The crude product was purified by silica gel column chromatography (eluent n-heptane/EtOAc 9/1) to give **3R** as a colorless oil (9.5 g, 31.94 mmol) with 70% yield.

^{1}H NMR (400 MHz, $CDCl_3$) δ 7.70 (dd, *J* = 8.4, 2.9 Hz, 2H), 7.57 (s, 1H), 7.33 (dd, *J* = 8.4, 1.8 Hz, 1H), 7.17 – 7.14 (m, 2H), 5.19 – 5.11 (m, 1H), 3.94 (s, 3H), 2.88 – 2.64 (m, 2H), 2.11 – 1.97 (m, 2H), 1.73 (s, 3H), 1.65 (s, 3H), 1.59 – 1.39 (m, 3H), 1.26 (dddd, *J* = 13.3, 9.5, 7.2, 5.9 Hz, 1H), 1.01 (d, *J* = 6.2 Hz, 3H).

^{13}C NMR (101 MHz, $CDCl_3$) δ 157.19, 138.44, 132.99, 131.23, 129.28, 128.98, 128.03, 126.76, 126.19, 125.26, 125.06, 118.69, 105.78, 55.37, 38.99, 37.29, 37.12, 33.51, 32.54, 32.28, 25.84, 25.72, 25.64, 19.70, 17.76.

HRMS (MALDI) *m/z*: [M]+ calcd for $C_{21}H_{28}O$, 296.2140; found, 296.2151

TLC (Heptane/EtOAc 9:1) : Rf = 0.73

The same procedure was applied to synthesize **3S**.

(S)- or (R)- 2-(3,7-dimethyloctyl)-6-methoxynaphthalene (4R, 4S)

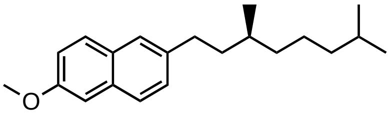

4R

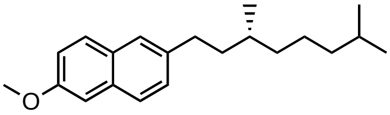

4S

Chemical Formula: $C_{21}H_{30}O$
Exact Mass: 298,23
Molecular Weight: 298,47

To a solution of **3R** (8.103g, 27.33 mmol, 1 eq) in 260 mL of EtOAc, Pd/C (10 wt.%, 1 g) was added and the reaction was stirred overnight in H_2 atmosphere (balloon pressure). The reaction mixture was filtered on a silica pad eluting with n-heptane. The product **4R** (8.15 g, 27.33 mmol) was collected in quantitative yield as a pure transparent oil crystallizing at room temperature.

^{1}H NMR (400 MHz, $CDCl_3$) δ 7.68 (dd, *J* = 9.1, 3.0 Hz, 2H), 7.56 (s, 1H), 7.32 (dd, *J* = 8.4, 1.8 Hz, 1H), 7.18 – 7.10 (m, 2H), 3.92 (s, 3H), 2.85 – 2.65 (m, 2H), 1.72 (dddd, *J* = 14.7, 12.4, 6.6, 4.0 Hz, 1H), 1.56 – 1.09 (m, 9H), 0.97 (d, *J* = 6.3 Hz, 3H), 0.89 (d, *J* = 6.6 Hz, 6H).

^{13}C NMR (101 MHz, $CDCl_3$) δ 157.16, 138.49, 132.96, 129.26, 128.96, 128.02, 126.73, 126.16, 118.66, 105.77, 77.43, 77.12, 76.80, 55.37, 39.44, 39.05, 37.29, 33.54, 32.62, 28.07, 24.81, 22.81, 22.72, 19.76.

HRMS (MALDI) *m/z*: [M]+ calcd for $C_{21}H_{30}O$, 298.2297; found, 298.2309

TLC (Heptane/AcOEt 9.8:0.2) : Rf = 0.62

The same procedure was applied to synthesize **4S**.

(S)- or (R)- (7-(3,7-dimethyloctyl)-3-methoxynaphthalen-2-yl)(methyl)sulfane (5R, 5S)

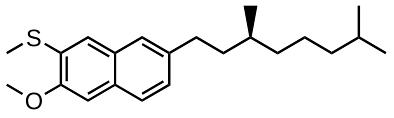

5R

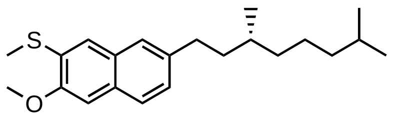

5S

Chemical Formula: $C_{22}H_{32}OS$
Exact Mass: 344,22
Molecular Weight: 344,56

To a solution of **4R** (7.16 g, 23.99 mmol, 1 eq) in 30 mL of THF, was added BuLi (2.5 M in exane, 26.39 mmol, 1.1 eq) at -78 °C. The reaction was stirred at room temperature for 1h30. MeSSMe, previously dried on molecular sieves, (2.71 g, 28.79 mmol, 1.2 eq) was added at -78 °C and the reaction was stirred at room temperature overnight. The reaction mixture was quenched in 60 mL of a solution of NH_4Cl in water (10% w/w) and extracted with Et_2O (3 x 30 mL). The organic phase was washed with brine, dried over $MgSO_4$, filtered and the solvents were evaporated. The crude product was purified by silica gel column chromatography (eluent heptane/EtOAc 9.4/0.6) to give **5R** (5.31 g, 16.07 mmol) as a pale yellow oil with 67% yield.
^{1}H NMR (400 MHz, $CDCl_3$) δ 7.61 (d, *J* = 8.6 Hz, 1H), 7.48 (s, 1H), 7.40 (s, 1H), 7.23 (dd, *J* = 8.3, 1.7 Hz, 1H), 7.04 (s, 1H), 3.99 (s, 3H), 2.82 – 2.61 (m, 2H), 2.52 (s, 3H), 1.75 – 1.62 (m, 1H), 1.57 – 1.43 (m, 2H), 1.37 – 1.08 (m, 7H), 0.94 (d, *J* = 6.3 Hz, 3H), 0.86 (d, *J* = 6.6 Hz, 6H).
^{13}C NMR (101 MHz, $CDCl_3$) δ 154.20, 139.11, 130.56, 129.62, 129.55, 127.12, 126.48, 125.07, 123.01, 104.80, 56.00, 39.48, 39.07, 37.33, 33.63, 32.64, 28.11, 24.84, 22.85, 22.76, 19.81, 14.73.
HRMS (MALDI) *m/z*: [M]+ calcd for $C_{22}H_{32}OS$, 344.2174; found, 344.2187
TLC (Heptane/AcOEt 9.4:0.6) : Rf = 0.56

The same procedure was applied to synthesize **5S**.

(S)- or (R)- 6-(3,7-dimethyloctyl)-3-(methylthio)naphthalen-2-ol (6R, 6S)

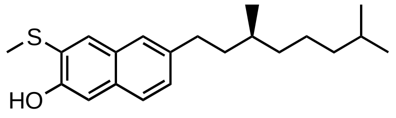

6R

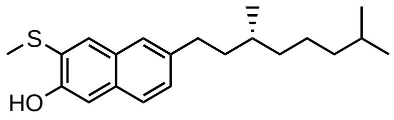

6S

Chemical Formula: $C_{21}H_{30}OS$
Exact Mass: 330,20
Molecular Weight: 330,53

To a solution of **5R** (5.989 g, 17.38 mmol, 1 eq) in 90 mL of DCM dry, was added BBr_3 (1M solution in DCM, 34.76 mmol, 2 eq) dropwise at 0 °C and the reaction was stirred at room temperature overnight. The reaction was quenched in ice and extracted with DCM (3 x 40 mL). The organic phase was dried on $MgSO_4$ and the solvents were evaporated. The crude product was purified by silica gel column chromatography (eluent: heptane/EtOAc 6.4/0.6) to obtain **6R** (4.77 g, 14.42 mmol) as a pale yellow oil with 83% yield.
^{1}H NMR (400 MHz, $CDCl_3$) δ 7.94 (s, 1H), 7.61 (d, J = 8.5 Hz, 1H), 7.49 (s, 1H), 7.33 – 7.28 (m, 2H), 6.56 (s, 1H), 2.88 – 2.60 (m, 2H), 2.42 (s, 3H), 1.76 – 1.62 (m, 1H), 1.60 – 1.09 (m, 9H), 0.96 (d, J = 6.3 Hz, 3H), 0.87 (d, J = 6.6 Hz, 6H).
^{13}C NMR (101 MHz, $CDCl_3$) δ 152.24, 138.85, 133.65, 133.60, 129.33, 128.85, 126.48, 125.71, 124.26, 109.18, 39.47, 38.96, 37.31, 33.56, 32.63, 28.10, 24.83, 22.85, 22.76, 20.01, 19.79.

HRMS (MALDI) *m/z*: [M]+ calcd for $C_{21}H_{30}OS$, 330.2017; found, 330.2028
TLC (Heptane/AcOEt 4:1) : Rf = 0.41

The same procedure was applied to synthesize **6S**.

(S)- or (R) -6-(3,7-dimethyloctyl)-3-(methylthio)naphthalen-2-yl trifluoromethanesulfonate (7R, 7S)

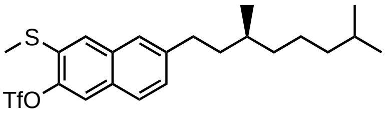

7R

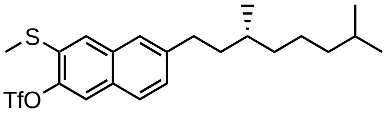

7S

Chemical Formula: $C_{22}H_{29}F_3O_3S_2$
Exact Mass: 462,15
Molecular Weight: 462,59

To a solution of **6R** (4.145g, 12.54 mmol, 1 eq) and Net3 (3.04 g, 30.10 mmol, 2.4 eq) in 60 mL of DCM, triflic anhydride (4.246 g, 15.05 mmol, 1.2 eq) was added dropwise at 0 °C. The reaction mixture was stirred at room temperature for 2h, quenched in 100 mL of water, extracted with DCM (3 x 20 mL) and the organic phase was washed with HCl (1M in water). The organic phase was dried on $MgSO_4$, filtered and the solvents were evaporated. The crude product was purified by silica gel column chromatography (eluent: heptane/Et_2O 9.8/0.2) to obtain **7R** (5.39 g, 11.66 mmol) as a pale yellow oil with 93% yield.
^{1}H NMR (400 MHz, $CDCl_3$) δ 7.72 (d, *J* = 8.4 Hz, 1H), 7.68 (s, 1H), 7.63 (s, 1H), 7.58 (s, 1H), 7.36 (dd, *J* = 8.4, 1.7 Hz, 1H), 2.87 – 2.68 (m, 2H), 2.59 (s, 3H), 1.75 – 1.65 (m, 1H), 1.59 – 1.43 (m, 3H), 1.41 – 1.09 (m, 9H), 0.96 (d, *J* = 6.3 Hz, 3H), 0.87 (d, *J* = 6.6 Hz, 6H).
^{13}C NMR (101 MHz, $CDCl_3$) δ 144.96, 143.06, 133.10, 130.79, 129.56, 128.48, 127.79, 126.49, 125.24, 119.27, 77.42, 77.10, 76.78, 39.39, 38.74, 37.22, 33.73, 32.57, 32.02, 29.80, 28.05, 24.77, 22.78, 19.69, 15.92, 14.19.
HRMS (MALDI) *m/z*: [M]+ calcd for $C_{22}H_{29}F_3O_3S_2$, 462.1510; found, 462.1521
TLC (Heptane/Et_2O 9.8:0.2) : Rf = 0.56

The same procedure was applied to synthesize **7S**.

(E)-1,2-bis(6-((R or S)-3,7-dimethyloctyl)-3-(methylthio)naphthalen-2-yl)ethene (8R, 8S)

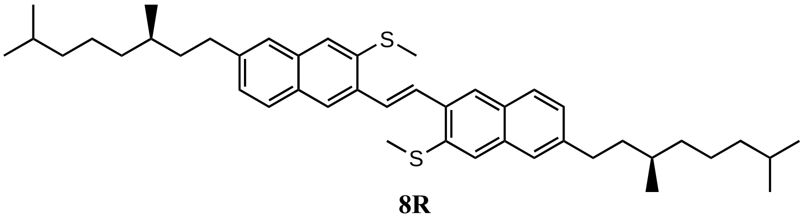

8R

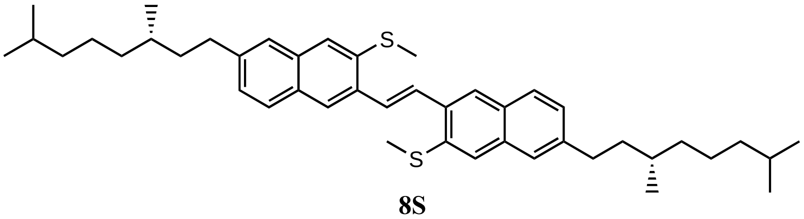

8S

Chemical Formula: $C_{44}H_{60}S_2$
Exact Mass: 652,41
Molecular Weight: 653,08

A solution of **7R** (5.105 g, 11.04 mmol, 2eq) in 80 mL of dry DMF was degassed with 3 freeze-pump-thaw cycles. The DMF solution was added to a second flask containing $Pd(PPh_3)_4$ (383 mg, 0.33 mmol, 6 mol%) in argon atmosphere. Trans-1,2-Bis(tributylstannyl)ethene (3.34 g, 5.52 mmol, 1 eq) was added and the reaction was stirred at

100 °C in dark overnight. The reaction mixture was quenched in a saturated solution of KF in water, extracted with $CHCl_3$ (3 x 40 mL) and washed with brine (3 x 40 mL). The organic phase was dried on $MgSO_4$, filtered and solvents were evaporated. The crude of reaction was filtered on silica eluting with heptane to obtain **8R** (2.59 g, 3.97 mmol) as an orange oil that crystallize at room temperature with 72% yield.
^{1}H NMR (400 MHz, $CDCl_3$) δ 8.05 (s, 2H), 7.76 (d, *J* = 8.4 Hz, 2H), 7.64 (s, 2H), 7.59 (s, 2H), 7.52 (s, 1H), 7.28 (dd, *J* = 8.4, 1.7 Hz, 2H), 2.86 – 2.69 (m, 4H), 2.57 (s, 6H), 1.77 – 1.63 (m, 2H), 1.58 – 1.48 (m, 6H), 1.40 – 1.11 (m, 18H), 0.96 (d, *J* = 6.4 Hz, 6H), 0.87 (d, *J* = 6.7 Hz, 12H).
^{13}C NMR (101 MHz, $CDCl_3$) δ 141.64, 135.85, 134.42, 133.75, 130.20, 128.36, 127.93, 127.42, 125.10, 125.04, 124.16, 77.48, 77.16, 76.84, 39.49, 38.88, 37.33, 35.57, 33.86, 32.67, 32.03, 28.12, 24.86, 22.86, 22.77, 19.81, 16.60.
HRMS (MALDI) *m/z*: [M]+ calcd for $C_{44}H_{60}S_2$, 652.4161; found, 652.4170
TLC (Heptane/AcOEt 9.6:0.4) : Rf = 0.40

The same procedure was applied to synthesize **8S**.

2,9-bis((R or S)-3,7-dimethyloctyl)naphtho[2,3-b]naphtho[2',3':4,5]thieno[2,3-d]thiophene ((R)-DNTT, (S)-DNTT)

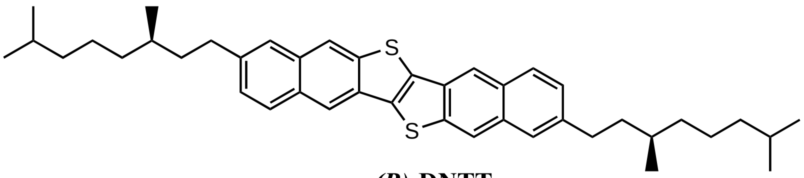

***(R)*-DNTT**

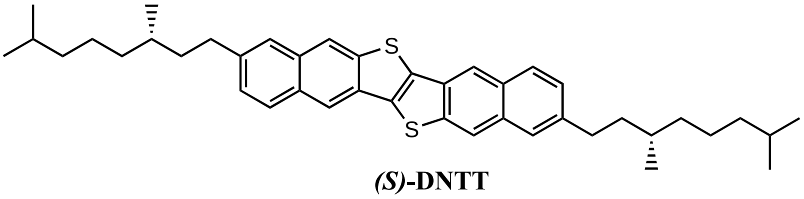

***(S)*-DNTT**

Chemical Formula: $C_{42}H_{52}S_2$
Exact Mass: 620,35
Molecular Weight: 621,00

8R (648 mg, 0.99 mmol, 1 eq) and I_2 (8.04 g, 31.7 mmol, 32 eq) were dissolved in 25 mL of $CHCl_3$ and heated at reflux overnight under argon atmosphere. The solvent was evaporated until only few mL of $CHCl_3$ were left. 20 mL of MeOH were added and the solution was stirred for 2 hours. 40 mL of a saturated solution of $NaSO_3H$ was added and the solution was stirred for 2 hours. The yellow precipitate was filtered washing with Heptane. The final product ***(R)*-DNTT** was collected as a yellow precipitate with 81% yield (501 mg, 0.81 mmol).
^{1}H NMR (400 MHz, $CDCl_3$) δ 8.32 (d, *J* = 8.6 Hz, 4H), 7.95 (d, *J* = 8.9 Hz, 2H), 7.70 (s, 2H), 7.39 (dd, *J* = 8.5, 1.7 Hz, 2H), 2.93 – 2.72 (m, 4H), 1.71-1.81 (m, 2H), 1.42 – 1.10 (m, 16H), 0.99 (d, *J* = 6.3 Hz, 6H), 0.87 (d, *J* = 6.6 Hz, 12H).
HRMS (MALDI) *m/z*: [M]+ calcd for $C_{42}H_{52}S_2$, 620.3534; found, 620.3510.
Mp = 254.8 °C

The same procedure was applied to synthesize ***(S)*-DNTT**.

1.2 ^{1}H-NMR and ^{13}C-NMR

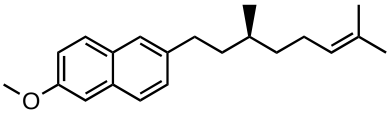

3R

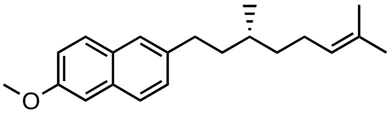

3S

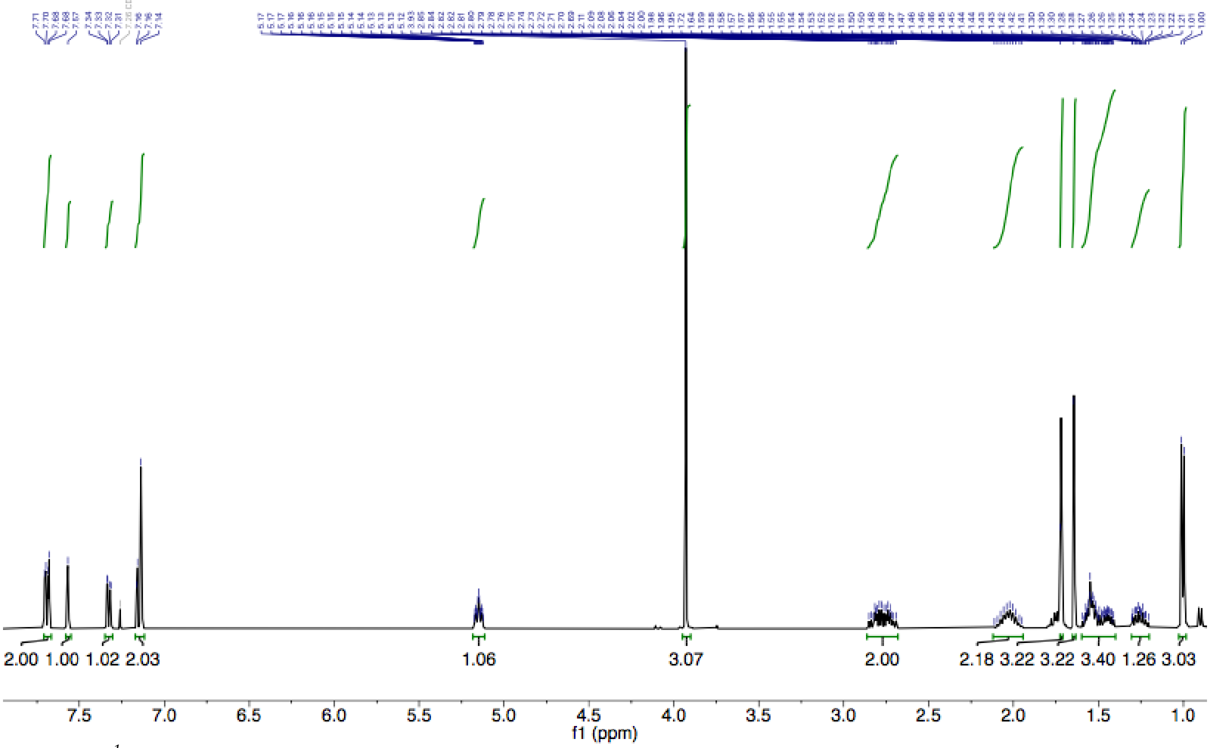

Figure S2 -^{1}H-NMR spectra in $CDCl_3$ of 3R and 3S

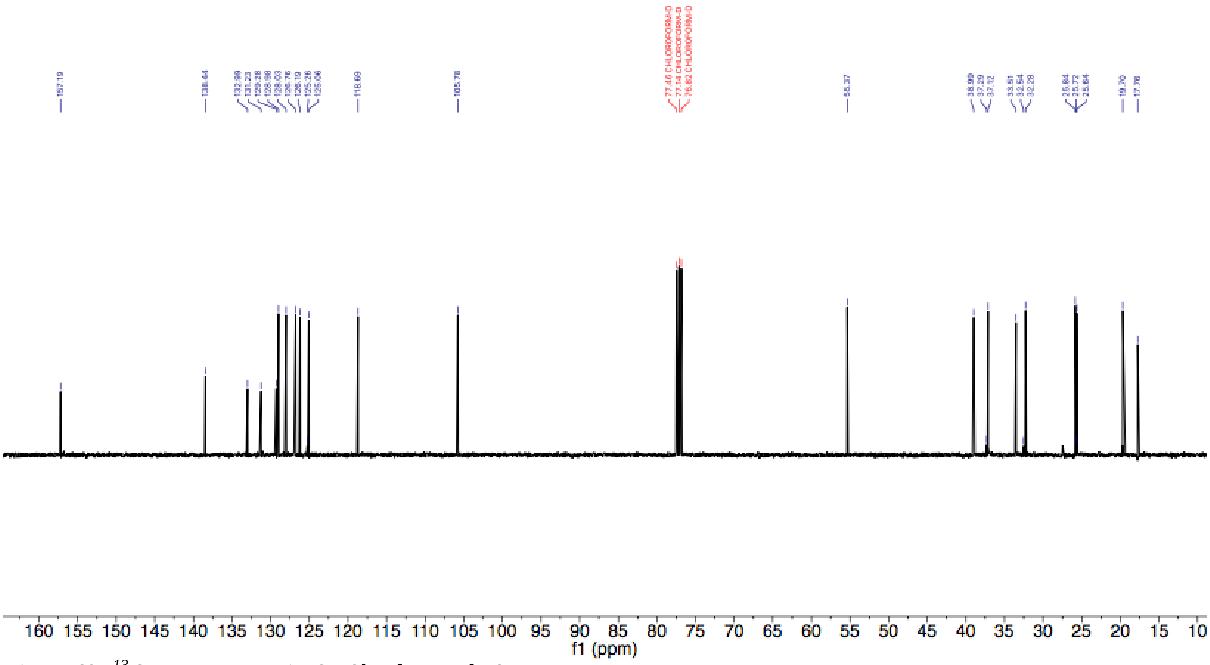

Figure S3 -^{13}C-NMR spectra in $CDCl_3$ of 3R and 3S

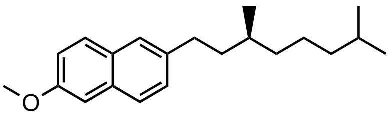

4R

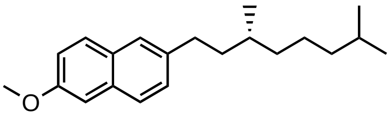

4S

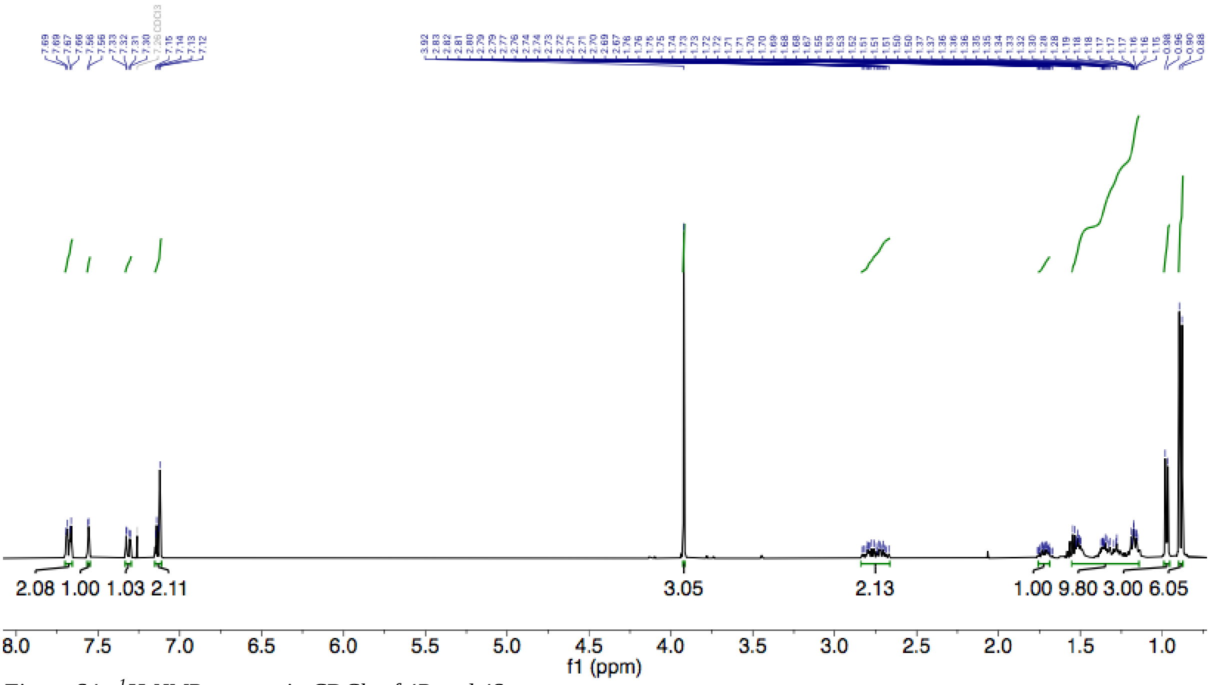

Figure S4 - ^{1}H-NMR spectra in $CDCl_3$ of 4R and 4S

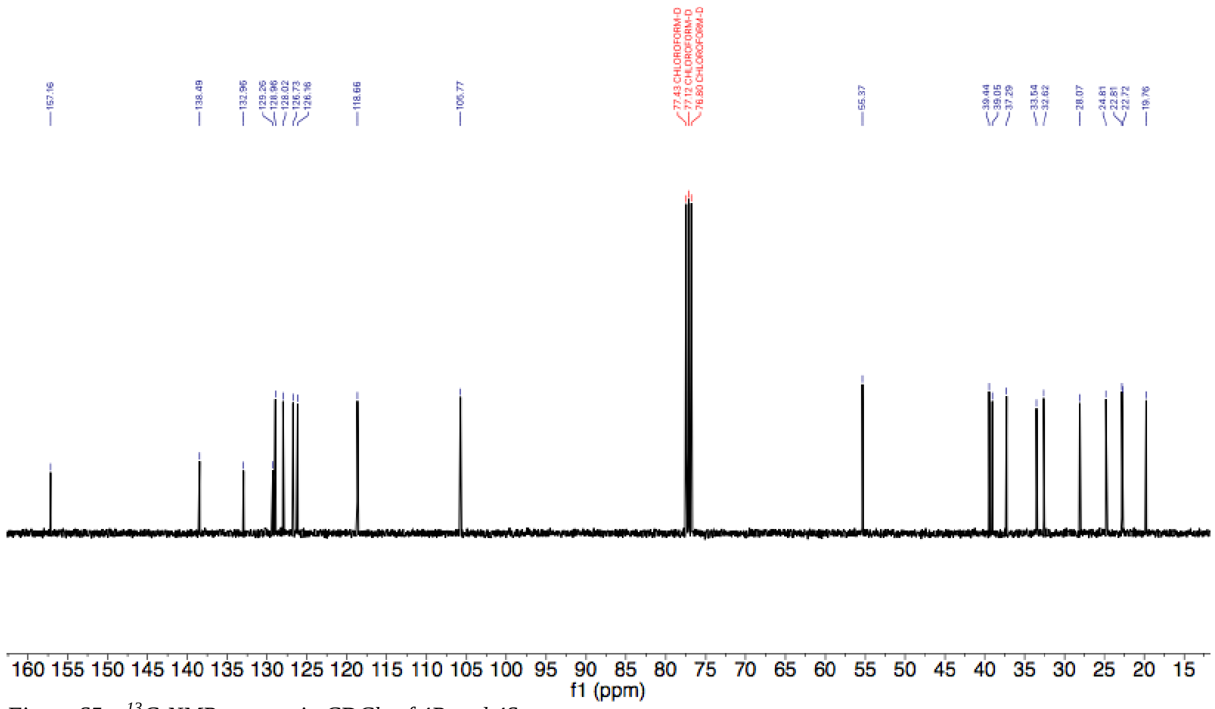

Figure S5 – ^{13}C-NMR spectra in $CDCl_3$ of 4R and 4S

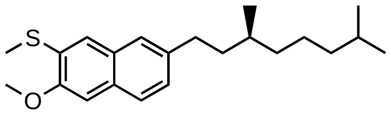

5R

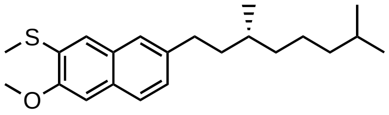

5S

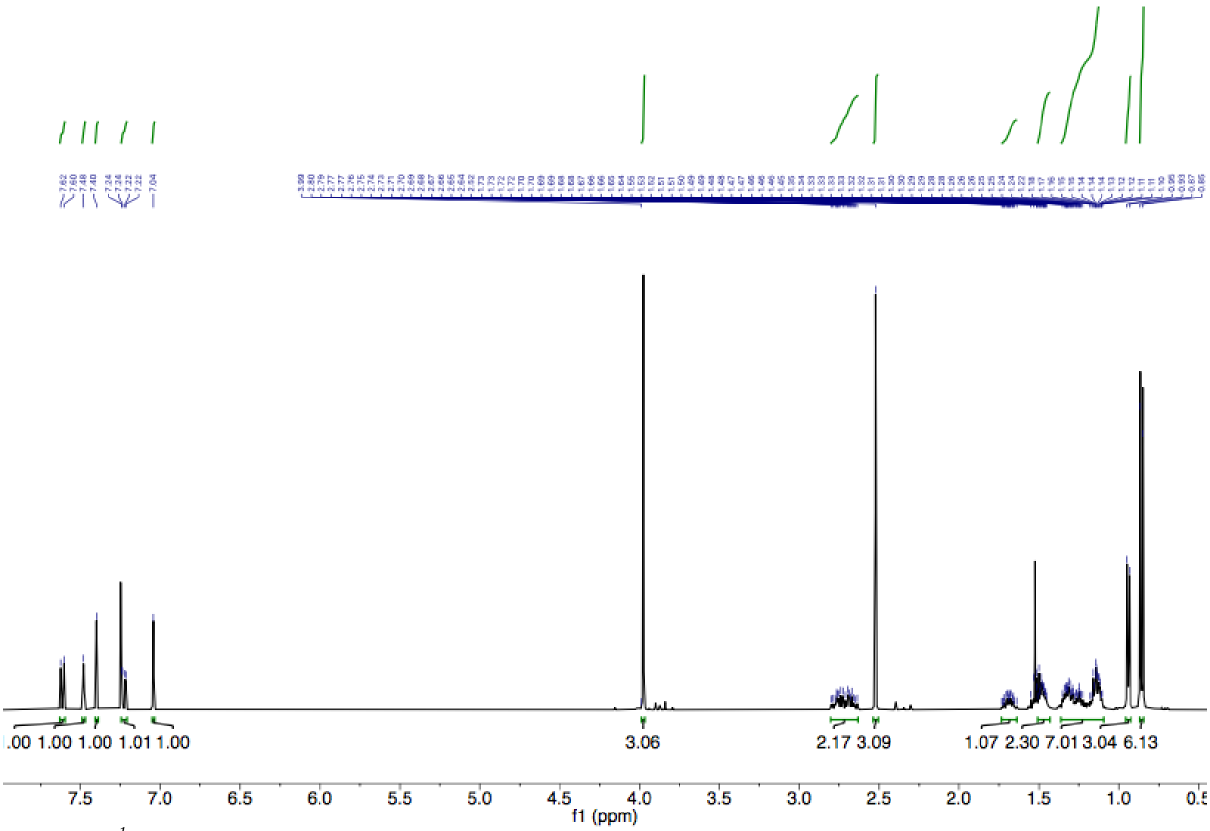

Figure S6 - ^{1}H-NMR spectra in $CDCl_3$ of 5R and 5S

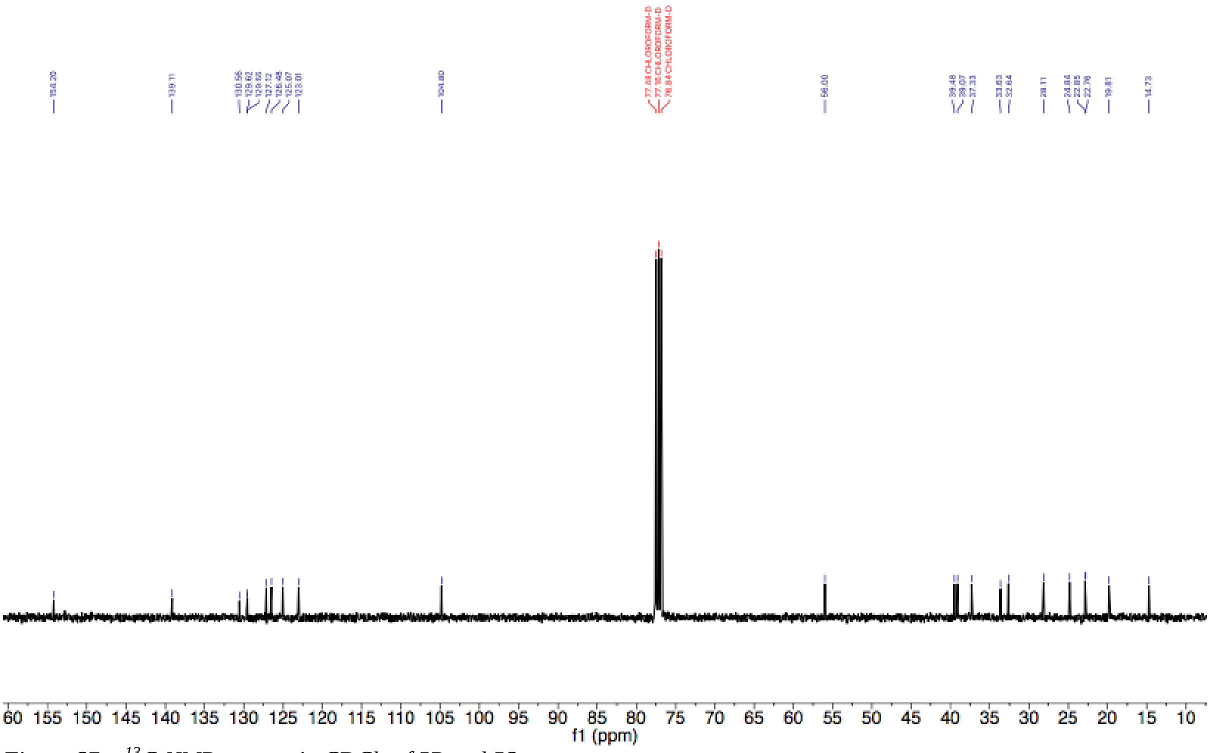

Figure S7 - ^{13}C-NMR spectra in $CDCl_3$ of 5R and 5S

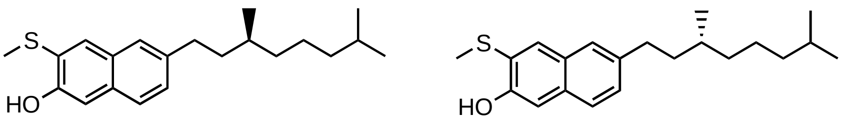

6R **6S**

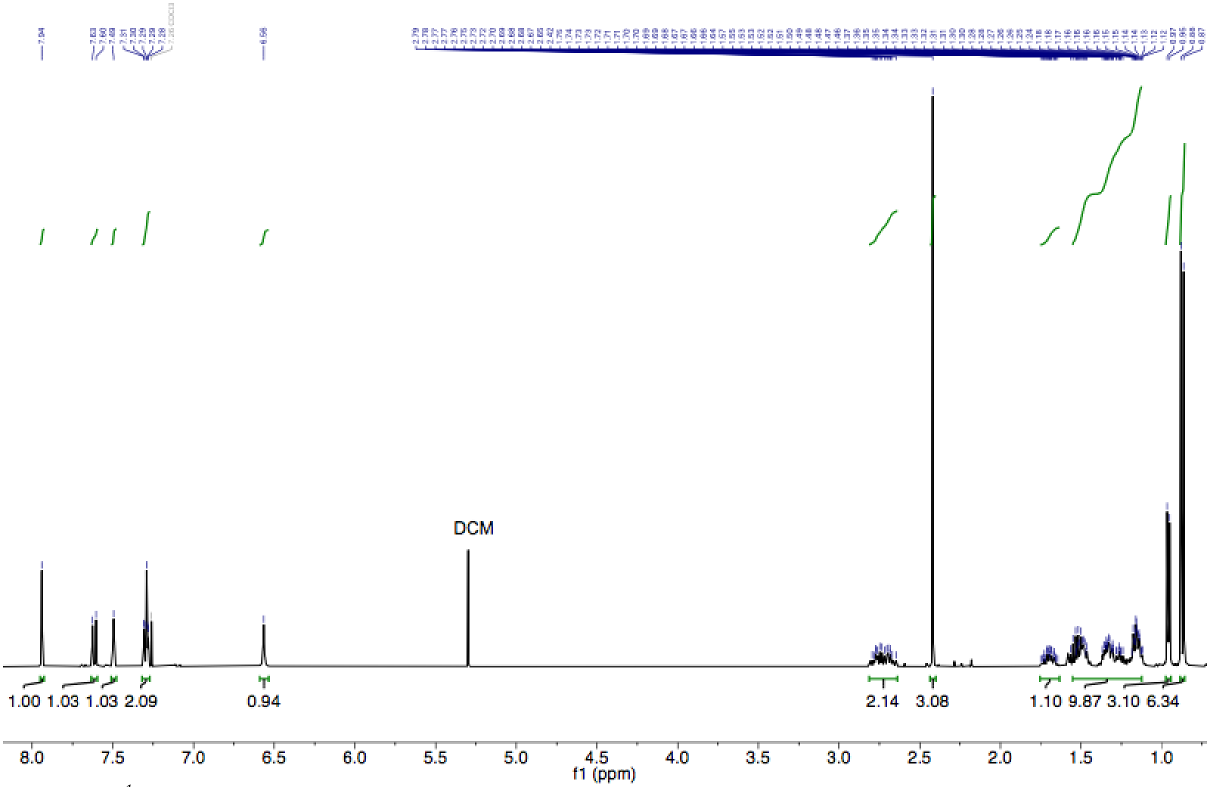

Figure S8 - ^{1}H-NMR spectra in $CDCl_3$ of 6R and 6S

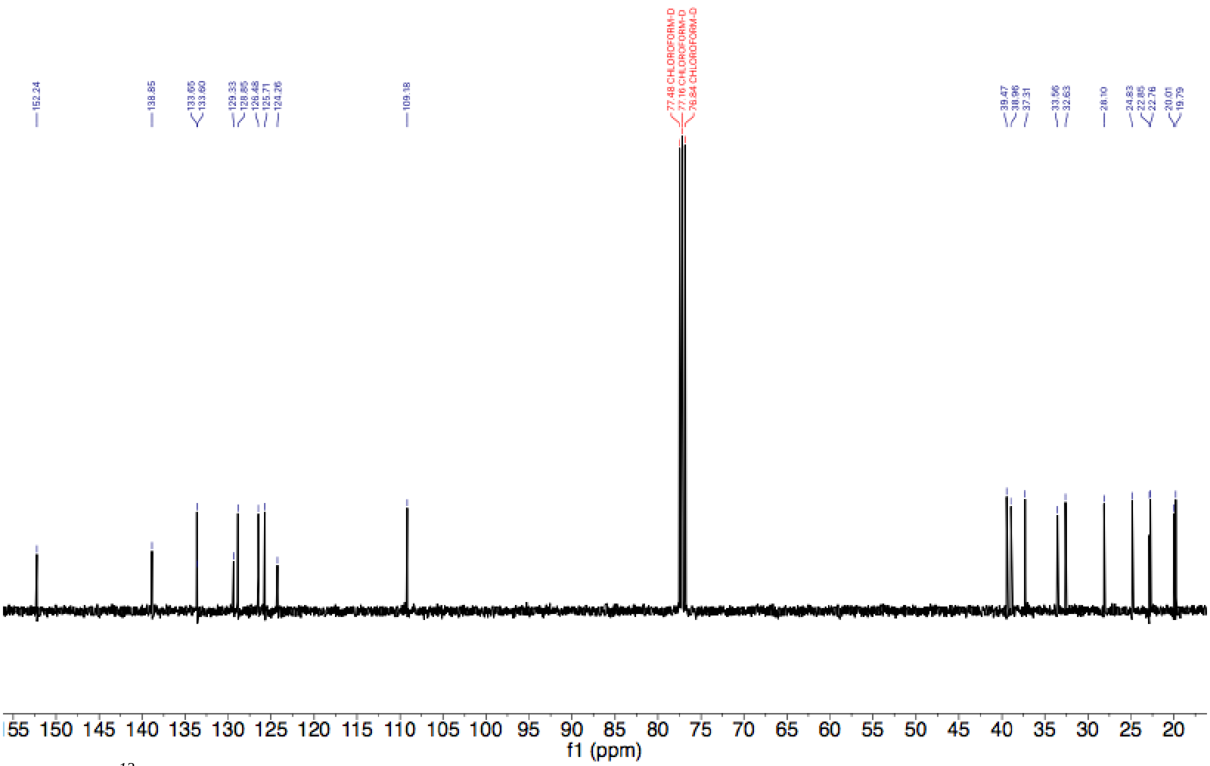

Figure S9 - ^{13}C-NMR spectra in $CDCl_3$ of 6R and 6S

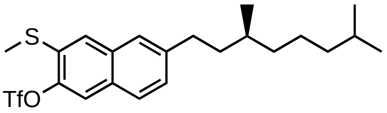

7R

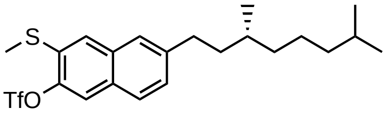

7S

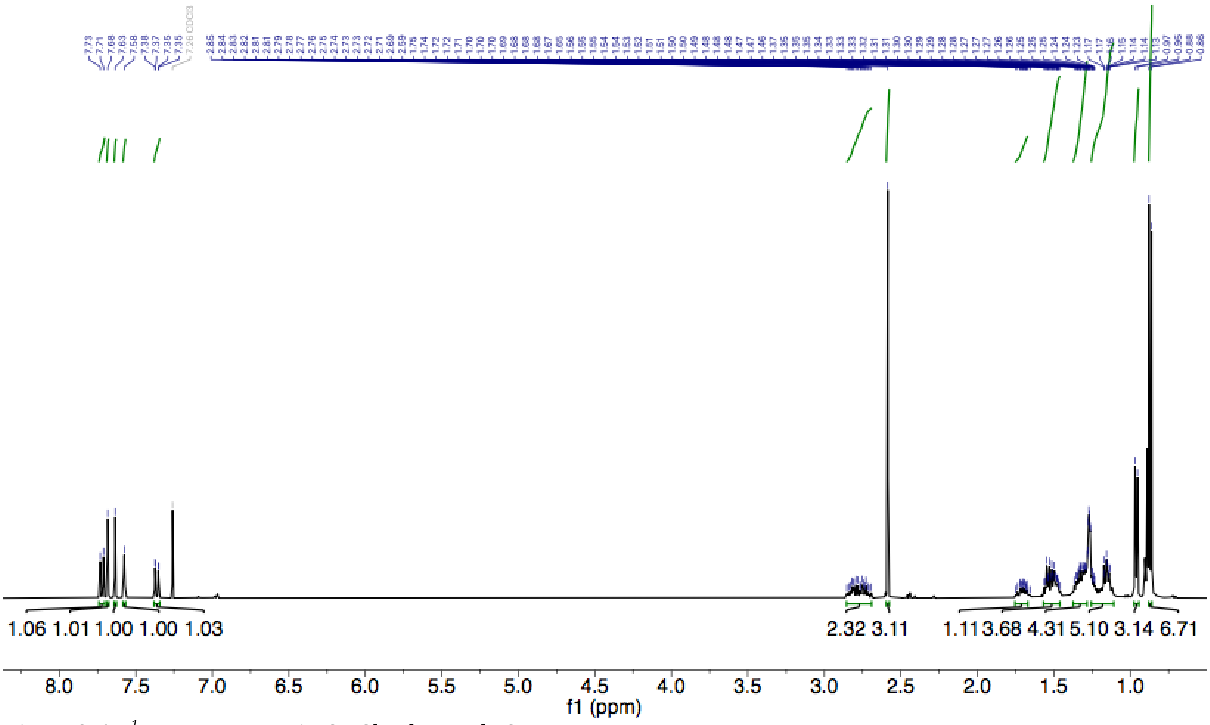

Figure S10 - ^{1}H-NMR spectra in $CDCl_3$ of 7R and 7S

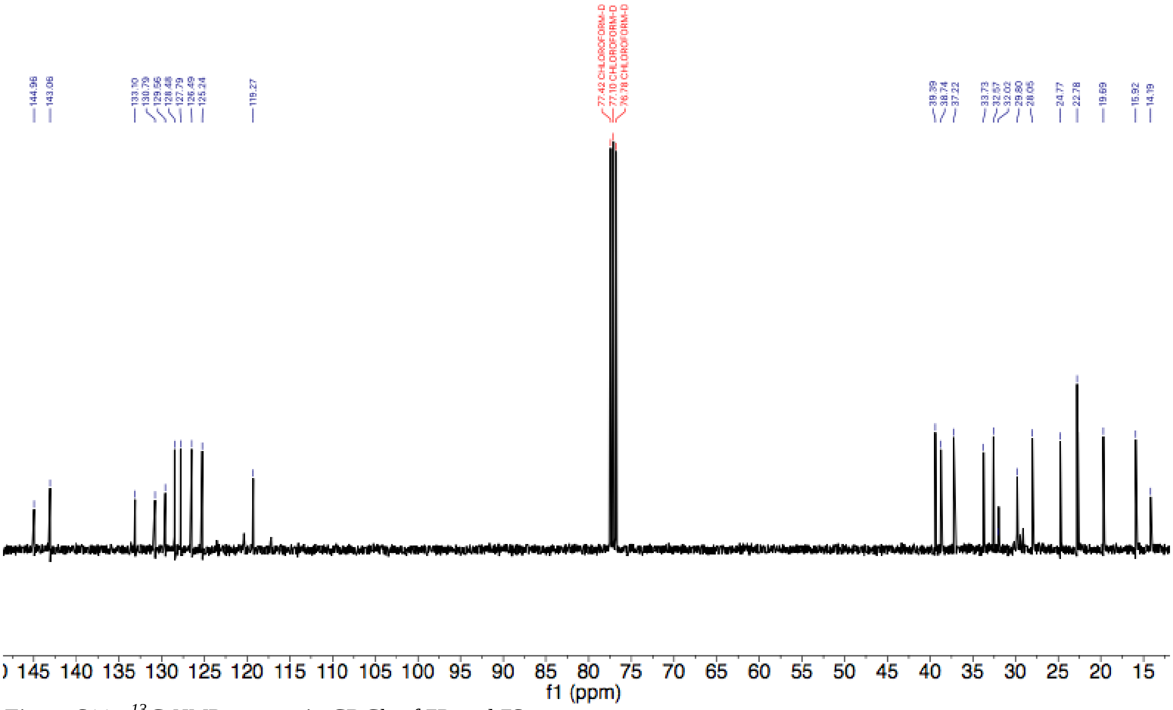

Figure S11 - ^{13}C-NMR spectra in $CDCl_3$ of 7R and 7S

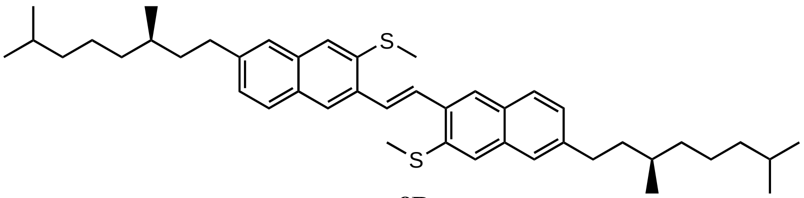

8R

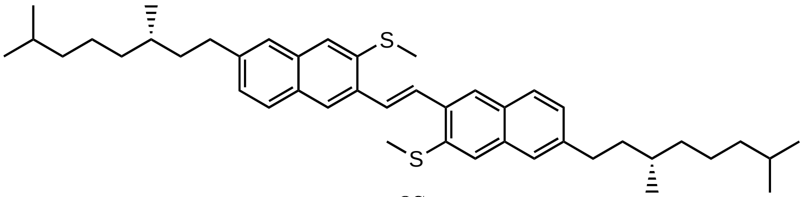

8S

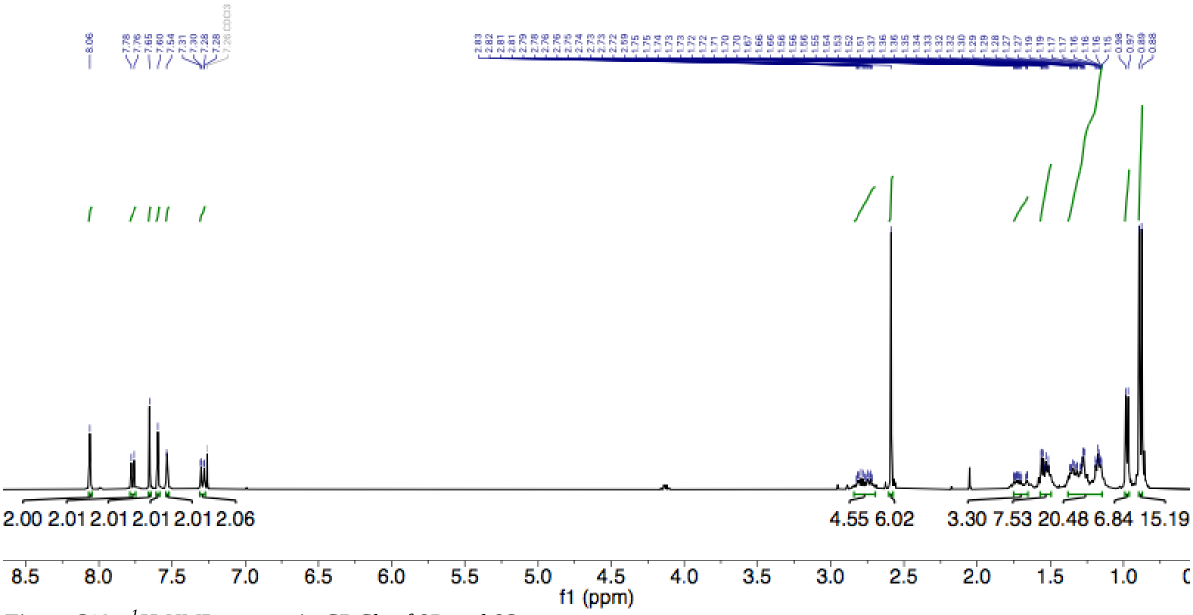

Figure S12 - ^{1}H-NMR spectra in $CDCl_3$ of 8R and 8S

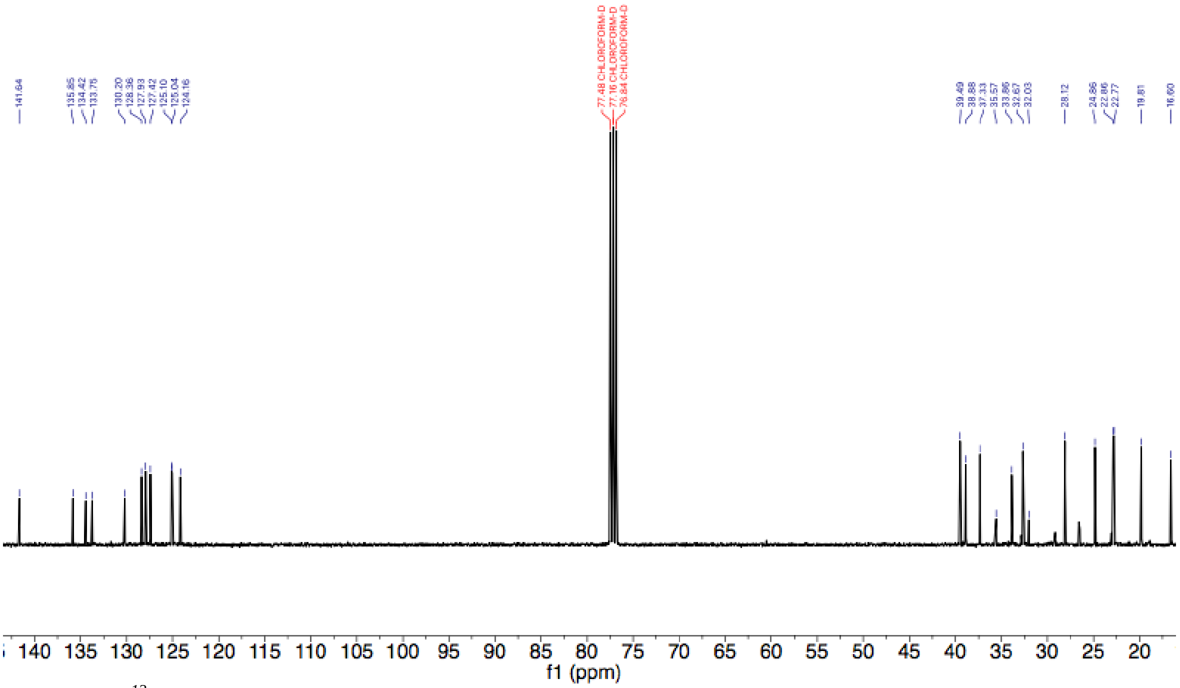

Figure S13 - ^{13}C-NMR spectra in $CDCl_3$ of 8R and 8S

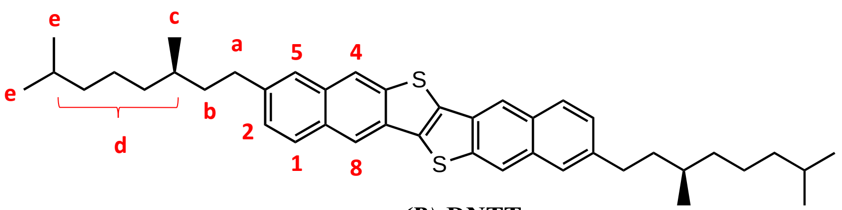

***(R)*-DNTT**

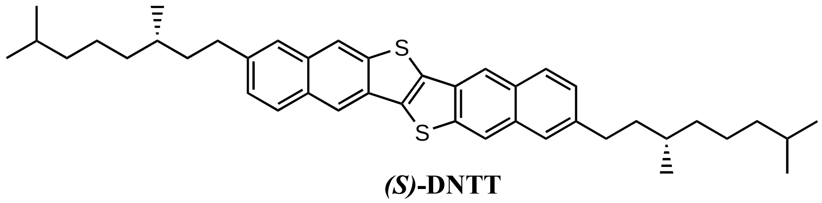

***(S)*-DNTT**

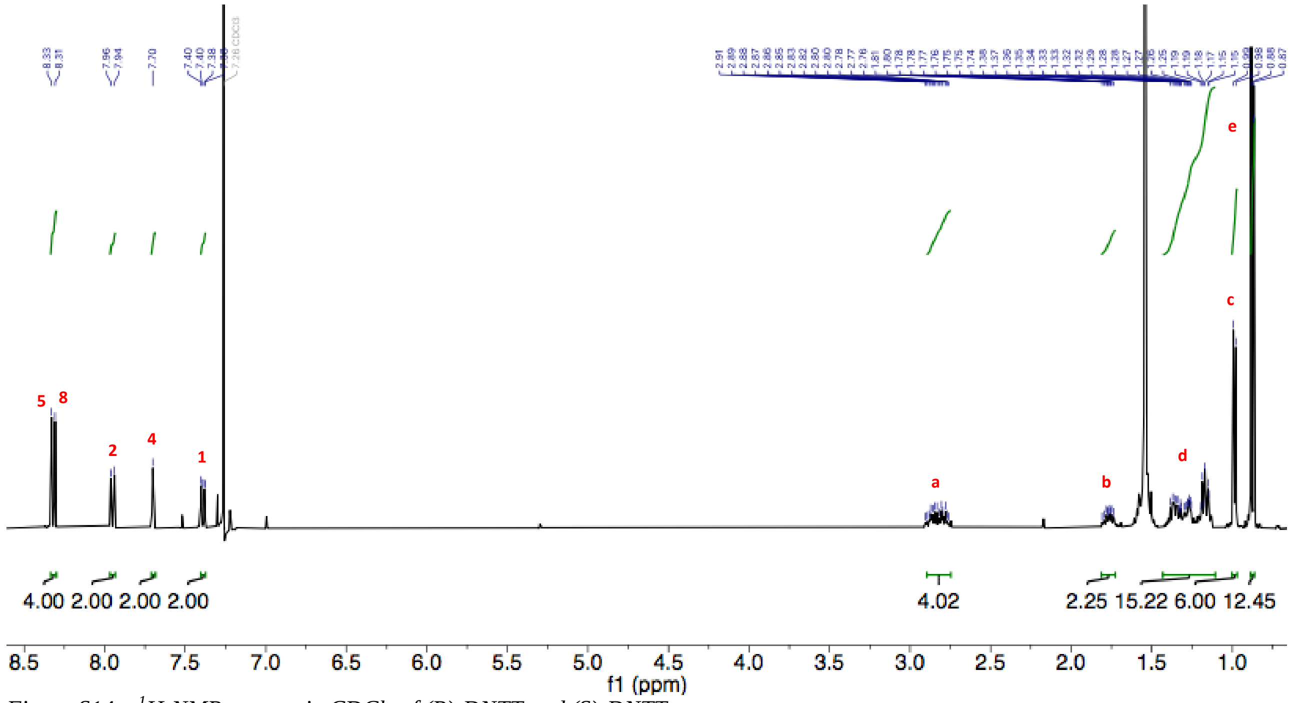

Figure S14 – ^{1}H-NMR spectra in $CDCl_3$ of (R)-DNTT and (S)-DNTT

1.3 MS spectra

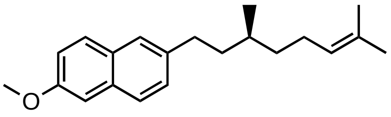

3R

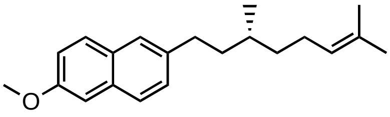

3S

Chemical Formula: $C_{21}H_{28}O$
Exact Mass: 296,21
Molecular Weight: 296,45

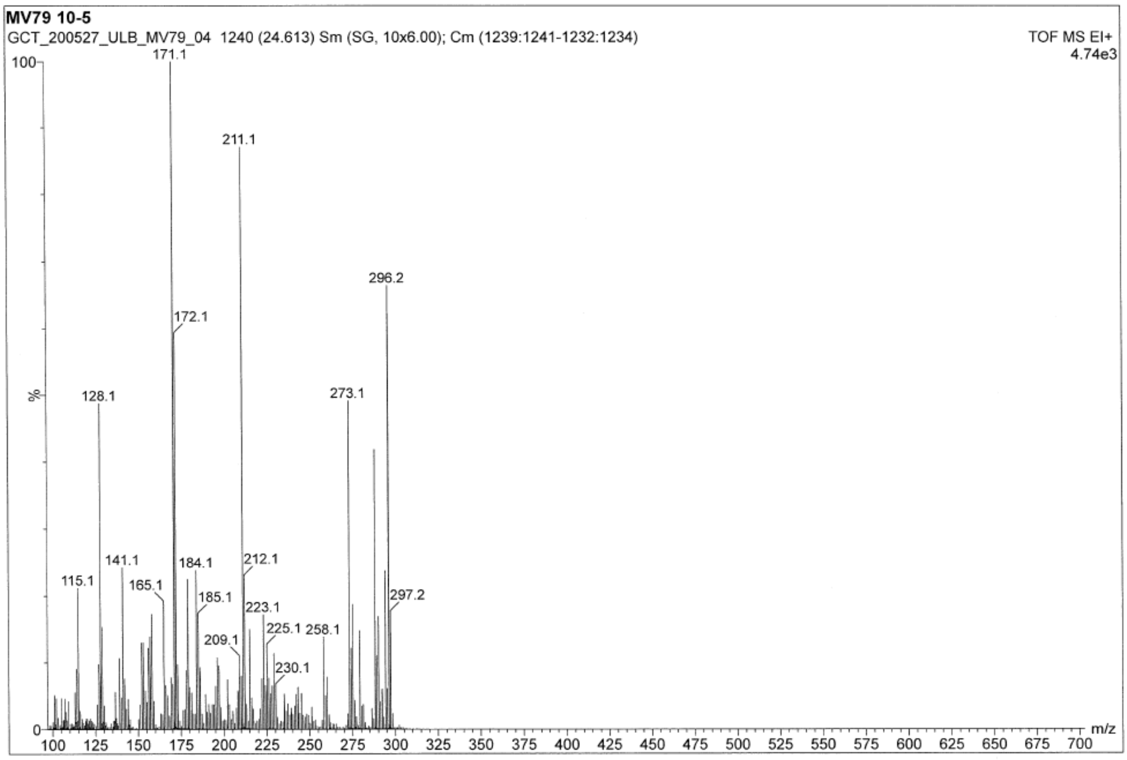

Figure S15 – HRMS spectra of 3R and 3S

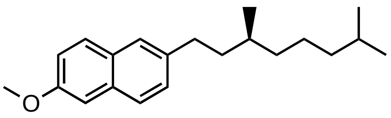

4R

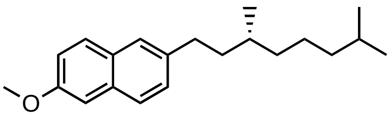

4S

Chemical Formula: $C_{21}H_{30}O$
Exact Mass: 298,23
Molecular Weight: 298,47

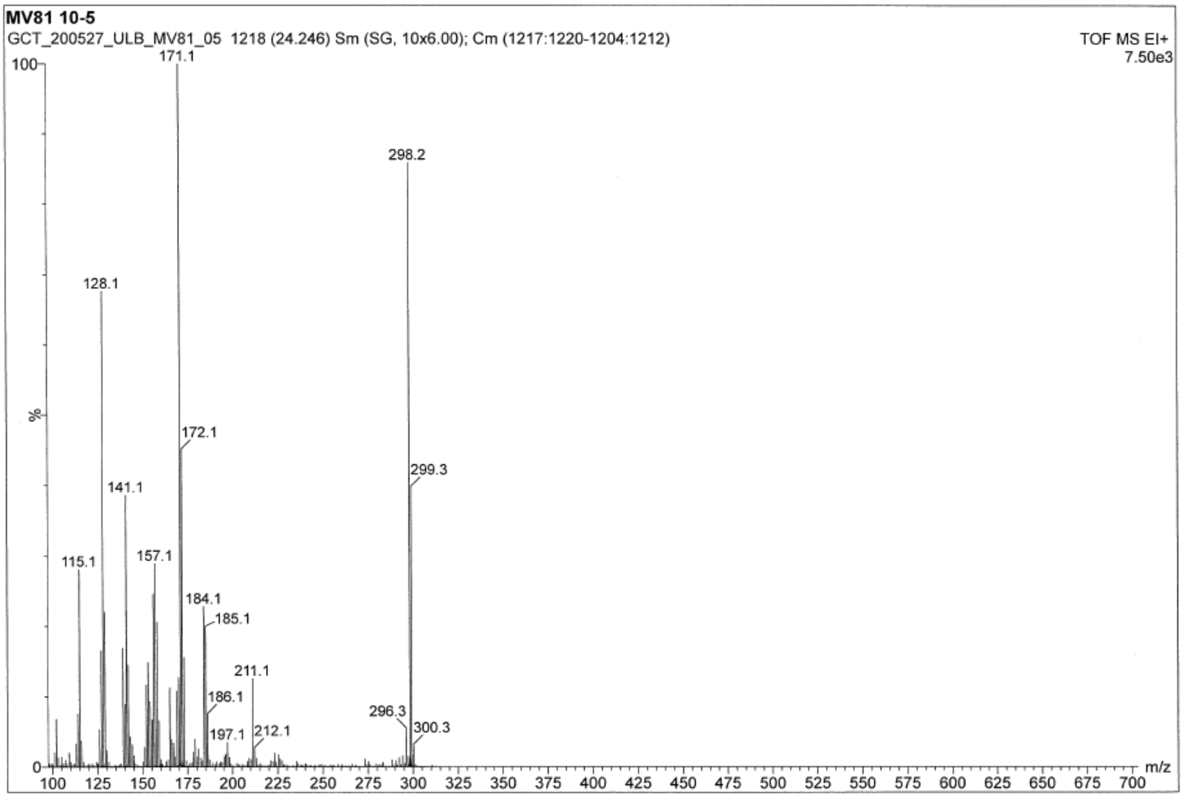

Figure S16 - HRMS spectra of 4R and 4S

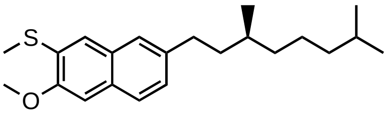

5R

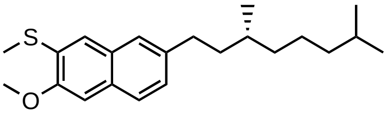

5S

Chemical Formula: $C_{22}H_{32}OS$
Exact Mass: 344,22
Molecular Weight: 344,56

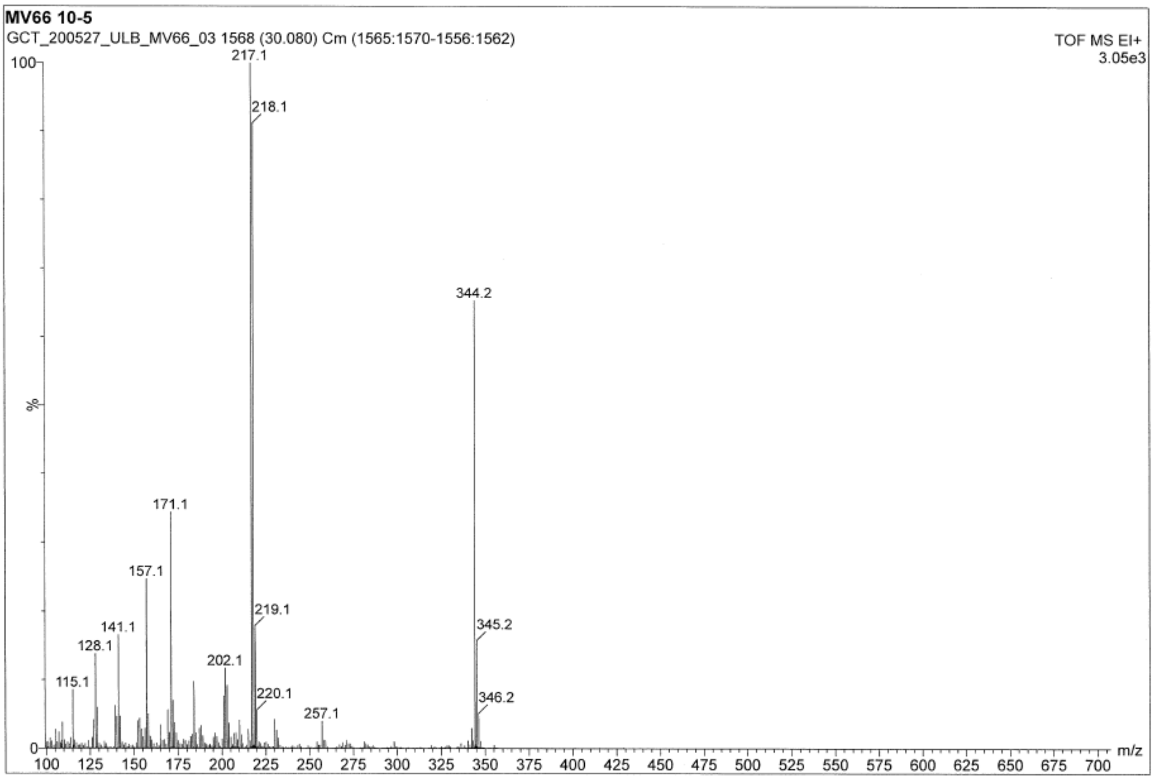

Figure S17 - HRMS spectra of 5R and 5S

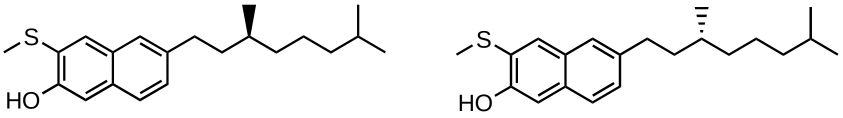

6R **6S**

Chemical Formula: $C_{21}H_{30}OS$
Exact Mass: 330,20
Molecular Weight: 330,53

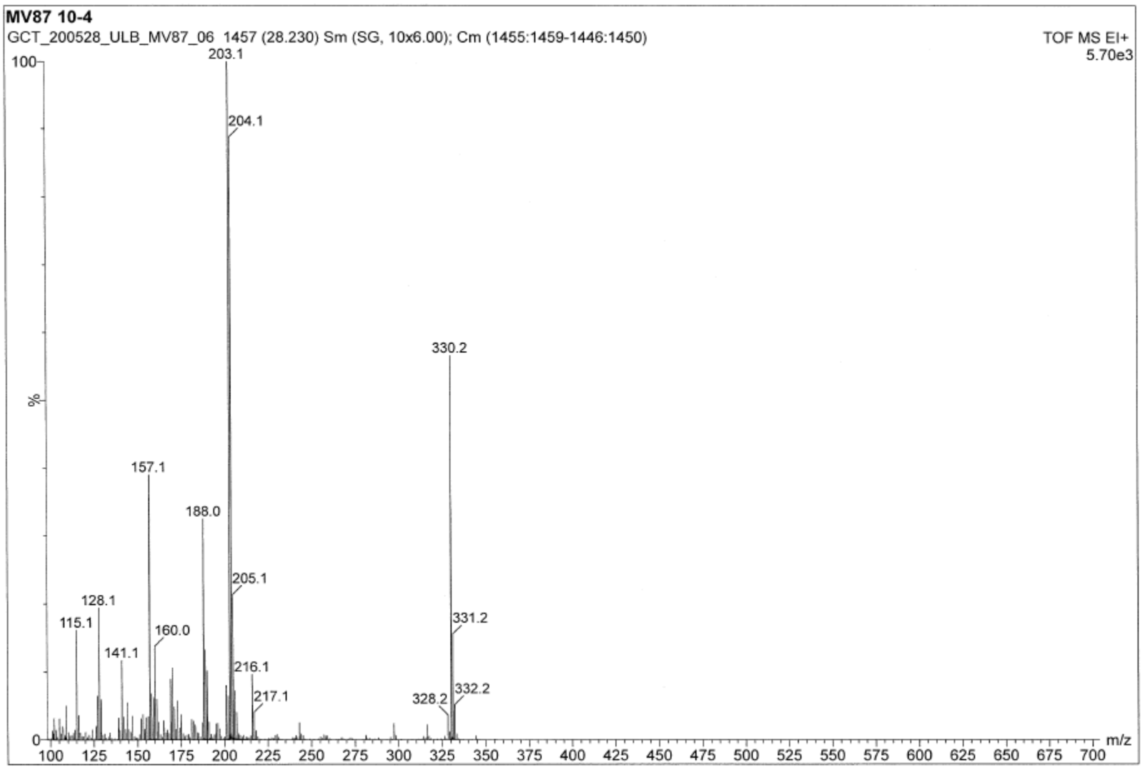

Figure S18 - HRMS spectra of 6R and 6S

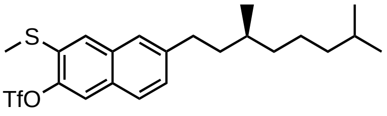

7R

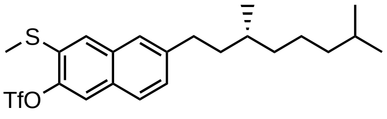

7S

Chemical Formula: $C_{22}H_{29}F_3O_3S_2$
Exact Mass: 462,15
Molecular Weight: 462,59

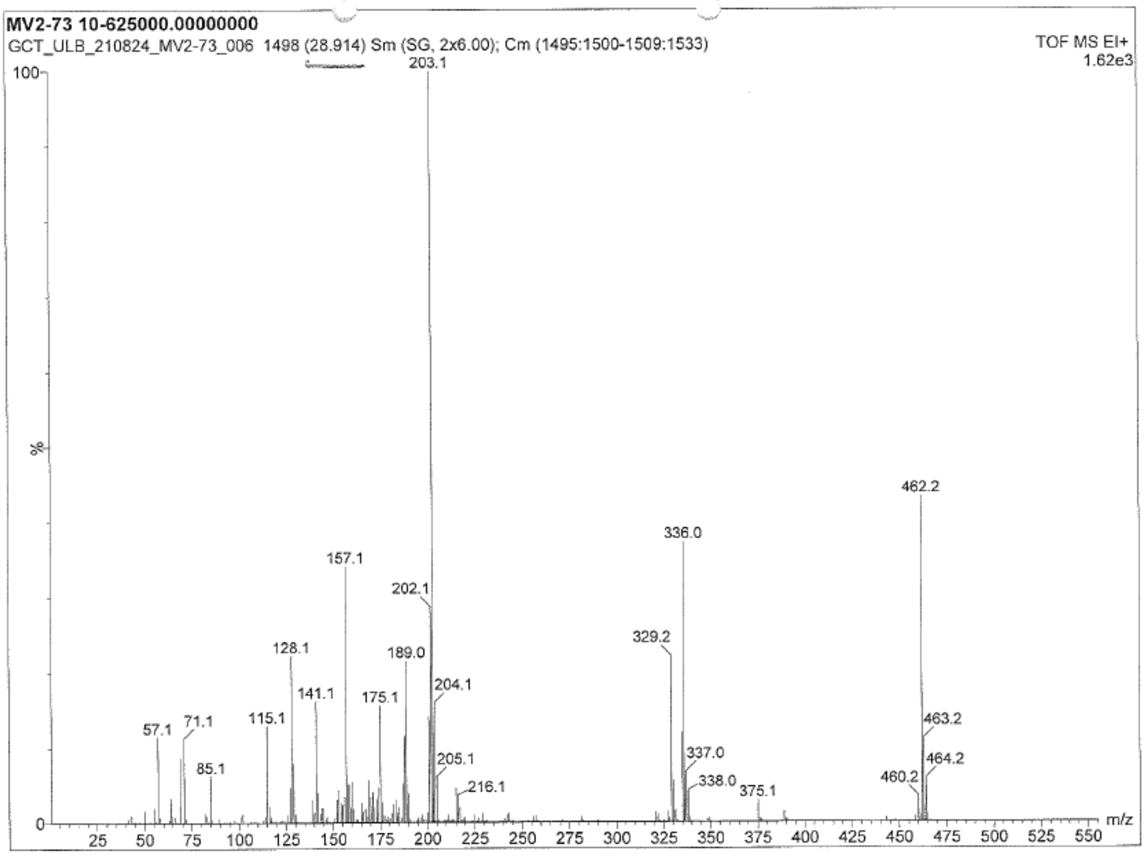

Figure S19 - HRMS spectra of 7R and 7S

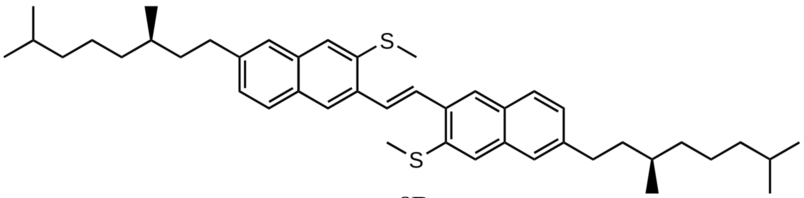

8R

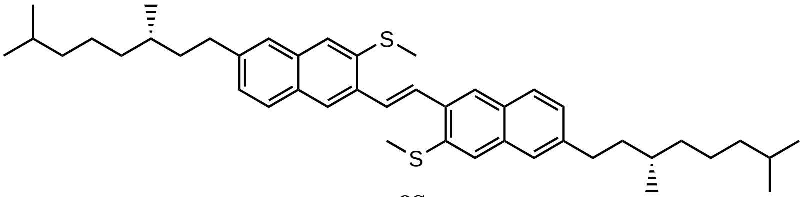

8S

Chemical Formula: $C_{44}H_{60}S_2$
Exact Mass: 652,41
Molecular Weight: 653,08

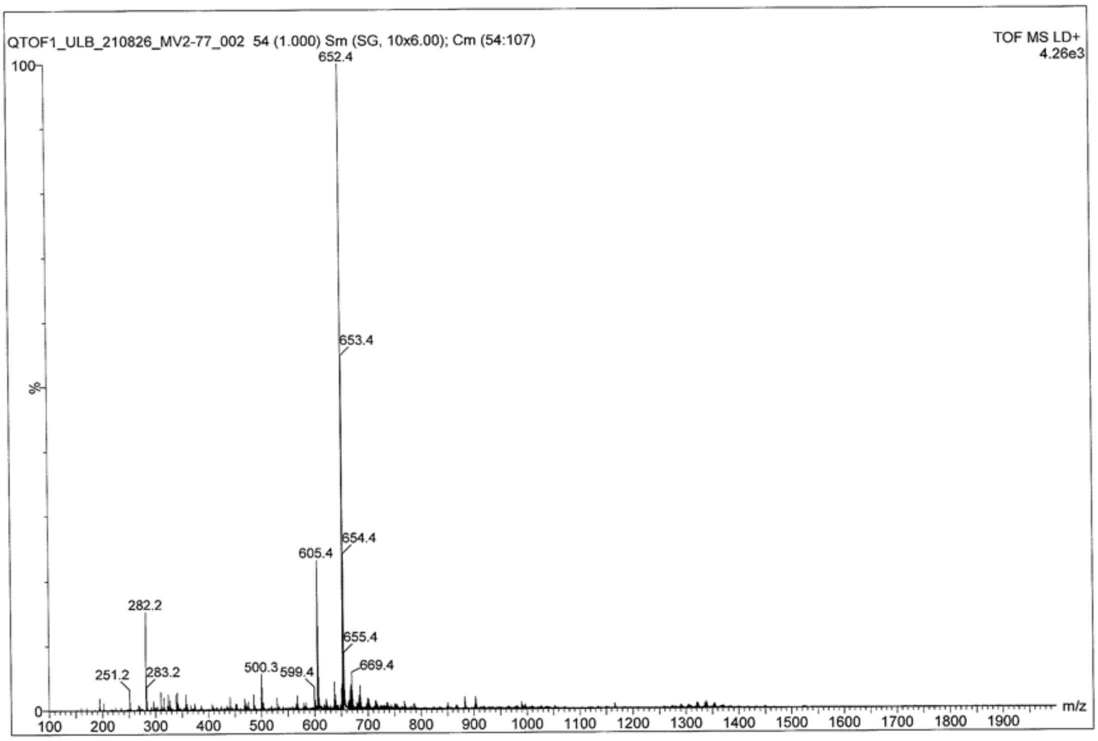

Figure S20 - HRMS spectra of 8R and 8S

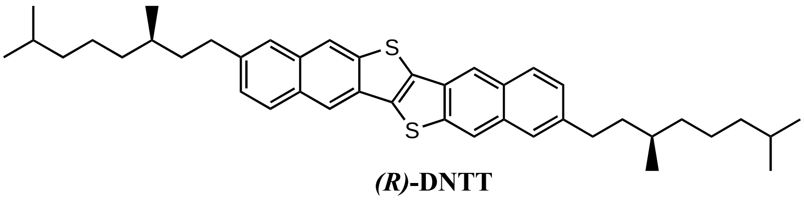

***(R)*-DNTT**

Chemical Formula: $C_{42}H_{52}S_2$
Exact Mass: 620,35
Molecular Weight: 621,00

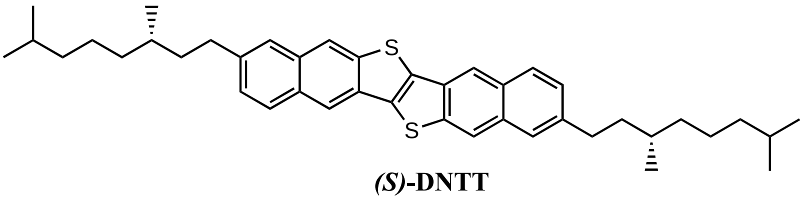

***(S)*-DNTT**

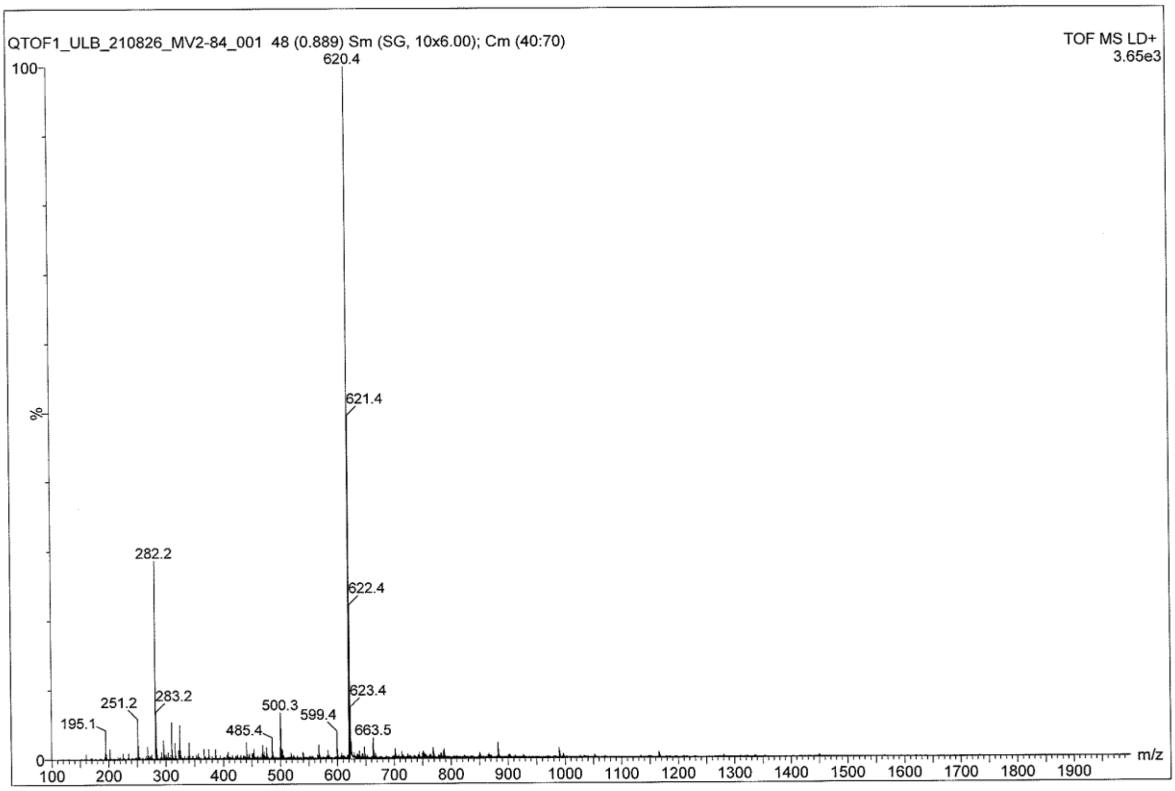

Figure S21 - HRMS spectra of (R)-DNTT and (S)-DNTT

1.4 Experimental UV-Vis absorption spectra

The optical absorption spectra, shown in Figure S22, were recorded with a Perkin Elmer's Lambda 950UV/Vis/NIR spectrophotometer. A background correction was performed, prior to measurement, for 100% transmittance or 0 absorbance over the wavelength range. The spectra of *(R)*-DNTT, *(S)*-DNTT and *(R+S)*-DNTT were taken in a chloroform solution.

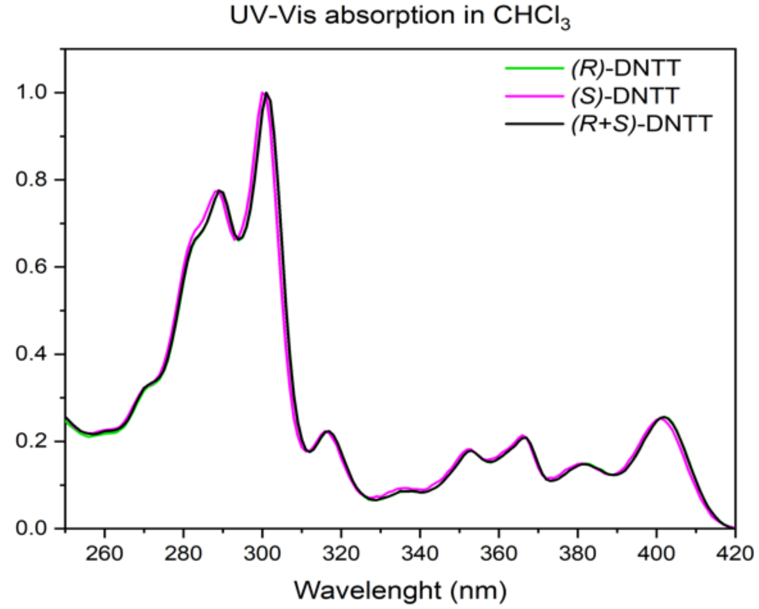

Figure S22 - UV-Visible absorption spectra of (R)-DNTT, (S)-DNTT and (R+S)-DNTT in a chloroform solution

1.5 Enantioenrichment by recrystallization

Enantiopurification has been achieved by recrystallization, after assessing chemical purity by NMR (Figure S14) and mass spectrometry (Figure S21). The commercially available chiral chains have an enantiomeric excess (*ee*) of 95% and 99% for *(R)*-DNTT and *(S)*-DNTT, respectively. The coupling of two chiral side chains to a single DNTT core decreases the *ee* of the product compared to the starting chiral chains. In Table S1 is reported the composition of the crude product after the synthesis of *(R)*-DNTT and *(S)*-DNTT, based on the *ee* of the chiral chains. The desired enantiomer is the most abundant compound, followed by the meso compound *(R),(S)*-DNTT and then by the opposite enantiomer. After each recrystallization the melting point of the compound has been measured by DSC. Four recrystallization steps are sufficient to reach the same value of melting point for the two enantiomers. In Figure S23 are reported the melting points and the yields of recrystallization associated to each step of the enantiopurification. The recrystallizations have been carried out from a saturated solution in boiling toluene. The hot solution has been gradually cooled down to room temperature overnight and the resulting crystalline fine yellow powder has been filtered under vacuum. In Figure S24 and Figure S25 are reported DSC traces corresponding to each recrystallization step. DSC traces of the final products after recrystallization n. 4 are reported in Figure S30 and Figure S31.

*Table S1 - Calculated ee of (R)-DNTT, (S)-DNTT, and meso compound (R),(S)-DNTT before enantioenrichment by recrystallisation of the product of the synthesis of (R)-DNTT and (S)-DNTT based on the initial ee of the commercial starting materials: 95% and 99%, for (R)-DNTT and (S)-DNTT, respectively. It is assumed that the ee of the intermediate products **2-7** remain constant throughout the synthetic steps. The ratio between stereoisomers for the coupling product **8** is also assumed to be the same than for (R)-DNTT, (S)-DNTT, and meso compound (R),(S)-DNTT.*

	***(S)*-DNTT**	***(R)*-DNTT**
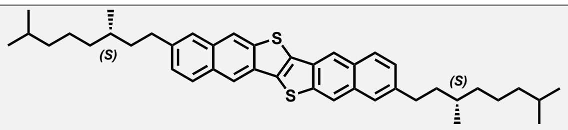	98.01%	0.25%
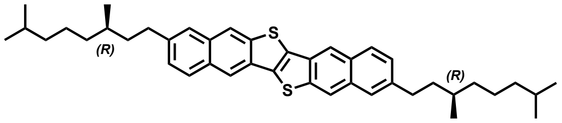	0.01%	90.25%
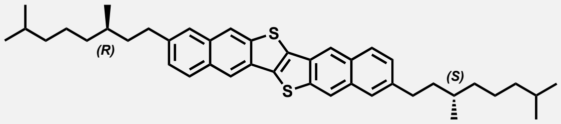	1.98%	9.5%

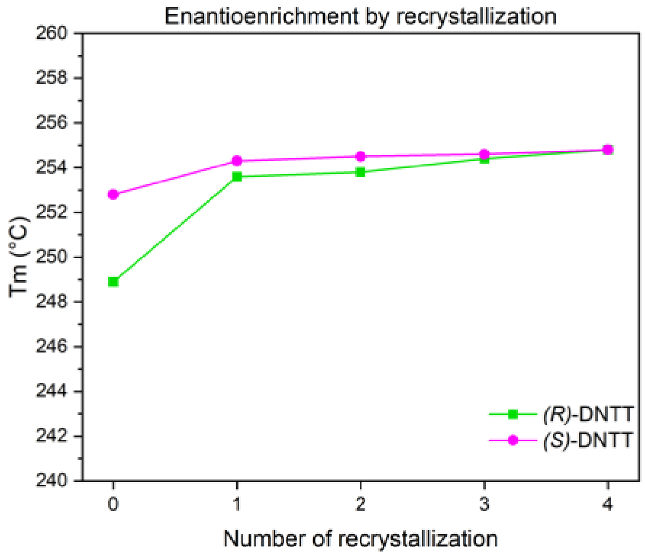

	(R)-DNTT		*(S)*-DNTT	
number of recryst	Tm (°C)	yield (%)	Tm (°C)	yield (%)
0	248.9	/	252.8	/
1	253.6	91	254.3	90
2	253.8	94	254.5	92
3	254.4	96	254.6	99
4	254.8	97	254.8	98

Figure S23 – Melting temperature and recrystallization yield for each recrystallization step of (R)-DNTT and (S)-DNTT

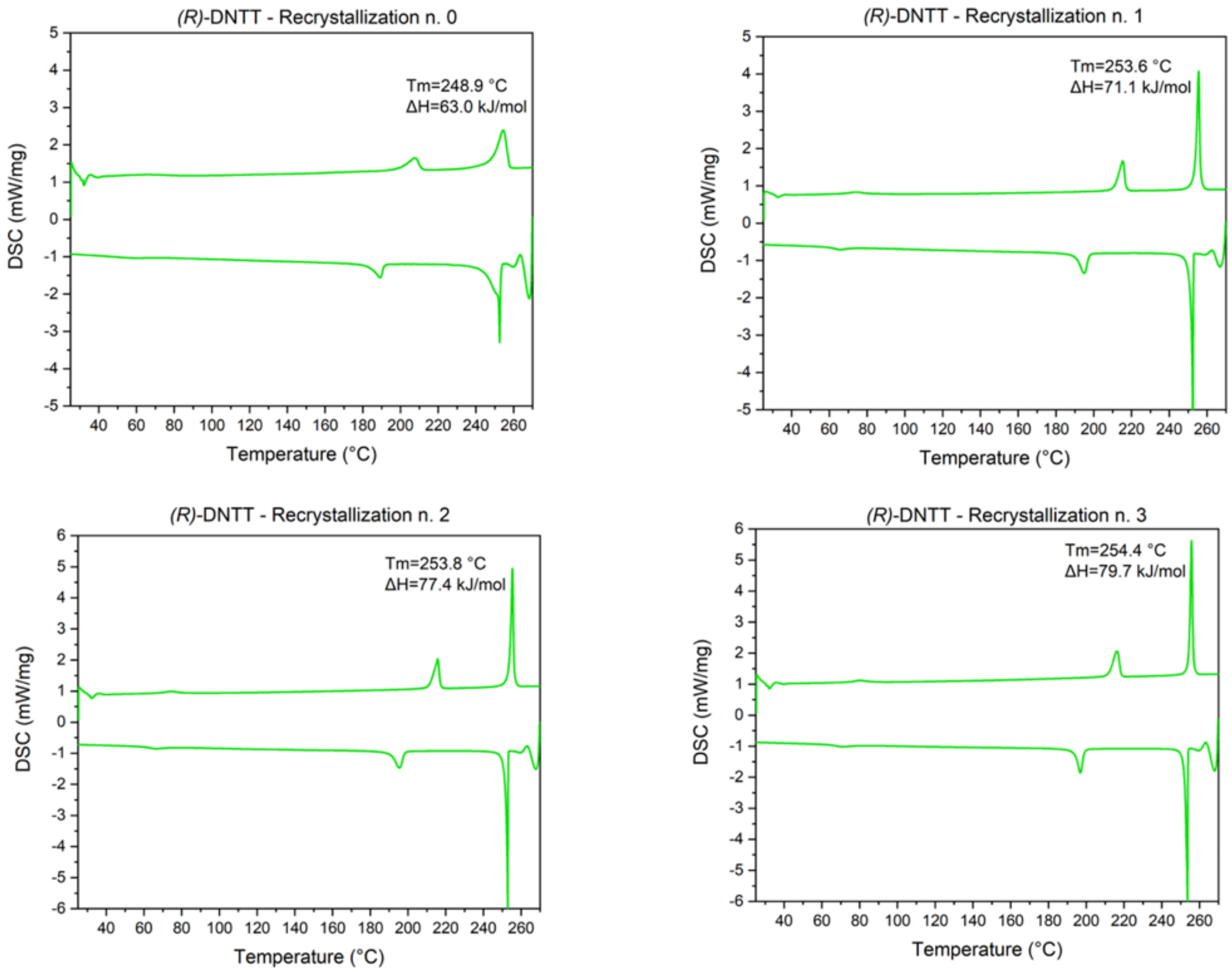

Figure S24 - DSC traces of (R)-DNTT for each recrystallization step mentioned in Figure S23

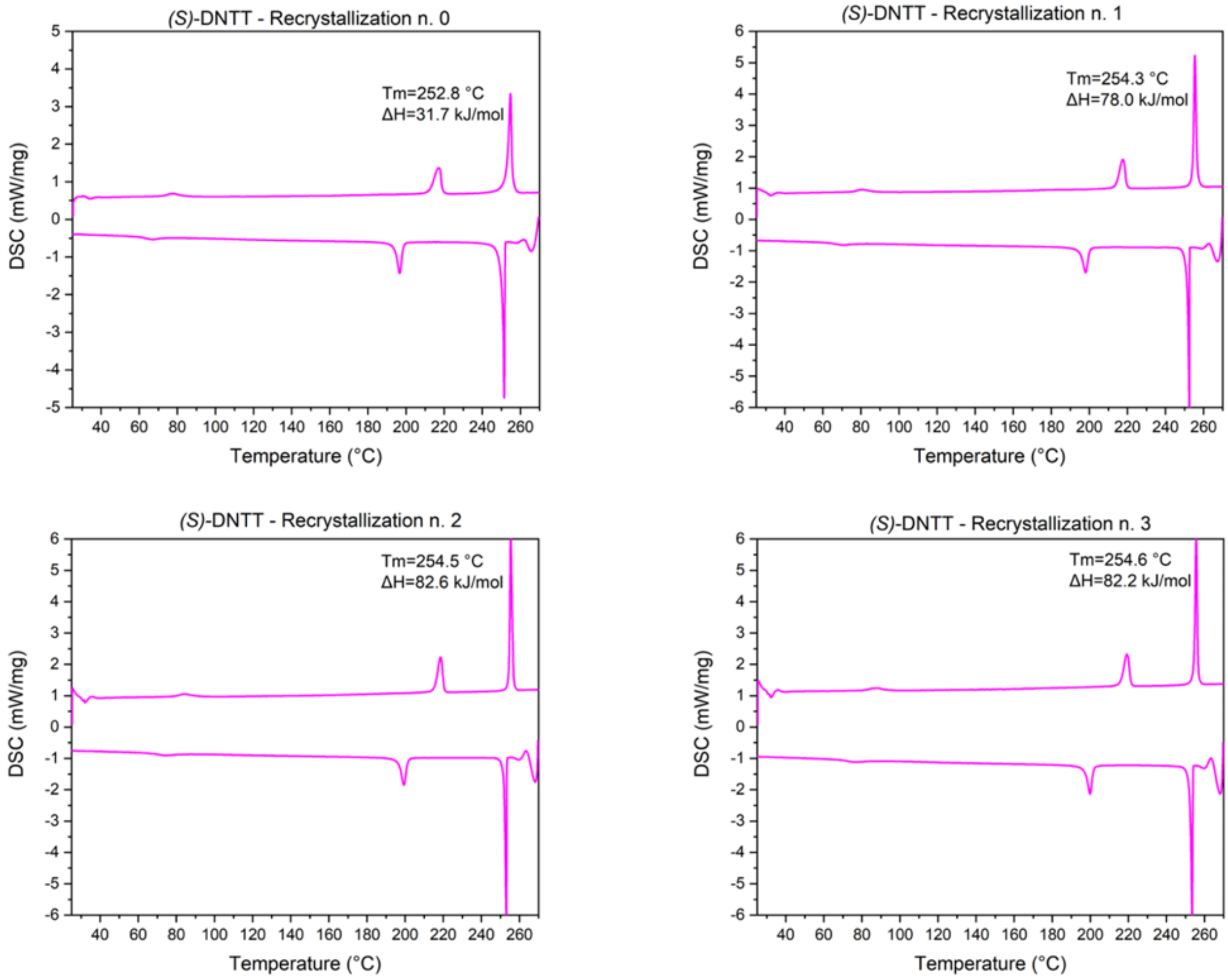

Figure S25 - DSC traces of (S)-DNTT for each recrystallization step mentioned in Figure S23

1.6 Preparation of the racemic mixture *(R+S)*-DNTT

The racemic mixture *(R+S)*-DNTT was prepared by dissolving equal quantities of *(R)*-DNTT and *(S)*-DNTT in $CHCl_3$ at room temperature, then refluxing the solution under argon for 10 minutes and letting it gradually cool down to RT. After a couple of days, the resulting crystalline powder was filtered under vacuum.

2. UPS and XPS measurements

X-ray and Ultraviolet photoelectron spectroscopy measurements were performed in ultra-high vacuum, on thin films of *(R)*- and *(S)*-DNTT that were thermally evaporated on sputtered clean polycrystalline gold substrates. The spectra were recorded on a hemispherical SPECS Phoibos 100 analyzer. The analyzer was calibrated prior to measurements by measuring the Fermi edge of a sputtered clean Au(111) substrate. For the XPS, we used magnesium K_a X-rays with an energy of 1253.6 eV to access the core energy levels of the atoms. To investigate the valence molecular energy levels, we used the Helium I_a emission line of 21.22 eV energy produced by a Helium discharge lamp. A UV-monochromator is installed at the UPS source to rule out the contribution of other emission lines of Helium to the spectrum.

2.1 XPS Measurements and elemental analysis

The chemical purity of the two compounds was determined using XPS. First, we obtained survey photoelectron spectra to determine the elemental composition of the samples, and then we scanned narrow energy regions that correspond to the peaks of carbon 1s (C1s) and sulfur 2p (S2p) to quantify the peak areas and determine the molecular stoichiometry. Figure S26 depicts survey scan spectra acquired with a pass energy of 50 eV, allowing an energy resolution of 1.2 eV. Figure S27 depicts narrow scan spectra of the Carbon 1s and Sulfur 2p energy regions recorded with a pass energy of 20 eV which provides a higher energy resolution of 0.8 eV.

The survey photoelectron spectra of *(R)*- and *(S)*- DNTT correspond to thin of 10 nm nominal thickness. The spectra show the characteristic chemical profiles of carbon and sulfur without the presence of external contamination. A minor signal associated with electrons excited from the Au 4f electronic level is still visible at the lower binding energy side of the *(S)*-DNTT spectrum and originates from the gold substrate. In the case of the *(R)*-DNTT survey spectrum, no signal from the underlying gold substrate is observed probably due to the full coverage of the gold substrate after deposition of 10 nm nominal thickness.

The photoelectron peak area, which represents the number of electrons recorded is proportional to the number of atoms in the given state. Therefore, from the ratio of the areas of the photoelectron peaks, it is possible to determine the stoichiometry of the material. To determine the peak areas, we followed a least-squares fitting procedure for the C1s and S2p peaks. All peaks were approximated with Voigt functions which are the convolution of a Gaussian and a Lorentzian function. The Gaussian function accounts for instrumental broadening, whereas the Lorentzian is associated with the natural linewidth of the energy levels. An active Shirley background was subtracted from the C1s and S2p peaks to remove the contribution of the inelastically scattered electrons (secondary electrons) to the peak areas.[2] Figure S27shows the approximated photoelectron peaks of the C1s and S2p core levels.

Both compounds have the same chemical formulas, thus the same stoichiometries ($C_{42}H_{52}S_2$). More specifically, the carbon to sulfur stoichiometry is given by:

$$\frac{C}{S} = \frac{42}{2} = 21$$

Equation S1

The experimental stoichiometry determined by XPS is given by:

$$\frac{C}{S} = \frac{A_{C1s}/S_{C1s}}{A_{S2p}/S_{S2p}}$$

Equation S2

where A_{C1s}, A_{S2p} are the areas of the C1s and S2p peaks respectively. The photoelectron peak areas were corrected using the relative sensitivity factors S_{C1s} and S_{S2p} for the C1s and S2p peaks, respectively. Theoretical values of the relative sensitivity factors values that we used are found in the previous study by Seah *et al.*[3] Table S2 and Table S3 show the least-squares fitting results for the C1s and S2p peaks of *(R)*-DNTT and *(S)*-DNTT, respectively.

Therefore, using Equation S2 and the experimental peak areas of C1s and S2p3/2 we find the stoichiometry of the two compounds as follows:

(R)-DNTT: $$\frac{C}{S} = \frac{A_{C1s}/S_{C1s}}{A_{S2p}/S_{S2p}} = 20.7 \pm 0.5$$

Equation S3

(S)-DNTT: $$\frac{C}{S} = \frac{A_{C1s}/S_{C1s}}{A_{S2p}/S_{S2p}} = 21.1 \pm 0.8$$

Equation S4

The observed stoichiometry is in reasonable agreement with the theoretical value within the experimental technique's confidence interval. The small discrepancy can be attributed to the S2p energy level's weak photoelectron signal.

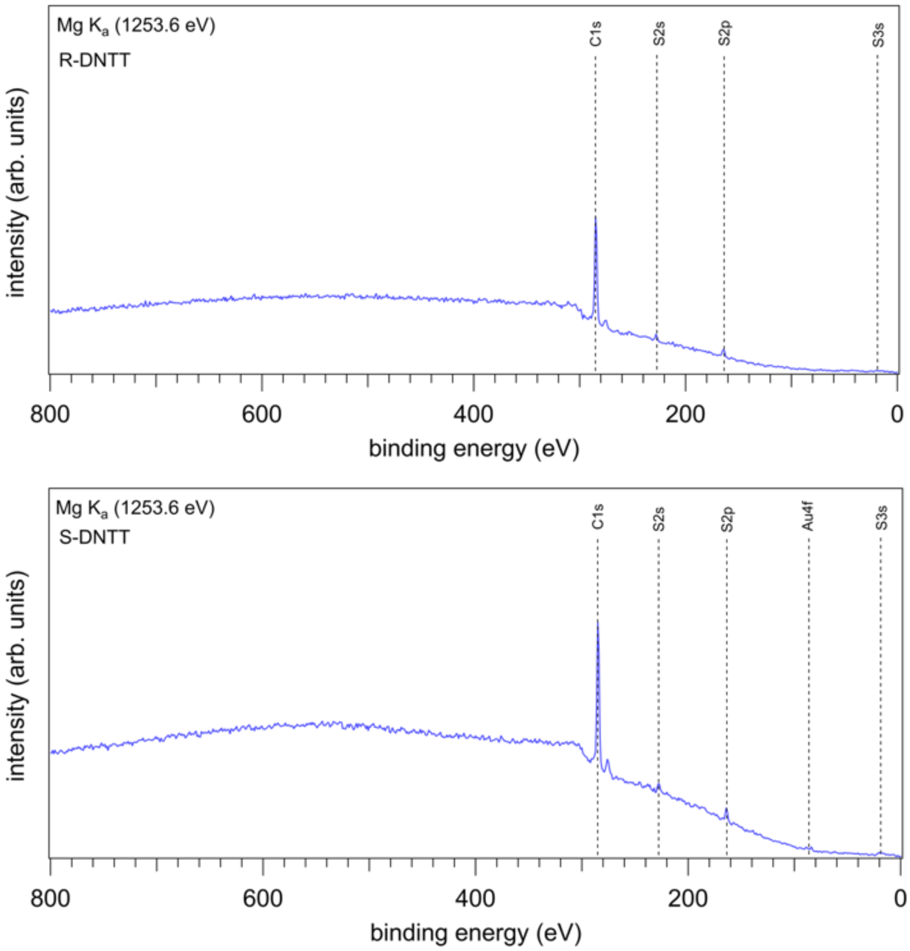

Figure S26 - XPS survey scan spectra from (R)-DNTT and (S)-DNTT. Thin films of 10 nm nominal thickness were deposited by thermal evaporation on polycrystalline gold substrates. The spectra were recorded using the Ka X-rays of magnesium with excitation energy of 1253.6 eV. The spectra show the elemental composition of the films. The vertical dashed lines indicate all the peaks present in the survey spectrum corresponding to the characteristic energy levels of the elements constituting (R)-DNTT and (S)-DNTT.

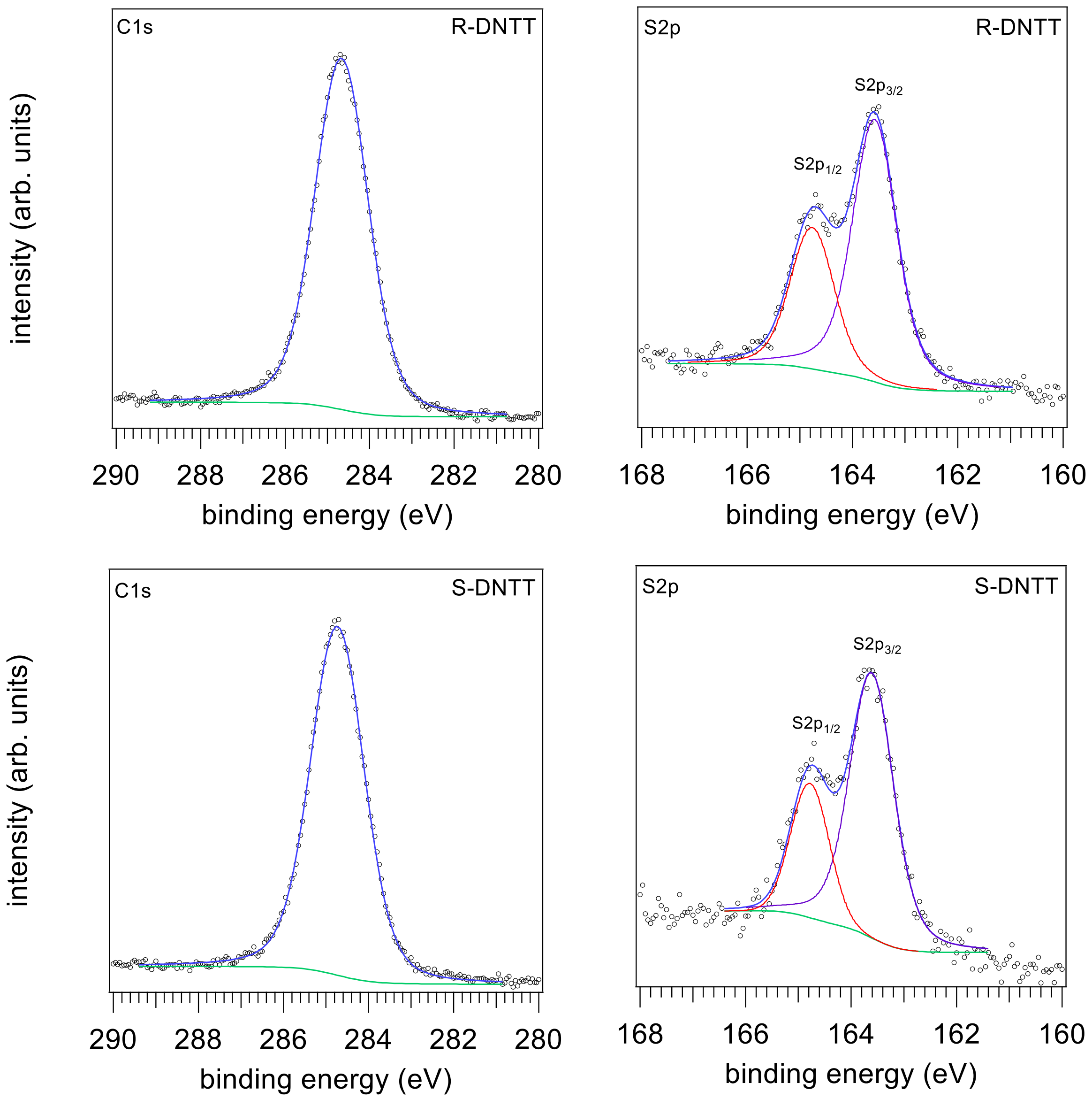

Figure S27 - Higher resolution narrow scan XPS spectra of the C1s and S2p energy levels of carbon and sulfur found in (R)-DNTT and (S)-DNTT molecules. The empty black circles indicate the raw data points while the blue line the least-squares fitted curve. The green line shows the Shirley background which was subtracted to evaluate the peak areas. The red and purple lines of the S2p peak correspond to the 2p3/2 and 2p1/2 energy levels of sulfur due to the spin-orbit splitting.

Table S2 - Fitting parameters and sensitivity factors for C1s and S2p peaks of (R)-DNTT

(R)-DNTT	location (eV)	peak area	FWHM (eV)	sensitivity factor
C1s	284.6 ± 0.4	2983.03 ± 14.82	1.49 ± 0.4	0.000181
S2p3/2	163.5 ± 0.4	172.49 ± 4.96	0.99 ± 0.4	0.000217
S2p1/2	164.7 ± 0.4	86.20 ± 4.84	0.99 ± 0.4	0.000111

Table S3 - Fitting parameters and sensitivity factors for C1s and S2p peaks of (S)-DNTT

(S)-DNTT	location (eV)	peak area	FWHM (eV)	sensitivity factor
C1s	284.7 ± 0.4	3956.53 ±19.53	1.49 ± 0.4	0.000181
S2p3/2	163.6 ± 0.4	224.45 ± 10.13	0.99 ± 0.4	0.000217
S2p1/2	164.8 ± 0.4	85.22 ± 9.33	0.85 ± 0.4	0.000111

2.2 Determination of ionization energies from UPS

To determine the ionization energy of *(R)*-DNTT and *(S)*-DNTT we performed ultraviolet photoemission measurements in ultra-high vacuum at a base pressure of 10^{-10} mbar. The valence region of both molecules was scanned using a pass energy of 5 eV that provides an energy resolution of 120 meV. For the work function which is obtained by the onset of the secondary electron cutoff, we used a pass energy of 2 eV that provides and energy resolution of 80 meV and we applied a sample bias of -10 V. Figure S28 shows the UPS spectra of the valence and secondary electron cutoff regions of *(R)*-DNTT and *(S)*-DNTT. Both molecules show very similar valence regions and resemble to the UPS valence spectrum of DNTT available in literature.[4] This is to be expected as the alkyl chains should not alter the electronic structure of the core molecule. The onset of the HOMO peak determines the hole injection barrier (HIB) and occurs at 0.87 eV for *(R)*-DNTT and 0.98 eV for *(S)*-DNTT. The samples work function (WF) is measured by the onset of the secondary electron cutoff which is found at 4.29 eV for *(R)*-DNTT and at 4.20 eV for *(S)*-DNTT. The ionization energy (IE) can then be calculated by the following expression:

$$IE = HIB + WF$$

Equation S5

Therefore, *(R)*-DNTT has an ionization energy of 5.16 ± 0.06 eV and *(S)*-DNTT an ionization energy of 5.18 ± 0.06 eV.

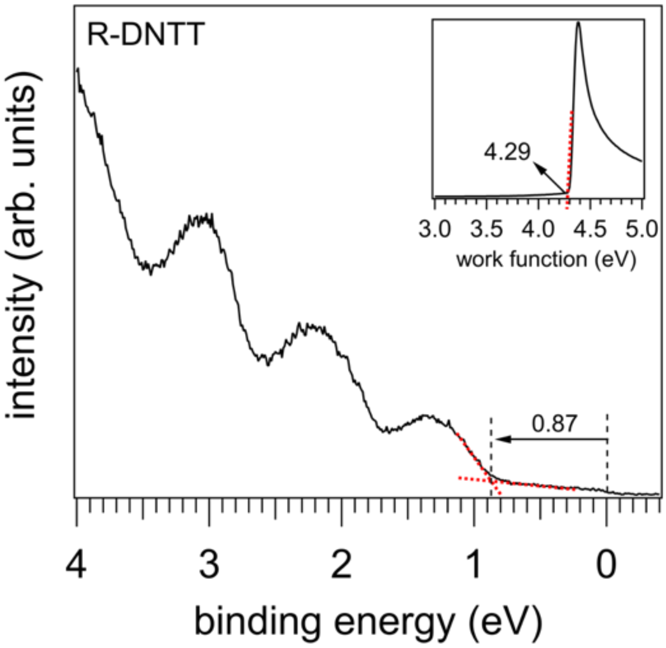

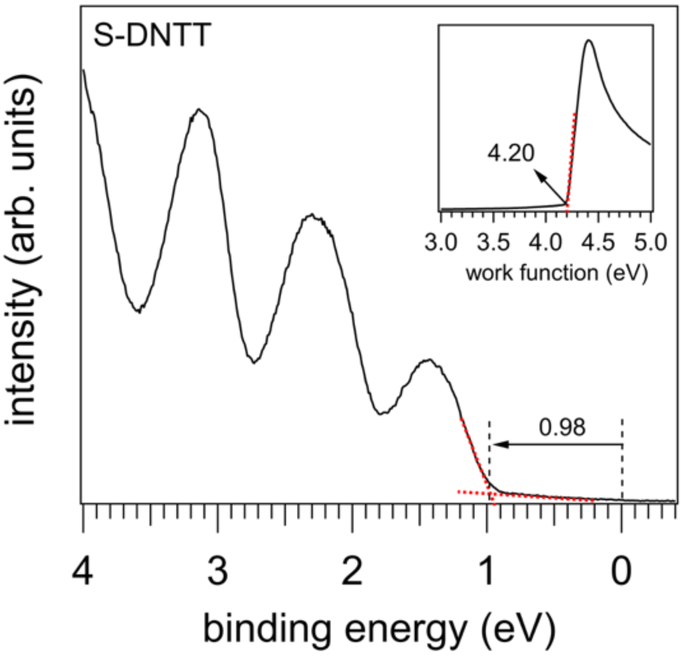

Figure S28 - UPS spectra showing the valence region of (R)-DNTT and (S)-DNTT thin films of 2 nm nominal thickness, deposited on polycrystalline gold substrates. The black arrows indicate the onset position of the HOMO peak from which we determine the hole injection barrier after a linear extrapolation method (red dashed lines). The insets in both graphs show the respective secondary electron cutoff regions from which we measured the work function of the (R)-DNTT and (S)-DNTT, by determining the low energy onset by a linear extrapolation method (red dashed line).

3. Determination of Ionization Energy from PYS

Photoelectron Yield Spectroscopy (PYS) in air exploits the photoelectric effect to determine the photoelectron emission yield (proportional to the number of extracted photoelectrons) from the OSC sample (in the form of thin film or powder) as a function of the incident light radiation energy. Photoelectron yield curves were collected using a Riken Keiki spectrophotometer (Japan) model AC-2 with energy step of 0.05 eV, a UV spot intensity of 10 nW, within an energy range of 3.4 to 6.2 eV. 12-nm-thick films fabricated on Al_2O_3 substrates by thermal deposition were used for determining the ionization energy (IE) of the OSCs. The final estimate for IE values is known with error of ± 0.05 eV or less.

IE value averaged over 3 measurements is 5.23 ± 0.01 eV for *(R)*-DNTT, 5.19 ± 0.01 eV for *(S)*-DNTT and 5.23 ± 0.01 eV for the racemic mixture *(R+S)*-DNTT (prepared according to the procedure reported in section 1.6). In the same experimental conditions, IE of reference compounds DNTT and C8-DNTT have been measured, being respectively 5.16 ± 0.04 eV and 4.94 ± 0.01 eV.

4. Thermal characterization

4.1 TGA

Thermogravimetric analysis (TGA) measurements were conducted on a Pyris 6 TGA instrument with Pyris software. About 5 mg of *(R)*-DNTT and *(S)*-DNTT were placed in an open aluminum crucible and were scanned at a rate of 10 °C/min under a nitrogen flow at 60 mL/min. TGA curves reveal that evaporation of both compounds starts around 370 °C showing that *(R)*-DNTT and *(S)*-DNTT possess high thermal stability (Figure S29).

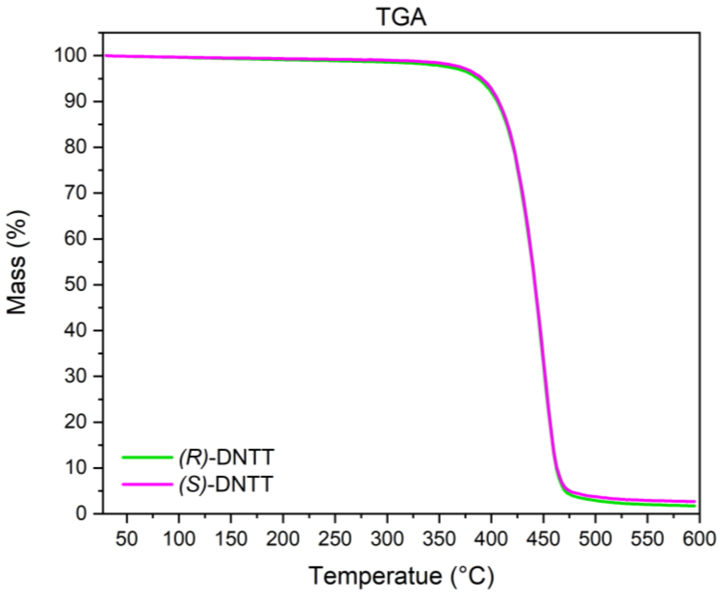

Figure S29 - TGA of (R)-DNTT and (S)-DNTT (heating rate 10 °C/min)

4.2 DSC

DSC analysis was performed on a Netzsch Polyma 214 instrument with Proteus software. Approximately 3 mg of compound were placed in a covered aluminum crucible with pierced lids. The heating and cooling rates of 10°C/min were adopted. The dry nitrogen acted as both purge and protect gas at 60 mL/min. Below are reported the DSC traces corresponding to the third heating/cooling cycle for *(R)*-DNTT, *(S)*-DNTT and *(R+S)*-DNTT (prepared according to the procedure reported in section 1.6). The two enantiomers *(R)*-DNTT and *(S)*-DNTT were previously purified with 4 recrystallization steps, according to the procedure reported in section 1.5. As anticipated a good agreement is observed between thermal data of *(R)*-DNTT and *(S)*-DNTT (Figure S30, Figure S31 and Table S4, Table S5).

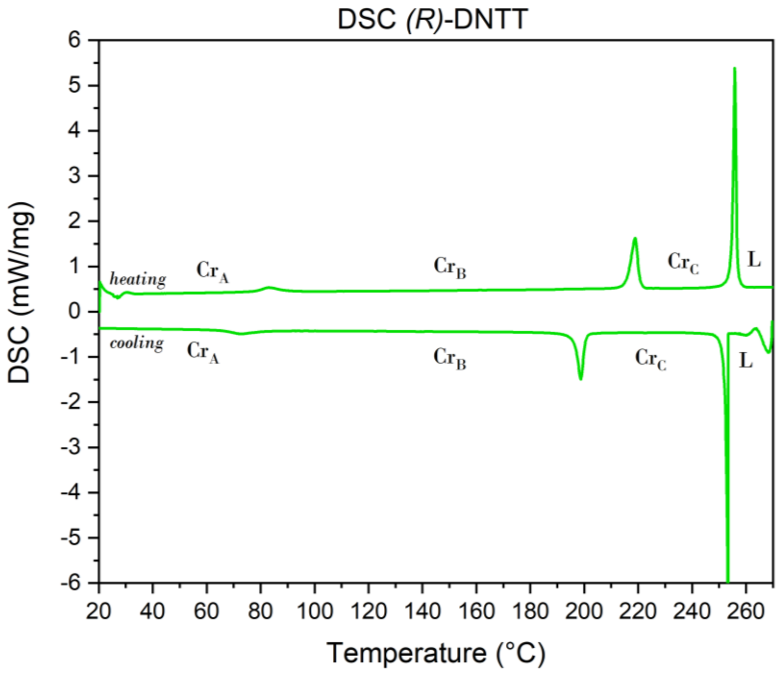

Figure S30 - DSC trace of (R)-DNTT recorded at 10 °C/min.

Table S4 – Description of peaks and transitions reported in DSC of (R)-DNTT

	***(R)*-DNTT**			
	Transition	Onset (°C)	Area (J/g)	ΔH (kJ/mol)
heating	$Cr_A \rightarrow Cr_B$	78,60	3,97	6,39
	$Cr_B \rightarrow Cr_C$	215,80	21,86	35,20
	$Cr_C \rightarrow L$	254,80	49,63	79,92
cooling	$Cr_B \rightarrow Cr_A$	78,50	-3,62	-5,83
	$Cr_C \rightarrow Cr_B$	200,50	-18,54	-29,86
	$L \rightarrow Cr_C$	253,40	-48,98	-78,87

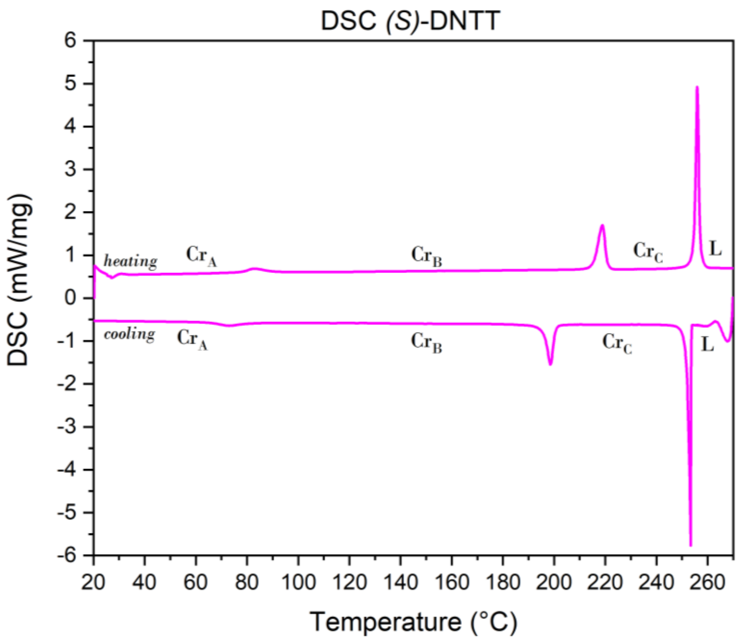

Figure S31 - DSC traces of (S)-DNTT recorded at 10 °C/min. Two heating/cooling cycles are reported.

Table S5 - Description of peaks and transitions reported in DSC of (S)-DNTT

	***(S)*-DNTT**			
	Transition	Onset (°C)	Area (J/g)	ΔH (kJ/mol)
heating	Cr_A→$Cr_{\mathbf{B}}$	79,10	3,01	4,85
	Cr_B→Cr_C	215,40	22,28	35,88
	Cr_C→L	254,80	49,66	79,97
cooling	Cr_B→Cr_A	78,40	-3,22	-5,19
	Cr_C→Cr_B	200,50	-18,05	-29,07
	L→Cr_C	253,60	-49,56	-79,81

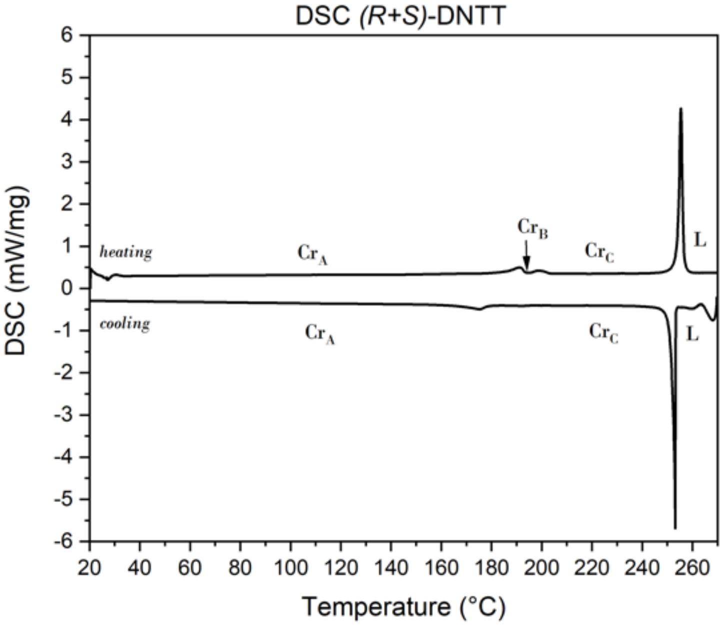

Figure S32- DSC trace of (R+S)-DNTT recorded at 10 °C/min.

Table S6 - Description of peaks and transitions reported in DSC of (R+S)-DNTT

	***(R+S)*-DNTT**			
	Transition	Onset (°C)	Area (J/g)	ΔH (kJ/mol)
heating	$Cr_A \rightarrow Cr_B$	187,80	3,90	6,27
	$Cr_B \rightarrow Cr_C$	195,30	1,56	2,51
	$Cr_C \rightarrow L$	254,00	48,17	77,57
cooling	$Cr_C \rightarrow Cr_A$	178,10	-4,11	-6,62
	$L \rightarrow Cr_C$	253,50	-47,41	-76,34

4.3 Hot-Stage Optical Microscopy

Images of Figure S33 were recorded at different temperatures under transmitted light with an optical microscope equipped with a hot-stage. A NIKON Eclipse 80i polarized light microscope equipped with a heating stage Linkam Scientific GS350 has been used in this work. Microscopy images of *(S)*-DNTT confirm that all the transitions in DSC traces correspond to crystal to crystal transitions, no liquid crystalline phase has been observed. The recorded pictures upon heating/cooling cycles always exhibit some sharp angles that are incompatible with classical textures observed for liquid crystal phases.[5]

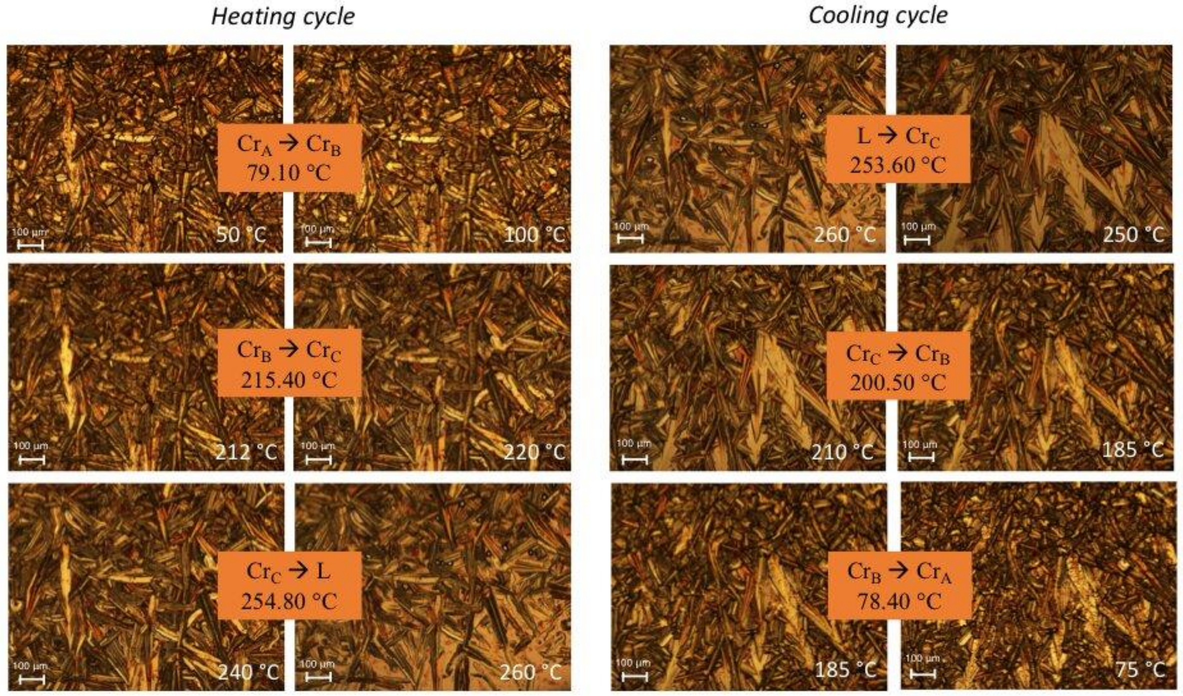

Figure S33 – Optical images of a sample of (S)-DNTT recorded at different temperatures using an hot-stage.

4.4 Variable temperature XRD

The variable-temperature PXRD patterns of the sample (Figure S34) were recorded using a DHS 1100 (Anton Paar Company) heating stage mounted on a Rigaku Ultima IV diffractometer, using Cu–Kα radiation (λ = 1.54178 I maÅ), a 2θ scan range 1.5°- 50°, a step size of 0.02° and a 10 °C heating increment. The PXRD recoded at 50 and 100 °C don't present any difference in the position of the first three 00*l* peaks, suggesting that the small Cr_A→Cr_B transition at 79.10°C in DSC (Figure S31) could be related to a rearrangement of the chiral side chains. A gradual shift towards higher 2θ value of the first three 00*l* diffraction peaks upon increasing the temperature from 190 °C to 240 °C (corresponding to the Cr_B→Cr_C transition at 215.40 °C in DSC) indicates an up to 2 Å contraction of the unit cell with temperature, along this specific crystallographic direction. The 002 peak, in the pattern recorded at room temperature, corresponds to a distance of 33 Å, which equates the length of the molecule with straight alkyl chains, in agreement with the 33.2 Å distance given by the structure solved by SC-XRD.

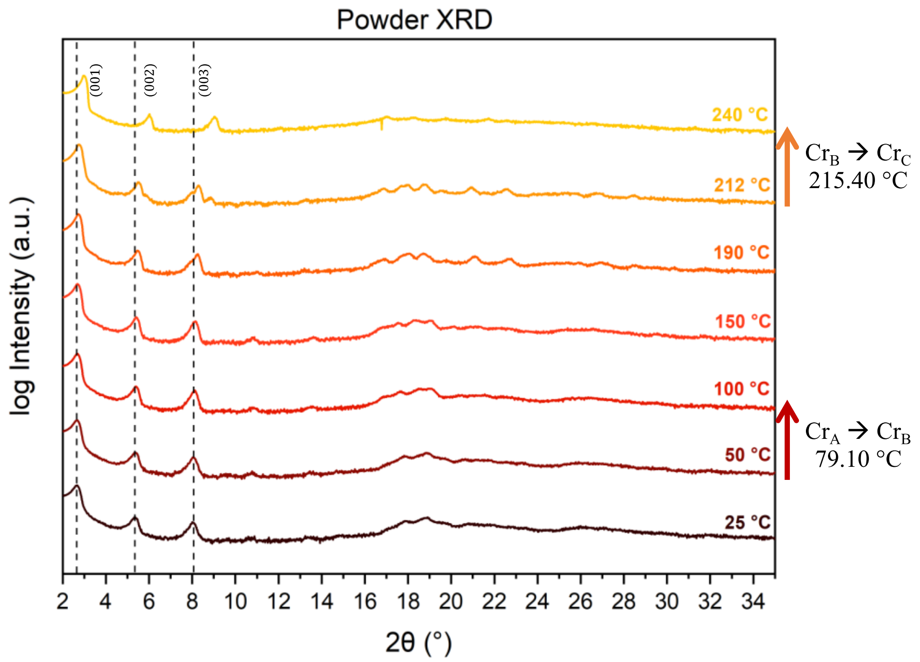

Figure S34 - PXRD of (S)-DNTT in function of temperature

5. CRYSTAL STRUCTURE

5.1 SC-XRD data

All crystallographic measurements were made with monochromatic Cu radiation (λ = 1.54184 Å) using a Rigaku Synergy-i diffractometer. Raw data processing utilised the program CrysalisPro.[6] All structures were solved using direct methods and were refined against F^2 to convergence using all unique reflections and the program Shelxl-2018,[7] as implemented within WinGX.[8] All four crystallographically independent alkyl groups were found to be disordered. Each was modelled over two sites with appropriate restraints and constraints applied to ensure that both geometry and displacement behaviour approached normal values. Selected crystallographic and refinement parameters are given in Table S7 and full structural and experimental details are available in cif format from the CCDC, deposition number 2239381.

Table S7 - Selected Crystallographic and Refinement Parameters

**Compound**	***(S)*-DNTT**
**Formula**	$C_{42}H_{52}S_2$
**Form. Wt.**	620.95
**Crystal system**	Triclinic
**Space group**	P1
**Temp. (K)**	295(2)
**a (Å)**	6.0392(2)
**b (Å)**	9.2522(3)
**c (Å)**	32.8958(11)
**α (°)**	90.513(2)
**β (°)**	91.336(2)

**γ (°)**	93.780(2)
**Volume (Å^3)**	1833.48(10)
**Z**	2
**Collected Reflections**	15574
**Independent Reflections**	8937
**Observed Reflections**	6171
**R_{int}**	0.0232
**2Θmax (°)**	143.016
**No. Parameters**	924
**S**	1.013
**R [on *F*, obs refs only]**	0.0590
**ωR [on F^2, all data]**	0.1825
**Largest diff. peak /hole (eÅ^{-3})**	0.246/-0.240
**Flack parameter**	0.012(19)

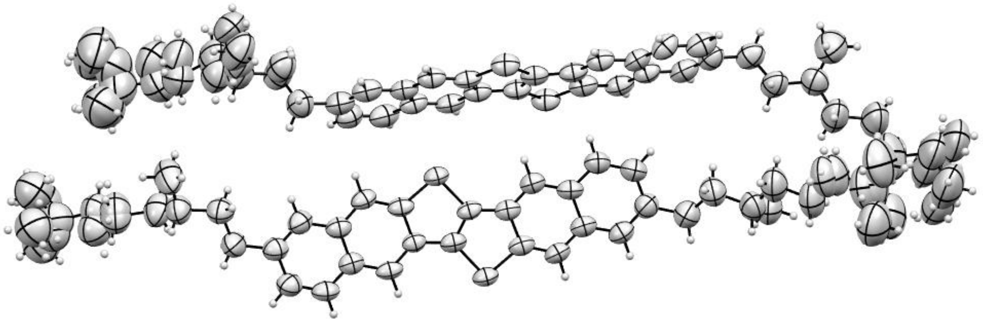

Figure S35 - Highlight of the disorder on the chiral alkyl chains

5.2 Hirshfeld surfaces analysis and fingerprints plot

The Hirshfeld surfaces[9] were generated through *CrystalExplorer17* software.[10] To handle the disorder on the chiral side chains in the experimental crystal structure, the surfaces have been calculated for the main conformer. In *CrystalExplorer*, X-H bond lengths were normalized to be the same as those obtained from a neutron diffraction experiments (e.g. C-H=1.083 Å).[11] The normalized contact distance (d_{norm}) based on d_{e} (the distance from the point on the surface to the nearest nucleus external to the surface) and d_{i} (the distance from the point on the surface to the nearest nucleus internal to the surface) and van der Waals radii of the atom is calculated by :

$$d_{norm} = \frac{(d_i - r_i^{vdw})}{r_i^{vdw}} + \frac{(d_e - r_e^{vdw})}{r_e^{vdw}}$$

Equation S6

where r_i^{vdw} and r_e^{vdw} are the van der Waals radii of the atoms. The parameter d_{norm} is negative or positive, which is illustrated by a surface with a red–white–blue colored graph. On the surface, red spots represent the lengths shorter than the van der Waals distance, whilst

white and blue regions show lengths around and longer than the van der Waals distance, respectively.
2D fingerprint plot derived from a Hirshfeld surface[9,12] reveals visually the frequency of each combination of d_e and d_i over the molecular surface. The color on the plot with a range from blue (relatively few points) through green (moderate fraction) to red (highest fraction) reflects the contribution from different interatomic contacts.
Curvedness surface,[9] a measure of ''how much'' shape is useful to measure curvature, offers further chemical insight into molecular packing. Low values of curvedness designate essentially a flat region of the surface and may be a sign of π…π stacking in the crystal. High curvedness is highlighted as dark-blue edges tends to divide the surface into patches. Curvedness, C, is given by:

$$C = \frac{2}{\pi} ln \sqrt{{k_1}^2 + {k_2}^2/2}$$

Equation S7

Where, k_1 and k_2 are principal curvatures.

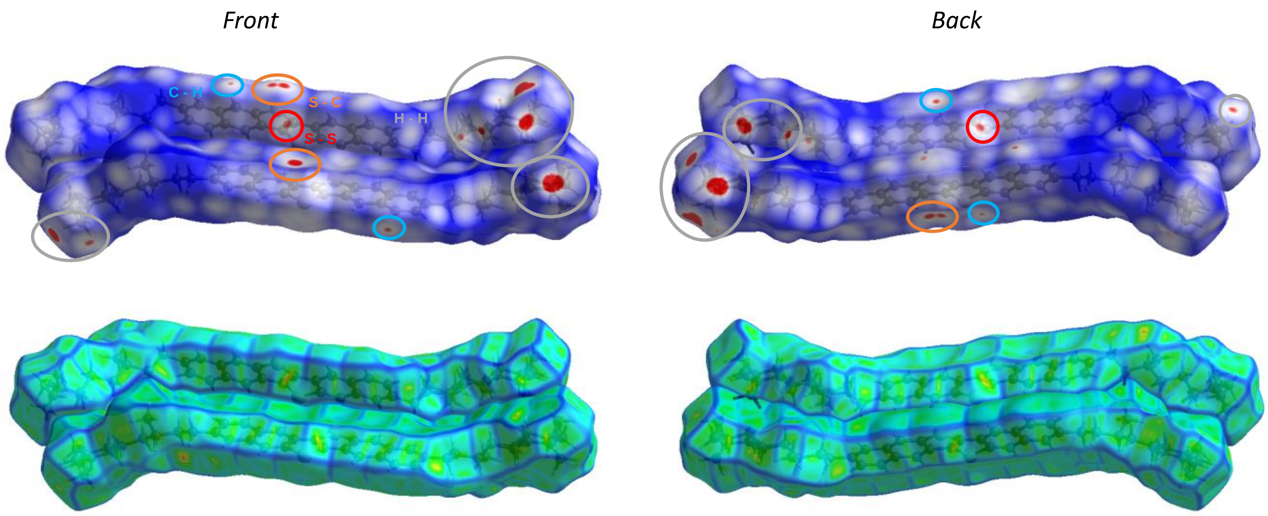

Figure S36 – Front (left) and back (right) views of Hirshfeld surfaces of the two unit cell molecules of (S)-DNTT (mapped over a d_{norm} range of -0.05 to 1.00 Å, top) and curvedness surfaces (bottom). Short contacts area are highlighted in Hirshfeld surfaces: S-S in red, S-C in orange, C-H in light blue and H-H in grey.

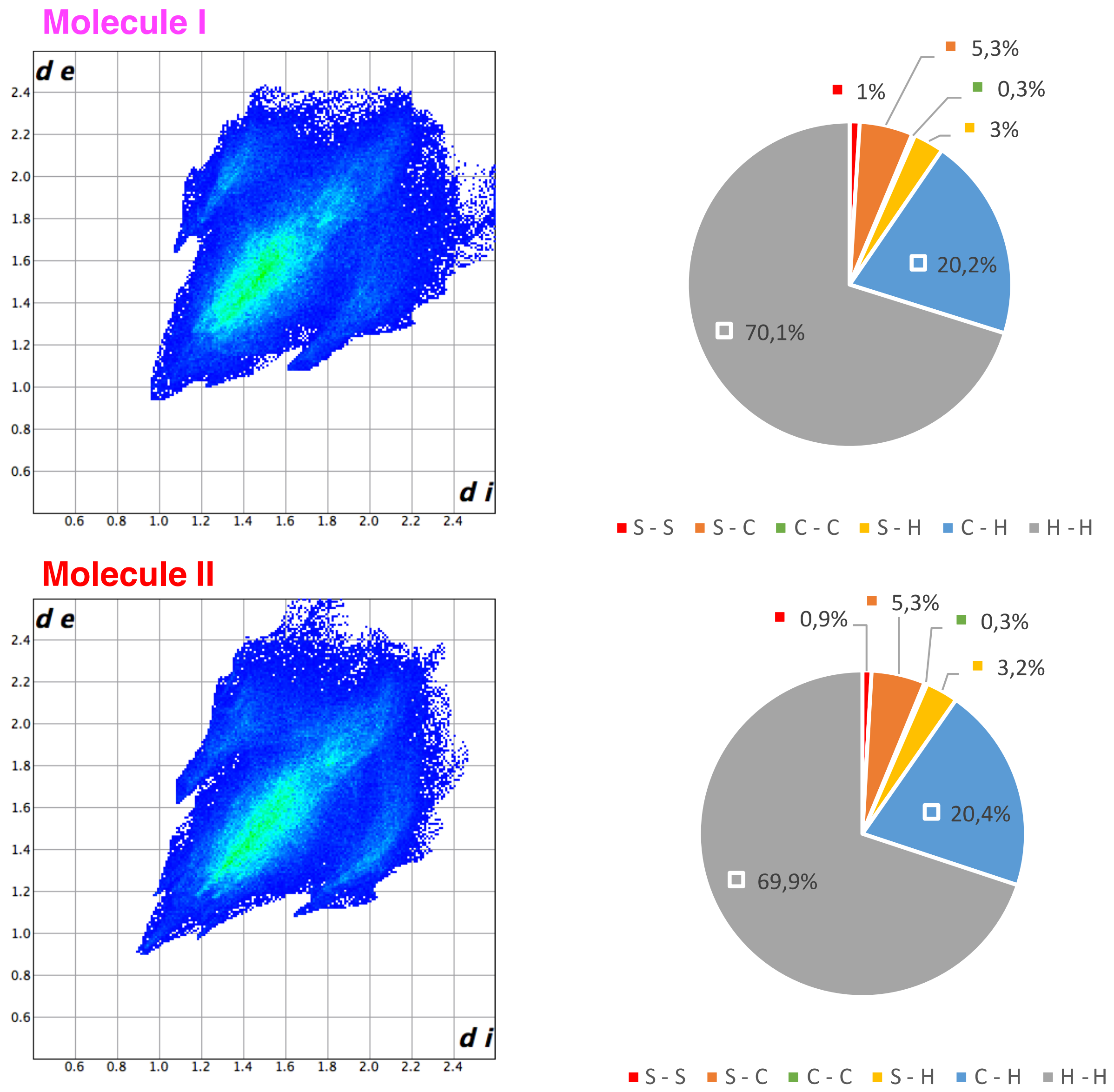

Figure S37 - 2D fingerprint plots of the two molecules in the unit cell (left) and the corresponding contacts contributions (right)

5.3 <u>Powder XRD</u>

PXRD patterns were recorded at room temperature with a Panalytical Empyrean diffractometer using Cu–Kα radiation (λ = 1.54178 Å), configuration = Reflection-Transmission Spinner 3.0, scan range 2°- 50°, step size = 0.0016°.

PXRD patterns of *(R)*-DNTT, *(S)*-DNTT and *(R+S)*-DNTT are displayed in Figure S38 in comparison with the pattern calculated from the SC-XRD data. The pattern of *(R+S)*-DNTT has the characteristic signatures of the pure enantiomers suggesting the formation of a conglomerate of *(R)*-DNTT and *(S)*-DNTT, but the phase behavior is complex. The doubling of the 00*l* peaks and the differences at high θ values are due to the presence of a mixture of phases and polymorphism. This behavior is confirmed also by the differences in DSC transitions (Figure S30, Figure S31, Figure S32).

In Figure S39 is reported the theoretical morphology of the grown crystal of *(S)*-DNTT calculated according to the Bravais-Friedel-Donnay-Harker (BDFH) method using Material

Studio. The crystal growth morphology shows the preferential orientation of the molecules along the c axes of the unit cell, which is in agreement with the molecular orientation suggested by the PXRD pattern.

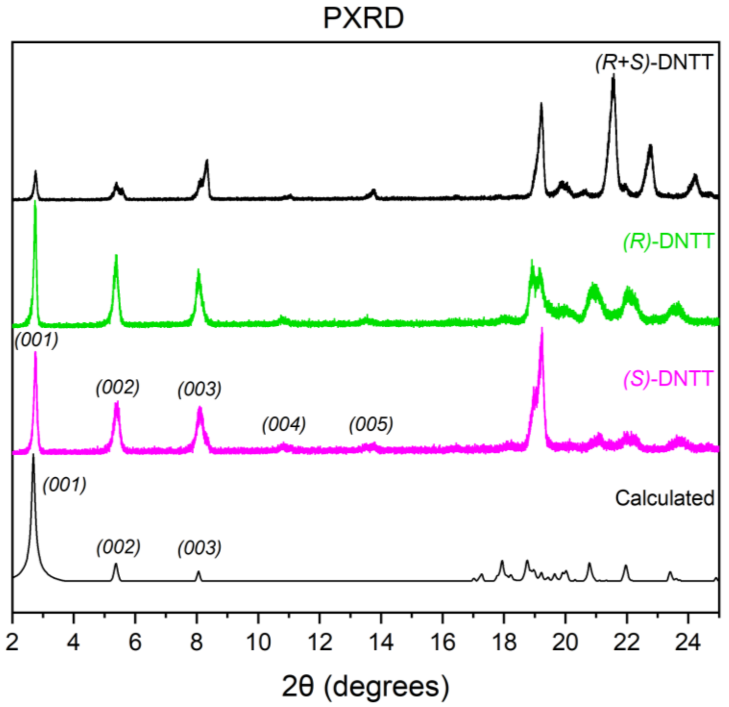

Figure S38 – PXRD patterns recorded at room temperature of (R)-DNTT, (S)-DNTT and (R+S)-DNTT in comparison with the pattern calculated from the SC-XRD structure data.

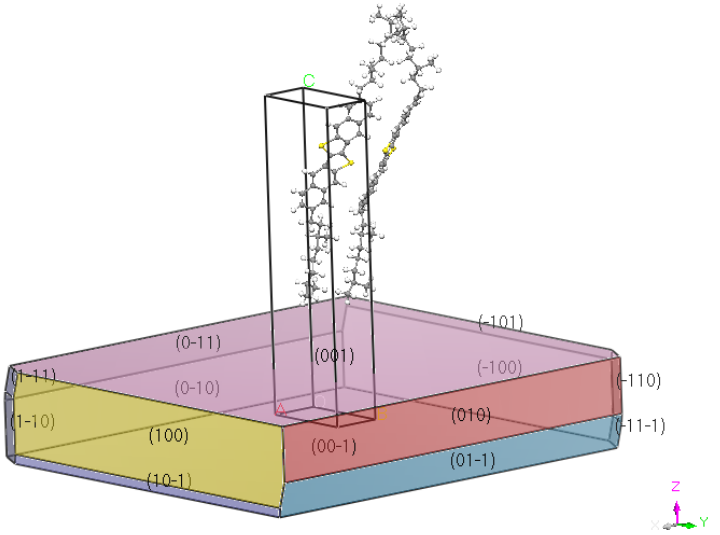

Figure S39 -Crystal growth morphology of (S)-DNTT calculated with Materials Studio software

6. QUANTUM CALCULATIONS

The Gaussian 16-A03 package was used to optimize the geometry, to compute the electronic structure of the neutral isomers and to estimate their reorganization energies at the Density Functional Theory (DFT) level using the B3LYP functional and a 6-311G* basis set.[13] The reported experimental crystal structure of *(S)*-DNTT was used as input to calculate transfer integrals between close neighbors in the frozen crystal structure. The ADF package was used to estimate these parameters at the DFT/B3LYP/DZ level of theory within a fragment orbital approach.[14,15] In practice, we created a 3×3×3 supercell for each molecule and computed electronic couplings among all pairs of close neighbors involving molecules in the unit cell at the center of the system. A close neighbor to a given molecule A is defined as any molecule B for which at least one atom is within a 5 Å range of any atom of molecule A.

6.1 HOMO/LUMO

HOMO and LUMO orbitals were calculated in vacuum on a single molecule with Gaussian at the B3LYP/6-311G* level of theory, after geometry optimization of the structure.

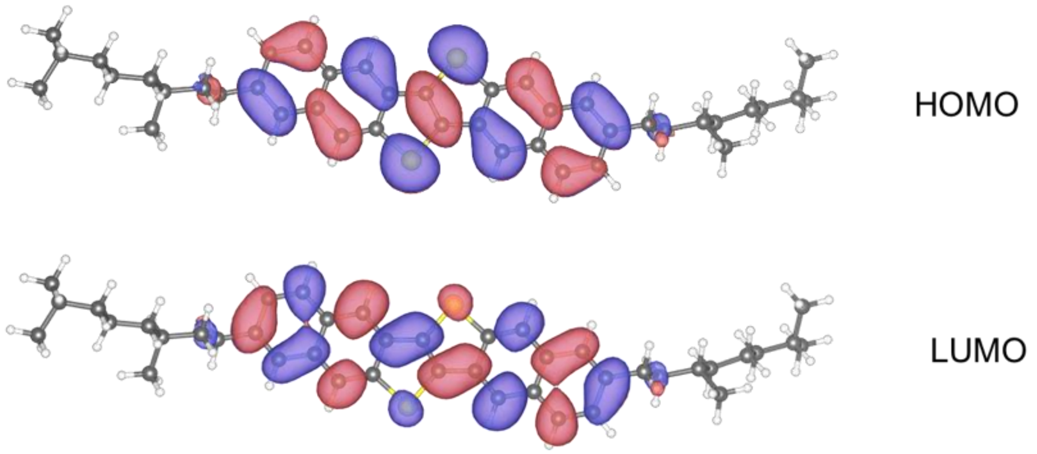

Figure S40 - Energy and shape of the HOMO and LUMO of (S)-DNTT calculated by DFT after geometry optimization at the B3LYP/6-311G level*

7. Organic Field-Effect Transistors with Au contacts

7.1 Device fabrication

All the thin film transistors were fabricated on highly doped silicon wafers with 30 nm of Al_2O_3 (Christian-Albrecht University of Kiel, Institute for Electrical Engineering and Information Technology). The Al_2O_3 substrates were exposed to oxygen plasma (Diener Electronic; oxygen flow rate 20 sccm, pressure 0.50 mbar, plasma power 100 W, duration 2 min) and then immersed overnight in 1.5 mM solution of n-tetradecylphosphonic acid (TDPA, Sigma-Aldrich) in 2-propanol (Acros Organics) to obtain a 1.5-nm-thick self-assembled monolayer (SAM). Subsequently, the substrates were rinsed first in 2-propanol then in deionized water and finally in 2-propanol again and dried on a hot plate at 100 °C for 10 min. Thus, the Al_2O_3/SAM dielectric has a capacitance of 185.5 nF cm^{-2}. Gold bottom contacts were deposited by vacuum sublimation (UNIVEX 300, Leybold GmbH; pressure of 1-5 × 10^{-5} mbar, deposition rate of 0.7 Å s^{-1} and nominal thickness of ca. 50 nm monitored by a quartz crystal microbalance) through a shadow mask onto the gate-dielectric substrates at room temperature. Afterwards, the substrates were immersed in a 10 mM solution of pentafluorobenzenethiol (PFBT, Alfa Aesar) in 2-propanol for 30 min, obtaining a SAM on the gold bottom contacts, then rinsed with 2-propanol and dried on a hot plate at 100 °C for 10 minwith N_2 flux. *(R)*-DNTT and *(S)*-DNTT were evaporated in vacuum (pressure of 1-4 × 10^{-6} mbar, deposition rate of 0.1 Å s^{-1} and nominal thickness of ca. 25nm) through a shadow mask onto the substrates which were held at different temperatures, obtaining the final devices with a channel width of 480 µm and channel length of 215 µm. Before the electrical measurements, the samples were left in air and in the dark overnight. OFETs were measured in dark, at ambient air and at room temperature, by using a manual probe station and an Agilent 4156C Semiconductor Parameter Analyzer.
Charge carrier mobilities (µ) and threshold voltages (V_{th}) as a function of the substrate temperature during the evaporation are reported in Table S8. The values of µ were calculated with the conventional gradual channel model approximation using Equation S8 and Equation S9 for the linear and the saturation regime respectively.

$$\mu_{lin} = \frac{\partial I_d}{\partial V_g} \frac{L}{C_i W V_d}$$

Equation S8

$$\mu_{lin} = \left(\frac{\partial I_d}{\partial V_g}\right)^2 \frac{2L}{C_i W}$$

Equation S9

Table S8- Performance of (S)-DNTT, (R)-DNTT and (R+S)-DNTT in bottom-gate bottom-contact OFETs. In the linear regime V_d= -0.1V and in the saturation regime V_d= -4V

	**Substrate T (°C)**	**μ ($cm^2 V^{-1} s^{-1}$)**		**Vth (V)**		**ON/OFF**	
		Linear	Saturation	Linear	Saturation	Linear	Saturation
(R)-DNTT	40	0.51±0.08	0.47±0.08	-1,51	-1,51	2E+04	3E+05
	70	0.48±0.02	0.46±0.02	-1,44	-1,46	2E+04	4E+05
	100	0.27±0.15	0.27±0.15	-1,32	-1,41	2E+04	2E+05
	130	0.16±0.06	0.15±0.05	-1,50	-1,52	4E+03	9E+04
(S)-DNTT	40	0.59±0.01	0.57±0.01	-1.43	-1,46	2E+04	1E+05
	70	0.49±0.01	0.47±0.01	-1.57	-1,60	2E+04	5E+05
	100	0.56±0.02	0.54±0.02	-1.40	-1,43	2E+04	3E+05
	130	0.40±0.04	0.39±0.03	-1.48	-1,49	3E+04	3E+05
(R+S)-DNTT	40	0.27±0.06	0.24±0.01	-1,48	-1,38	1E+04	1E+05
	70	0.26±0.03	0.24±0.03	-1,64	-1,49	2E+03	1E+05
	100	0.26±0.01	0.24±0.02	-1,72	-1,56	9E+03	6E+04
	130	0.28±0.07	0.25±0.07	-1,54	-1,42	2E+03	3E+04

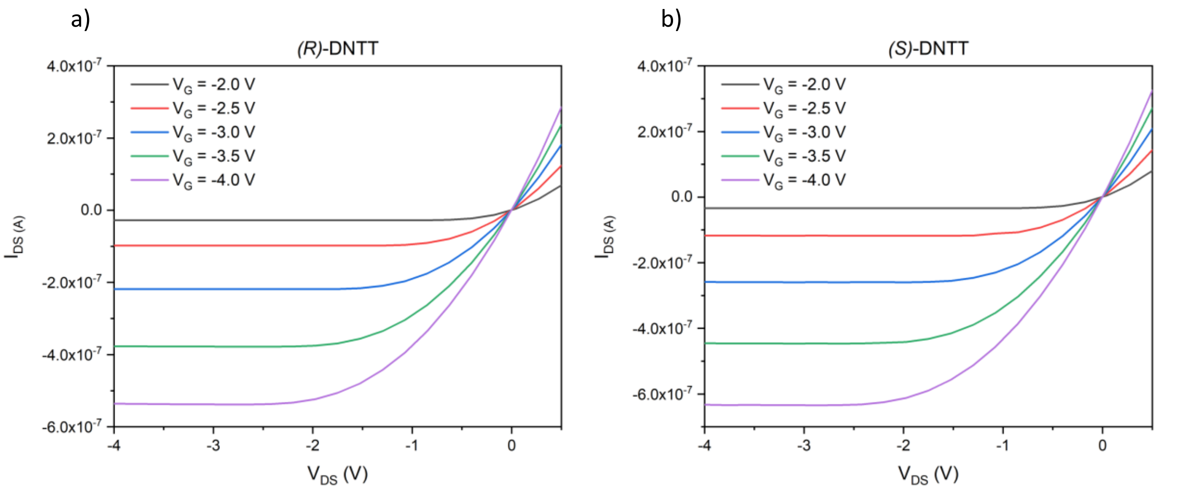

Figure S41 - Output curves of a) (R)-DNTT and b) (S)-DNTT best performing OFETs fabricated with BGBC geometry at a substrate temperature of 40 °C.

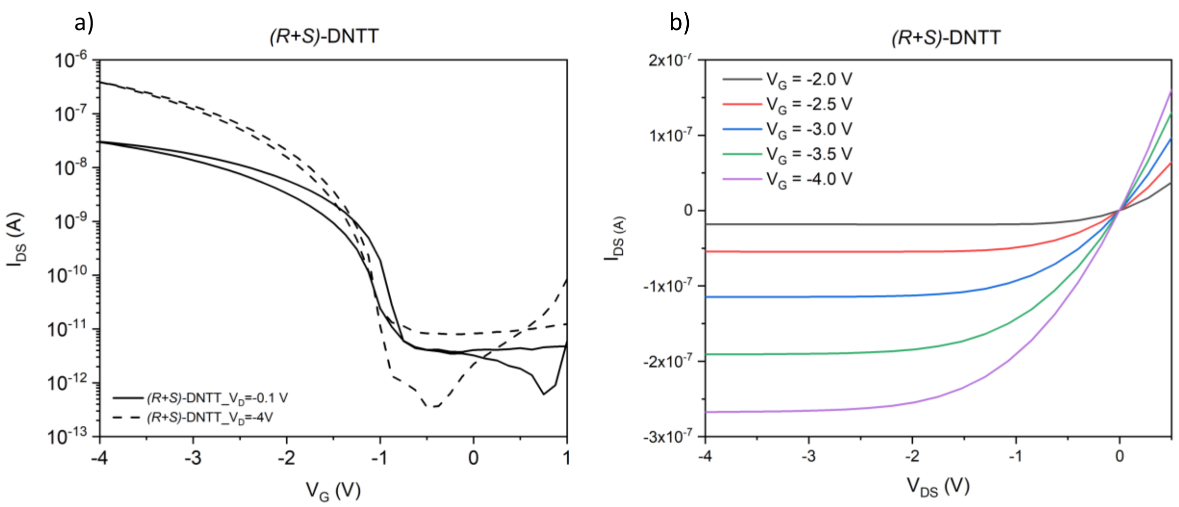

Figure S42 – a) Transfer (solid and dotted lines for linear and saturation regime, respectively) and b) output curves of (R+S)-DNTT best performing OFETs fabricated with BGBC geometry at substrate temperature of 40 °C.

7.2 Thin films characterization

In Figure S43 we report the XRD patterns of thin films (30 nm thinkness) of *(R)*-DNTT and *(S)*-DNTT evaporated on substrates held at different temperatures during the evaporation (40, 70, 100, 130 °C). Thin film XRD were recorded with Panalytical Empyrean diffractometer using Cu–Kα radiation (λ = 1.54178 Å), configuration = 3-axes cradle, scan range 2°-50°, step size = 0.0131°. At low angle, Kiessig fringes are visible beside the expected 00l peaks indicating that unit cell stands on a,b face. PXRD patterns of thin films evaporated at different substrate temperature don't present any difference that could explain the decrease in mobility in OFETs, upon temperature increase, shown in Table S8.

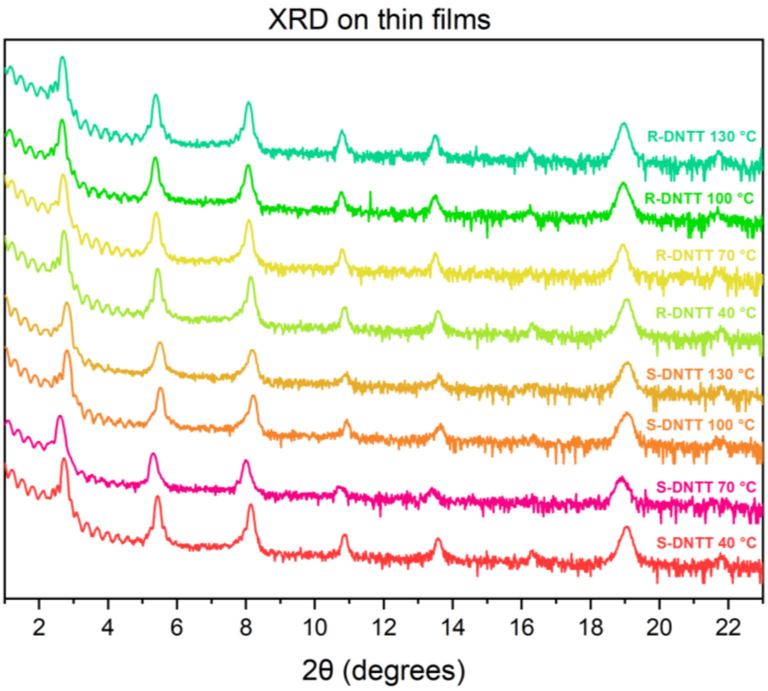

Figure S43 - Thin film XRD of (R)-DNTT and (S)-DNTT evaporated at different substrate temperatures

The same *(R)*-DNTT and *(S)*-DNTT thin films were imaged by AFM (Figure S45). In Figure S is reported the AFM topography of a thin film of *(R+S)*-DNTT, prepared by co-evaporation of equal quantities of the two enantiomers, previously grinded together. AFM topography (height) imaging was performed with a Bruker Dimension Icon setup operating in air, in ScanAsyst mode. For soft OSC materials a probe as the ScanAsyst-AIR is more suitable due to its low stiffness (compared to traditional Tapping/intermittent contact mode) that results in reduced forces applied on the surface. Those probes (tip radius: 2-12 nm, resonance frequency: 70 kHz, force constant: 0.4 N/m) are optimized for this proprietary Bruker mode that collects a force curve at every pixel in the image that is used as feedback signal. The software automatically controls the force applied on the sample by reducing the penetration depth and hence minimizing the interaction with the surface.

Contrary to thin film XRD (Figure S43), AFM images (Figure S45) show different patterns and domains for thin films evaporated at different substrate temperature. This thin film behavior could be related to the differences in mobility upon increasing the temperature from 40 °C to 130 °C.

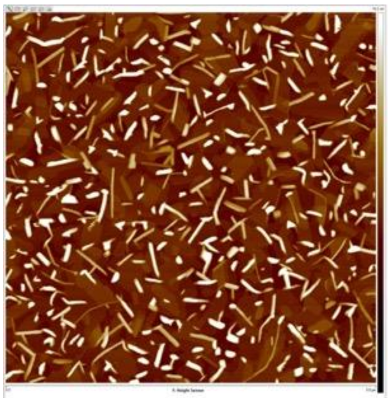

Figure S44 - AFM topography image of a co-sublimated mixture of (R)-DNTT and (S)-DNTT (30 nm thickness) thin film deposited on a TPA/Al_2O_3 at a substrate temperature of 40 °C. Z-scale: 80 nm.

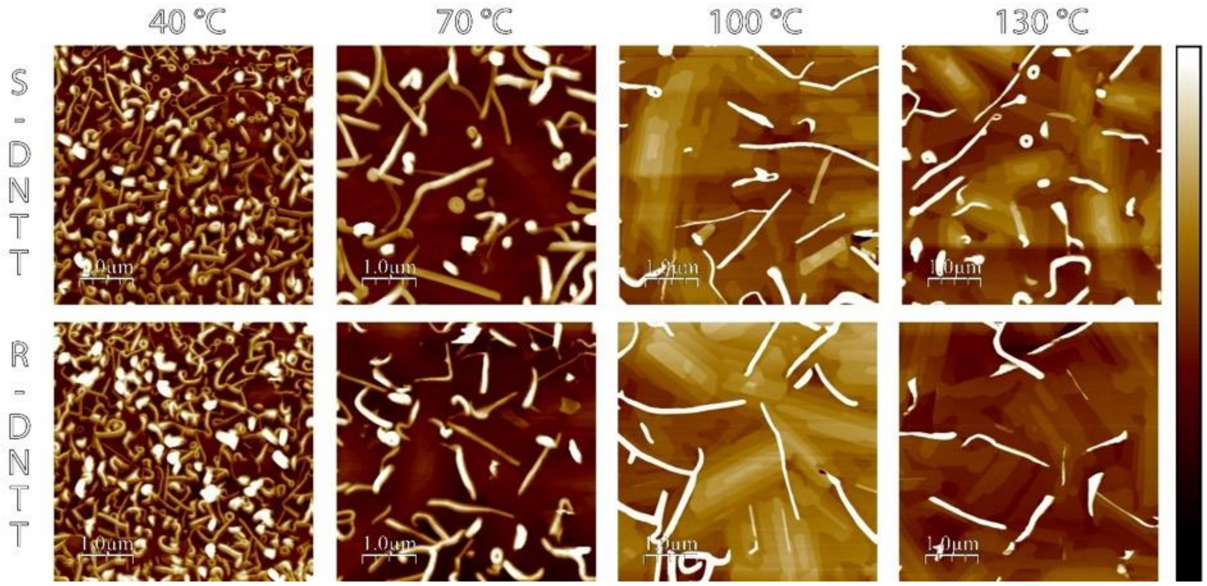

Figure S45 – AFM images of thin films of (S)-DNTT (top) and (R)-DNTT (bottom) (30 nm thickness) deposited at different substrate temperatures (40 °C, 70 °C, 100 °C, 130 °C). The height Z-Scales (Z) for each image (in nm) is: (S-DNTT: 40, Z: 150; 70, Z: 185; 100, Z: 70; 130, Z: 75) and (R-DNTT: 40, Z: 140; 70, Z: 155; 100, Z: 75; 130, Z: 75)

8. Organic Field-Effect Transistors with ferromagnetic contacts

8.1 Device fabrication

For devices with ferromagnetic contacts, Ni with a protective Au thin layer has been used for contacts Ni contacts were deposited onto Al_2O_3/TDPA substrates by vacuum sublimation trough a shadow mask (pressure of 1-5 × 10^{-5} mbar, deposition rate of 0.3 Ǻ s^{-1}, nominal thickness of ca. 40 nm, room temperature). Right after Ni deposition, without opening the vacuum chamber or moving the shadow mask, a thin layer of Au was deposited (pressure of 1-5 × 10^{-5} mbar, deposition rate of 0.1 Ǻ s^{-1}, nominal thickness of ca. 10 nm, room temperature). The contacts were treated with a PFBT solution in 2-propanol for 30 min and then rinsed and dried as described before. *(R)*-DNTT and *(S)*-DNTT were evaporated in vacuum (pressure of 1-4 ×10^{-6} mbar, deposition rate of 0.1 Ǻ s^{-1} and nominal thickness of ca. 25nm) through a shadow mask onto the substrates which were held at 40 °C, obtaining the final devices with a channel width of 480 µm and channel length of 215 µm. Before the electrical measurements, the samples were left in air and in the dark overnight.
The external magnetic field has been applied with two permanent neodymium magnets (~1.3 T) placed at two opposite sides of the substrate carrying the devices, with a resulting magnetic field on the substrate of ~ 0.2 T (Figure S46-a). The homogeneity of the magnetic field has been assessed with iron powder and the effective magnetic field has been measured with a magnetometer (Figure S46-b).

a) b)

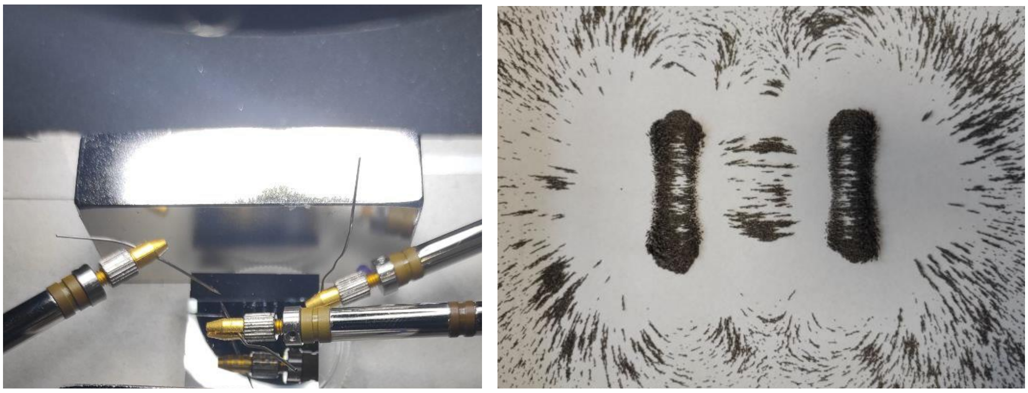

Figure S46 - a) Picture of the magnets set up during measurements and b) corresponding magnetic field lines highlighted by iron powder

9. Structures of molecules mentioned in the text

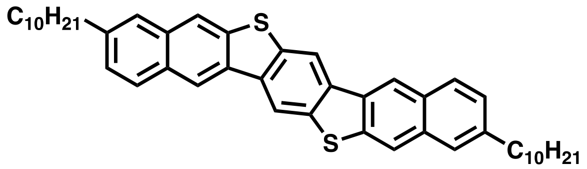

C10-DNBDT

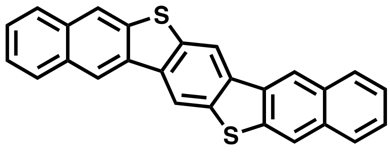

DNBDT

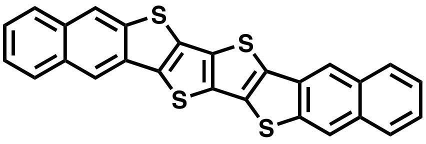

DN4T

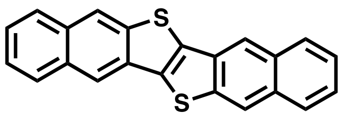

DNTT

10. References

(1) Siu, J. C.; Parry, J. B.; Lin, S. Aminoxyl-Catalyzed Electrochemical Diazidation of Alkenes Mediated by a Metastable Charge-Transfer Complex. *Journal of the American Chemical Society* **2019**, *141*, 2825-2831.

(2) Herrera-Gomez, A.; Bravo-Sanchez, M.; Ceballos-Sanchez, O.; Vazquez-Lepe, M. O. Practical methods for background subtraction in photoemission spectra. *Surface and Interface Analysis* **2014**, *46*, 897-905.

(3) Seah, M. P.; Gilmore, I. S.; Spencer, S. J. Quantitative XPS: I. Analysis of X-ray photoelectron intensities from elemental data in a digital photoelectron database. *Journal of Electron Spectroscopy and Related Phenomena* **2001**, *120*, 93-111.

(4) Takeuchi, R.; Izawa, S.; Hasegawa, Y.; Tsuruta, R.; Yamaguchi, T.; Meissner, M.; Ideta, S.-i.; Tanaka, K.; Kera, S.; Hiramoto, M.; Nakayama, Y. Experimental Observation of Anisotropic Valence Band Dispersion in Dinaphtho[2,3-b:2′,3′-f]thieno[3,2-b]thiophene (DNTT) Single Crystals. *The Journal of Physical Chemistry C* **2021**, *125*, 2938-2943.

(5) Dierking, I.: *Textures of liquid crystals*; John Wiley & Sons, 2003.

(6) CrysalisPRO. 2016. Rigaku Oxford Diffraction Ltd., Yarnton, England.

(7) G. M. Sheldrick, Acta Cryst C 2015, 71, 3.

(8) L. J. Farrugia, J Appl Cryst 2012, 45, 849

(9) Spackman, M. A.; Jayatilaka, D. Hirshfeld surface analysis. *CrystEngComm* **2009**, *11*, 19-32.

(10) Spackman, P. R.; Turner, M. J.; McKinnon, J. J.; Wolff, S. K.; Grimwood, D. J.; Jayatilaka, D.; Spackman, M. A. CrystalExplorer: A program for Hirshfeld surface analysis, visualization and quantitative analysis of molecular crystals. *Journal of Applied Crystallography* **2021**, *54*, 1006-1011.

(11) Mathematical, Physical and Chemical Tables, 3. ed., repr. with corrections.; Prince, E., Internationale Union für Kristallographie, Eds.; International tables for crystallography; Kluwer Acad. Publ: Dordrecht, 2011

(12) McKinnon, J. J.; Spackman, M. A.; Mitchell, A. S. Novel tools for visualizing and exploring intermolecular interactions in molecular crystals. *Acta Crystallographica Section B: Structural Science* **2004**, *60*, 627-668.

(13) Frisch, M. J.; Trucks, G. W.; Schlegel, H. B.; Scuseria, G. E.; Robb, M. A.; Cheeseman, J. R.; Scalmani, G.; Barone, V.; Petersson, G. A.; Nakatsuji, H.; Li, X.; Caricato, M.; Marenich, A. V.; Bloino, J.; Janesko, B. G.; Gomperts, R.; Mennucci, B.; Hratchian, H. P.; Ortiz, J. V.; Izmaylov, A. F.; Sonnenberg, J. L.; Williams; Ding, F.; Lipparini, F.; Egidi, F.; Goings, J.; Peng, B.; Petrone, A.; Henderson, T.; Ranasinghe, D.; Zakrzewski, V. G.; Gao, J.; Rega, N.; Zheng, G.; Liang, W.; Hada, M.; Ehara, M.; Toyota, K.; Fukuda, R.; Hasegawa, J.; Ishida, M.; Nakajima, T.; Honda, Y.; Kitao, O.; Nakai, H.; Vreven, T.; Throssell, K.; Montgomery Jr., J. A.; Peralta, J. E.; Ogliaro, F.; Bearpark, M. J.; Heyd, J. J.; Brothers, E. N.; Kudin, K. N.; Staroverov, V. N.; Keith, T. A.; Kobayashi, R.; Normand, J.; Raghavachari, K.; Rendell, A. P.; Burant, J. C.; Iyengar, S. S.; Tomasi, J.; Cossi, M.; Millam, J. M.; Klene, M.; Adamo, C.; Cammi, R.; Ochterski, J. W.; Martin, R. L.; Morokuma, K.; Farkas, O.; Foresman, J. B.; Fox, D. J.: Gaussian 16 Rev. C.01. Wallingford, CT, 2016.

(14) te Velde, G.; Bickelhaupt, F. M.; Baerends, E. J.; Fonseca Guerra, C.; van Gisbergen, S. J. A.; Snijders, J. G.; Ziegler, T. Chemistry with ADF. *Journal of Computational Chemistry* **2001**, *22*, 931-967.

(15) D. B. Boyd, K. B. Lipkowitz, Reviews in Computational Chemistry. Vol. 15 Vol. 15, Wiley-VCH, New York, New York, 2000
